# Laparoscopy is non‐inferior to open surgery for rectal cancer: A systematic review and meta‐analysis

**DOI:** 10.1002/cam4.7363

**Published:** 2024-07-05

**Authors:** Ling Ma, Hai‐jiao Yu, Yu‐bing Zhu, Wen‐xia Li, Kai‐yu Xu, Ai‐min Zhao, Lei Ding, Hong Gao

**Affiliations:** ^1^ Department of Gastrointestinal Tumor Surgery Beijing Shijitan Hospital Affiliated to Capital Medical University Beijing People's Republic of China

**Keywords:** laparoscopic surgery, meta‐analysis, non‐inferiority, open surgery, rectal cancer

## Abstract

**Background:**

Laparoscopic surgery has been endorsed by clinical guidelines for colon cancer, but not for rectal cancer on account of unapproved oncologic equivalence with open surgery.

**Aims:**

We started this largest‐to‐date meta‐analysis to comprehensively evaluate the safety and efficacy of laparoscopy in the treatment of rectal cancer compared with open surgery.

**Materials & Methods:**

Both randomized and nonrandomized controlled trials comparing laparoscopic proctectomy and open surgery between January 1990 and March 2020 were searched in PubMed, Cochrane Library and Embase Databases (PROSPERO registration number CRD42020211718). The data of intraoperative, pathological, postoperative and survival outcomes were compared between two groups.

**Results:**

Twenty RCTs and 93 NRCTs including 216,615 patients fulfilled the inclusion criteria, with 48,888 patients received laparoscopic surgery and 167,727 patients underwent open surgery. Compared with open surgery, laparoscopic surgery group showed faster recovery, less complications and decreased mortality within 30 days. The positive rate of circumferential margin (RR = 0.79, 95% CI: 0.72 to 0.85, *p* < 0.0001) and distal margin (RR = 0.75, 95% CI: 0.66 to 0.85 *p* < 0.0001) was significantly reduced in the laparoscopic surgery group, but the completeness of total mesorectal excision showed no significant difference. The 3‐year and 5‐year local recurrence, disease‐free survival and overall survival were all improved in the laparoscopic surgery group, while the distal recurrence did not differ significantly between the two approaches.

**Conclusion:**

Laparoscopy is non‐inferior to open surgery for rectal cancer with respect to oncological outcomes and long‐term survival. Moreover, laparoscopic surgery provides short‐term advantages, including faster recovery and less complications.

## 1 INTRODUCTION

Colorectal cancer (CRC) is the third most common cause of cancer death in the United States with an incidence rate of 35.3 and mortality rate of 13.2 per 100,000 population. Approximately one‐third of these cases are rectal cancers.^1^, ^2^ The mainstay treatment for rectal cancer remains surgical resection. Considered as a major landmark, laparoscopic colectomy was first performed on 20 patients by Jacobs et al.^3^ Over the next 30 years, the theory and technique of laparoscopic surgery in the management of rectal cancer have been standardized and improved enormously. Since 2006, the National Comprehensive Cancer Network (NCCN) clinical practice guidelines had recommended laparoscopic‐assisted colectomy to be the priority option for the qualified cases. However, the application of laparoscopy in rectal cancer is still controversial.

Laparoscopic surgery has unbeatable advantages in terms of postoperative recovery. The major controversies over laparoscopy versus open proctectomy are on the oncological outcomes and long‐term survival. The early researches showed doubts on the high incidence of postoperative and peritoneal implantation metastasis after laparoscopic surgery.^4^ On the contrary, accumulating clinical trials had proved the safety and efficacy of laparoscopic proctectomy compared with open surgery. However, there had been no comprehensive assessment and comparison covering all the aspects of the two procedures in the treatment of rectal cancer, especially the pathological outcomes and long‐term survival. Based on a comprehensively review of literature, we started this largest‐to‐date meta‐analysis to make an overall comparison between the two surgical methods.

## 2 METHODS AND METHODS

The protocol for this meta‐analysis was available in PROSPERO (CRD42020211718).

### 2.1 Literature search

We searched literatures involved with randomized controlled trials (RCTs) or nonrandomized controlled trials (NRCTs) on the following online databases: MEDLINE (through PubMed), Cochrane Library and Embase Databases, covering a period from January 1990 to March 2020. The search string was as follows: (rectal cancer or rectal carcinoma or rectal neoplasms) and (treatment or therapy or access or approach or management) and (laparoscopy or laparoscopic surgery) and (open surgery or laparotomy). Manual retrieval of relevant literature reference was available to expand the search and to ensure that no research was omitted. Only full‐text English‐language trials that met the selection criteria were retrieved and reviewed.

### 2.2 Inclusion criteria

The trials were included based on the following inclusion criteria: (1) RCTs or NRCTs conducted in the period from January 1990 to March 2020; (2) the population of interest were adults diagnosed with rectal cancer by pathology of histology and underwent surgical treatment by means of laparoscopic proctectomy or open surgery; the surgical procedure of protectomy including anterior resection, abdominoperineal resection and intersphincteric resection was made according to the tumor localization above the anal verge, extent of tumor invasion and histologic type; (3) the selected literature must include two sets of data comparison of laparoscopic surgery group and open surgery group in the following aspects: surgical process, pathological results, postoperative recovery and short‐term or long‐term outcomes; and (4) literatures were full‐text papers and published in English.

### 2.3 Exclusion criteria

The trials were excluded based on the following exclusion criteria: (1) articles were not written in English, or unable to provide full text; (2) review, editorials and commentary articles; (3) literatures were published by the same researcher or research institutes; and (4) data provided by the paper were not clear and valid or could not be obtained via calculation.

### 2.4 Quality assessment

The quality of trials was assessed independently by two authors (LI and XU) using two methods. All RCTs were assessed by the Cochrane risk of bias criteria,^5^ whereas the NRCTs were assessed by the Newcastle‐Ottawa Scale (NOS),^6^ as recommended in the Cochrane Handbook. A score above 6 indicated high quality; otherwise, a lower score indicated poor quality. If controversy existed between the two independent evaluations, all of the authors participated in a discussion to resolve the issue.

### 2.5 Data extraction

All the information about the participants' characteristics, surgical process, pathological results, postoperative recovery and complications, and short‐term or long‐term outcomes from included studies were extracted independently by three authors (MA, YU, and ZHU). Consensus was reaching after discussion by the three authors or the intervene of the senior authors (DING and GAO).

### 2.6 Statistical analysis

This meta‐analysis was performed using Review Manager (RevMan‐Version 5.3.). We designed subgroup analysis based on the RCTs and NRCTs. Continuous variables were pooled using the mean difference (MD) with a 95% confidence interval (CI). Discontinuous variables were pooled using the risk ratios (RRs) with a 95% CI. If studies reported the median, range and size of the trial, the means and standard deviations were calculated according to Hozo et al.^7^ If studies only reported the medians and range, this parameter would be excluded. According to the practice recommendation of the Cochrane Handbook, trials with zero events in both the intervention and the control groups were not included in the meta‐analysis.

All tests were two‐tailed, and *p* < 0.05 was considered statistically significant. The *I* ^2^ statistic was used to investigate the heterogeneity among the studies. *I* ^2^ < 50% indicated a small inconsistency and the fixed effects model was used to pool the data. *I* ^2^ > 50% indicated a large inconsistency and the random effects model was used to pool the data. Sensitivity analysis was performed by excluding low‐quality studies, trials recruiting participants with particular conditions, or trials with characteristics different from the others.

## 3 RESULTS

### 3.1 Study Selection

**FIGURE 1:**
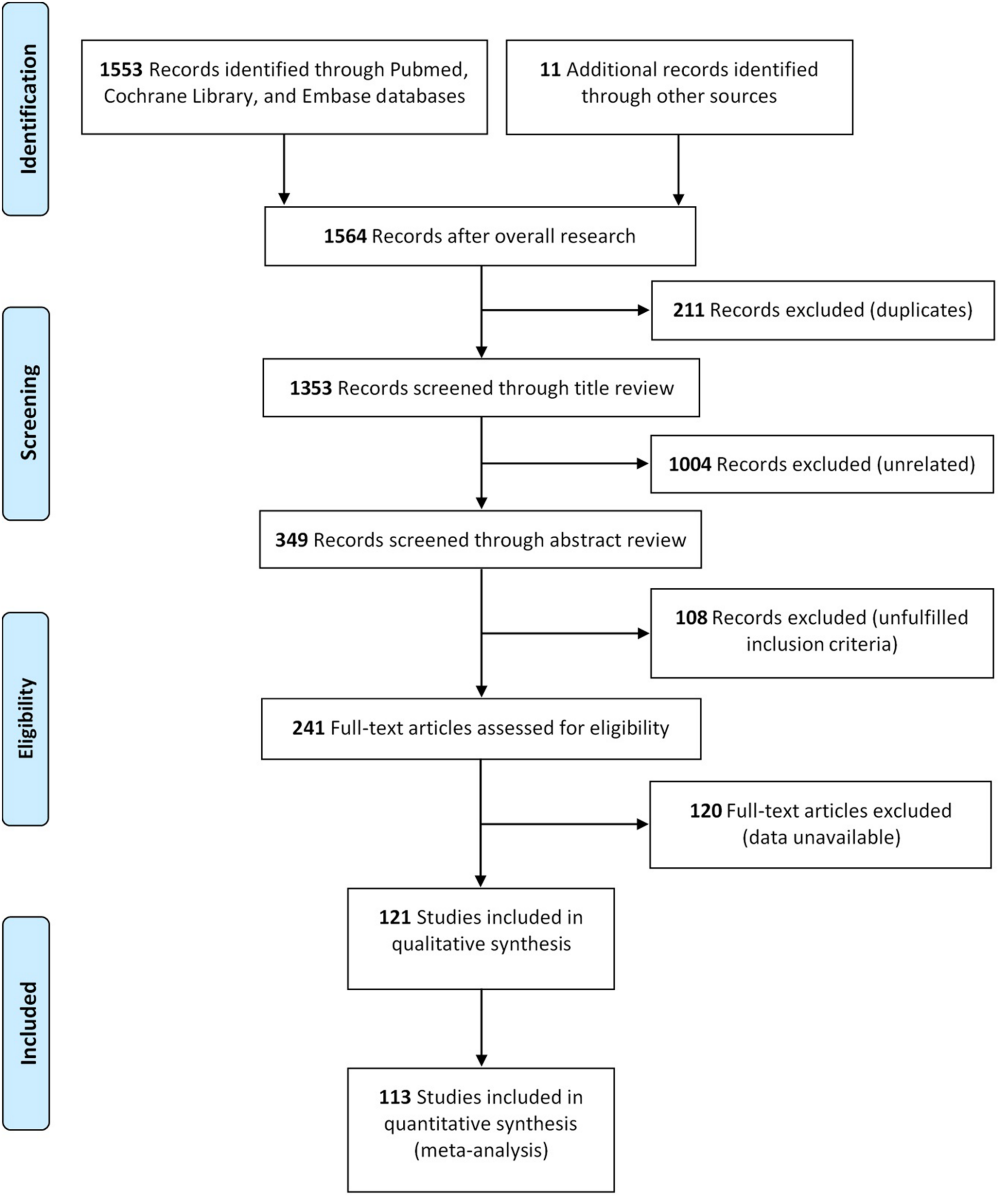
Flow diagram of literature search and selection process.

After the combined search, 1564 articles were identified. Titles and abstracts of these records were screened for inclusion. 1323 articles were rejected and the remaining 241 articles then underwent full‐text evaluation. Finally, 113 articles including 20 RCTs and 93 NRCTs met the inclusion criteria. The PRISMA flowchart for study inclusion and exclusion process was showed in Figure 1.

### 3.2 Characteristics and methodological quality of eligible studies

The characteristics of the 113 studies were summarized in Table 1. It included 20 RCTs and 93 NRCTs for a total of 216,615 rectal cancer patients. Of them, 48,888 patients received laparoscopic proctectomy and 167,727 patients underwent open surgery. In the laparoscopic surgery group, patients required a conversion from laparoscopy to open surgery. The conversion cases remained in the laparoscopic surgery group according to the principle of intention‐to‐treat.

**TABLE 1:** Summary of the Included Studies.

Reference	Area	Study type	Period	Inclusion criteria	Medical center included	Number of participants	Surgical approach		Conversion rate (%)	Neoadjuvant treatment	Follow up time (month)	
							Lap (*n*)	Open (*n*)		Lap/open	Lap	Open
Araujo^8^	Brazil	RCT, P	September 1997–September 2000	Distal rectal cancer with nCRT	Single	28	13	15	0	15/13	47.2^a^	47.2^a^
Leung^9^	Hong Kong, China	RCT, P	September 1993–October 2002	Rectosigmoid cancer	Single	403	203	200	23.2	55/77	52.7 (38.9)^c^	49.2 (35.4)^c^
Zhou^10^	China	RCT, P	June 2001–September 2002	Low rectal cancer, within 1.5–8 cm from DL	Single	171	82	89	ND	ND	(1–16)^b^	(1–16)^b^
CLASICC^11^, ^12^, ^13^, ^14^	UK	RCT, P	July 1996–July 2002	Colorectal cancer	27	381	253	128	0	ND	62.9 (22.9–92.8)^c^	62.9 (22.9–92.8)^c^
Braga^15^	Italy	RCT, P	ND	Rectal cancer	Single	168	83	85	7.2	14/12	53.6^a^	53.6^a^
Pechlivanides^16^	Greece	RCT, P	ND	Mid and low rectal cancer, within 12 cm from AV	Single	73	34	39	3	13/17	ND	ND
Ng^17^	Hong Kong, China	RCT, P	July 1994–February 2005	Low rectal cancer, within 5 cm from AV	Single	99	51	48	9.8	0/0	87.2 (22.8–150.0)^b^	90.1 (27.0–145.5)^b^
Lujan^18^	Spain	RCT, P	January 2002–February 2007	Mid and low rectal cancer	Single	204	101	103	7.9	73/77	32.8 (18.9)^a^	34.1 (20.0)^a^
Ng^19^	Hong Kong, China	RCT, P	September 1993–October 2002	Rectosigmoid junction and upper rectal cancer, within 12–15 cm from AV	Single	153	76	77	30.3	24/46	112.5 (71.1–168.3)^b^	108.8 (69.8–168.7)^b^
COREAN^20^, ^21^	Korea	RCT, P	April 2006–August 2009	cT3N0–2M0 mid or low rectal cancer, after nCRT	3	340	170	170	1.18	170/170	47.65 (37–56)^c^	46.35 (38–57)^c^
Liu^22^	China	RCT, P	February 2005–October 2008	Rectal cancer	Single	186	98	88	0	ND	16.3^a^	16.3^a^
Liang^23^	China	RCT, P	May 2004–April 2008	Nonmetastatic rectal cancer without adjuvant therapy	Single	343	169	174	0.59	0/0	44 (1–72)^b^	44 (1–72)^b^
Gong^24^	China	RCT, P	September 2008–July 2011	Mid and low rectal cancer	Single	138	67	71	2.99	ND	21 (9–56)^b^	21 (9–56)^b^
COLOR II^25^, ^26^	Belgium et al	RCT, P	January 2004–May 2010	Rectal cancer, within 15 cm from AV, without evidence of distant metastases	30	1044	699	345	17	608/298	36	36
Enroll^27^	UK	RCT, P	July 2008–April 2012	Rectal cancer (colorectal cancer)	12	56	29	27	8	ND	12	12
Ng^28^	Hong Kong, China	RCT, P	August 2001–August 2007	Mid and low rectal cancer	Single	80	40	40	7.5	ND	75.7 (16.9–115.7)^b^	76.1 (4.7–126.6)^b^
ALaCaRT^29^, ^30^	Australia	RCT, P	March 2010–November 2014	T1‐T3 rectal cancer, within 15 cm from AV	24	473	238	235	9	119/116	38.4 (36–49.2)^c^	39.6 (36–50.4)^c^
Z6051^31^	USA and Canada	RCT, P	October 2008–September 2013	Stage II or III rectal cancer within 12 cm from AV	35	462	240	222	11.3	240/222	47.7 (26.1–59.1)^c^	48.1 (33.9–59.8)^c^
Yao^32^	China	RCT, P	May 2014–February 2016	Rectal cancer with nCRT	Single	120	60	60	0	60/60	1	1
Alhanafy^33^	Turkey	RCT, P	February 2017–February 2019	Rectal cancer	Single	120	60	60	8.33	30/27	1	1
Schwandner^34^	Germany	NRCT, R, CCS	September 1992–January 1998	Rectosigmoid or rectal cancer	Single	64	32	32	0	ND	33.1 (24.2)^a^	31.0 (17.9)^a^
Leung^35^	Hong Kong, China	NRCT, R	January 1993–January 1996	Low rectal cancer	Single	59	25	34	4	ND	30.1 (6.0–52.3)^b^	28.3 (1.9–55.1)^b^
Hartley^36^	UK	NRCT	December 1993–October 1998	Rectal cancer	Single	43	21	22	0	ND	38 (6–53)^b^	38 (6–53)^b^
Anthuber^37^	Germany	NRCT, R	January 1996–March 2002	Rectal cancer	Single	435	101	334	10.9	27/116	17^b^	17^b^
Feliciotti^38^	Italy	NRCT, R	May 1992–April 2002	Rectal cancer	Single	124	81	43	12.3	75/38	43.8 (12–108)^d^	43.8 (12–108)^d^
Wu^39^	China	NRCT, R, CCS	April 2002–May 2003	Rectal cancer	Single	36	18	18	0	0/0	1	1
Bretagnol^40^	France	NRCT, R, CCS	September 2000–September 2004	Mid and low rectal cancer, within 12 cm from AV	2	288	144	144	14	120/115	18 (1–46)^b^	18 (1–46)^b^
Breukink^41^	Netherlands	NRCT, R, CCS	April 1996–March 2003	Rectal cancer after nCRT	Single	82	41	41	10	41/41	14 (2–31)^d^	14 (2–31)^d^
Morino^42^	Italy	NRCT, P, CCS	April 1994–April 2002	Mid and low rectal carcinoma, within 12 cm from AV	Single	191	98	93	18.4	ND	46.3 (12–132)^d^	49.7 (12–132)^d^
Sahakitrungruang^43^	Thailand	NRCT, R	June 2004–May 2005	Rectosigmoid or rectal cancer	Single	49	24	25	8.3	ND	1	1
Law^44^	Hong Kong, China	NRCT, R	June 2000–December 2004	Upper and mid cancer, within 8–20 cm from AV	Single	265	98	167	12.2	ND	21.2 (4.1–56.7)^b^	21.2 (4.1–56.7)^b^
Wong^45^	Hong Kong China	NRCT, P	March 1994–December 2003	Low rectal and anal canal cancer	Single	102	71	31	15.5	6/0	46 (6–100)^b^	50 (6–123)^b^
Lelong^46^	France	NRCT, P, CCS	January 1998–September 2004	Rectal cancer	Single	172	104	68	14.5	ND	3	3
Staudacher^47^	Italy	NRCT	January 1998–September 2005	Mid and low rectal cancer	Single	187	108	79	12	68/34	27.6 (6–82)^b^	27.6 (6–82)^b^
Veenhof^48^	Netherlands	NRCT, R, CCS	February 1999–November 2005	Rectal cancer	Single	100	50	50	8	26/26	17 (6–26)^c^	22 (10–38)^c^
Ströhlein^49^	Germany	NRCT, P	1998–2005	Rectal cancer	2	389	114	275	21.9	56/174	31.1 (2–71)^d^	32.6 (1–76)^d^
Ding^50^	China	NRCT, R, CCS	January 2004–December 2005	RECTAL cancer	3	335	115	220	11.3	ND	20 (11)^c^	20 (11)^c^
González^51^	Mexico	NRCT, P	November 2005–November 2007	Mid and low rectal cancer	Single	56	28	28	0	ND	(9–24)^b^	(9–24)^b^
Gouvas^52^	Greece	NRCT, R, CCS	January 1998–March 2007	Mid and low rectal cancer, within 10 cm from AV	4	88	45	43	9	15/11	3	3
Khaikin^53^	America	NRCT, R	November 2004–July 2006	Rectal cancer	Single	82	32	50	12.5	18/31	10 (1–18)^b^	10 (1–18)^b^
Kim^54^	Korea	NRCT, R	1994–2004	Rectal cancer	Single	407	272	135	4	ND	52 (25–129)^b^	180 (29–263)^b^
Koulas^55^	Greece	NRCT, R	October 1998–December 2006	Rectal cancer	Single	117	57	60	7.01	ND	38^a^	78^a^
Laurent^56^	France	NRCT, R	1994–2006	Rectal cancer, within 15 cm from AV	Single	471	238	233	15.1	178/149	52 (1–151)^b^	52 (1–151)^b^
Law^57^	Hong Kong China	NRCT, R	June 2000–December 2006	Rectal cancer	Single	421	111	310	12.5	7/47	34^b^	34^b^
Biondo^58^	Spain	NRCT, R	May 2006–May 2008	Rectal cancer	7	604	209	395	ND	138/231	1	1
Denoya^59^	America	NRCT, R, CCS	1997–2005	Mid and low rectal cancer after nCRT	Single	64	32	32	28	32/32	1	1
Feng^60^	China	NRCT, R	January 2003–May 2005	Rectal cancer	Single	173	87	86	0	0/0	50 (36–65)^b^	49 (36–64)^b^
Fujimoto^61^	Japan	NRCT, R	July 2005–December 2008	Very low rectal cancer, below 5 cm from dentate line	Single	54	35	19	3	11/6	19 (5–42)^b^	19 (5–42)^b^
Baik^62^	America	NRCT, R, CCS	September 2001–September 2009	Rectal cancer, within 12 cm from AV	Single	162	54	108	11.1	43/89	59^b^	59^b^
da Luz Moreira^63^	USA	NRCT, R, CCS	1992–2008	Rectal cancer	Single	182	91	91	18.6	30/30	40 (7–179)^b^	40 (7–179)^b^
Greenblatt^64^	America	NRCT, R	2005–2009	Rectal cancer	Multiple	5420	1040	4380	ND	377/2002	1	1
June^65^	Korea	NRCT, R	December 2007–July 2009	Rectal cancer	Single	211	123	88	0	10/9	1	1
Li^66^	China	NRCT, P	January 2000–June 2005	Mid and low rectal cancer, within 10 cm from AV	Single	236	113	123	5.3	39/46	76 (30.3)^a^	73.6 (30.5)^a^
Park^67^	Japan and Korea	NRCT, R	January 1997–December 2009	Low rectal cancer	2	210	130	80	0	10/1	32.5 (16–41.5)^c^	37 (25–45.2)^c^
Yamamoto^68^	Japan	NRCT, P, CCS	July 2002–January 2011	Low rectal cancer	Single	44	22	22	0	0/0	1	1
Gunka^69^	Czech Republic	NRCT, P	January 2001–December 2006	Nonmetastatic rectal cancer	Single	145	75	70	6.7	23/20	65 (25–96)^b^	65 (25–96)^b^
Jefferies^70^	UK	NRCT, R	February 2007–June 2010	Rectal cancer	Single	41	16	25	12.5	7/14	23 (6–46)^b^	23 (6–46)^b^
Kellokumpu^71^	Finland	NRCT, P	January 1999–December 2006	Rectal cancer	Single	191	100	91	22	67/71	57.6	57.6
Laurent^72^	France	NRCT, R	1990–2007	Rectal cancer, below 6 cm from AV	Single	175	110	65	22	103/55	53 (1–170)^b^	53 (1–170)^b^
Li^73^	China	NRCT, R	June 2004–June 2010	Stage I‐III rectal cancer	Single	657	381	276	ND	ND	51 (13–87)^d^	51 (13–87)^d^
Mckay^74^	Australia	NRCT, R	January 2001–December 2008	Rectal cancer	Multiple	545	157	388	8.3	77/137	1	1
Seshadri^75^, ^76^	India	NRCT, P, CCS	January 2004–January 2010	Mid and low rectal cancer, clinical stage cT2‐4 or cN1‐2 after nCRT	Single	144	72	72	4	72/72	69.5 (1–138)^b^	69.5 (1–138)^b^
Siani^77^	Roma	NRCT, R	January 2004–January 2010	Stage I‐III mid and low rectal cancer	Single	60	30	30	3.3	30/30	38.3^a^	37.9^a^
Kuo^78^	Taiwan, China	NRCT, R	January 2006–October 2011	Very low rectal cancer, within 5 cm from AV	Single	58	28	30	0	28/27	1	1
Lee^79^	Korea	NRCT, R	June 2001–December 2008	Stage I rectal cancer	Single	160	80	80	1.25	0/0	51 (1–109)^b^	51 (1–109)^b^
Lujan^80^	Spain	NRCT, P	2006–July 2010	Rectal cancer	Single	4405	1387	3018	17.37	806/1582	23.58 (11.77)^a^	21.98 (12.39)^a^
Park^81^	Korea	NRCT, R, CCS	January 2000–November 2008	Rectal cancer, within 12 cm from AV	Single	812	406	406	2	43/33	55.5 (35.4–77.4)^c^	55.5 (35.4–77.4)^c^
Penninckx^82^	Belgium	NRCT, R	January 2006–October 2011	Mid or low rectal cancer	Multiple	2660	764	1896	11.5	508/1393	36	36
Yang^83^	China	NRCT, R	May 2010–May 2012	Low rectal cancer, within 10 cm from AV	Single	177	87	90	1.1	ND	1	1
Ferko^84^	Czech Republic	NRCT, P	January 2010–December 2012	Mid and lower rectal cancer, within 10 cm from AV	Single	125	53	72	6.2	39/58	ND	ND
Inada^85^	Japan	NRCT, R, CCS	Aug 2004–November 2011	Low rectal cancer	Single	28	14	14	0	ND	1	1
Inomata^86^	Japan	NRCT, R	April 2002–March 2012	Stage II/III rectal cancer	Single	65	38	27	ND	5/1	1	1
Keller^87^	USA	NRCT, R	Aug 2005–May 2011	Rectal cancer	Single	81	62	19	14.5	40/16	25 (0.7–70.3)^b^	25 (0.7–70.3)^b^
Kusano^88^	Japan	NRCT, R	February 2002–November 2012	Low rectal cancer after nCRT	Single	33	19	14	ND	19/14	35^b^	40^b^
Wang^89^	China	NRCT, R	January 2010–January 2012	Low rectal cancer	Single	236	100	136	2	0/0	26 (17–38)^d^	26 (17–38)^d^
Zhou^90^	China	NRCT, R	January 2005– January 2008	Rectal cancer, within 12 cm from AV	Single	122	57	65	8.8	33/40	56.6 (10–84)^d^	56.6 (10–84)^d^
Chi^91^	China	NRCT, R	January 2006–Aug 2013	Low rectal cancer	Single	137	89	48	2.25	28/18	32.3^b^	32.3^b^
Guo^92^	China	NRCT, R, CCS	April 2007–December 2013	Rectal cancer	Single	382	191	191	ND	ND	42	46
Huang^93^	China	NRCT, CCS, R	January 2006–December 2013	Rectal cancer	Single	916	492	424	2.44	ND	55^a^	55^a^
Kim^94^	Korea	NRCT, R	January 2002–December 2011	Rectal cancer	Single	307	131	176	ND	14/26	40 (11–135)^b^	51 (8–142)^b^
Li^95^	China	NRCT, P	January 2003–December 2008	Mid and low rectal cancer, within 10 cm from AV	Single	281	129	152	6.2	48/55	74.8 (31.2)^a^	73.9 (31.7)^a^
Nussbaum,^96^	USA	NRCT, R, CCS	2010–2011	Rectal cancer	Multiple	12,860	6430	6430	ND	1978/1961	1	1
Wang,^97^	China	NRCT, R	March 2009–December 2013	Rectal cancer, within 10 cm from AV	Single	212	106	106	2.83	26/26	16 (1–67)^b^	16 (1–67)^b^
Zeng^98^	China	NRCT, R	June 2007–June 2012	Rectal cancer	Single	294	112	182	6.3	39/61	29 (1–73)^b^	29 (1–74)^b^
Zhou^99^	China	NRCT, R	January 2000–December 2009	Stage II‐III rectal cancer	Single	406	152	254	4.6	13/16	63 (28–112)^b^	65 (32–118)^b^
Allaix^100^	Italy	NRCT, R	April 1994–Aug 2005	Rectal cancer, within 12 cm from AV	Single	307	153	154	10.5	78/85	79 (12–231)^b^	82.5 (12–242)
Chen ZX^101^	China	NRCT, CCS, R	2007–2013	Rectal cancer, over 75 years old	Single	74	37	37	0	0	1	1
Chen WP^102^	China	NRCT, R	June 2009–October 2015	Mid‐low rectal cancer following nCRT	Single	172	75	97	0	75/97	1	1
Keskin^103^	Turkey	NRCT, R	January 2005–December 2011	Rectal cancer	Single	587	437	150	7.78	286/81	48.5 (24.1)^a^	48.5 (24.1)^a^
Kim^104^	Korea	NRCT, R	July 2010–February 2015	Rectal cancer	Single	1581	486	1095	5.1	61/553	36	36
Liu^105^	China	NRCT, P	April 2009–April 2013	Low or ultra‐low rectal cancer, diameter <2 cm, located at 3–5 cm from AV	Single	112	67	45	0	0	(26–40)^b^	(24–38)^b^
Nonaka^106^	Japan	NRCT, R	January 2008– December 2014	Advanced mid and low rectal cancer (pStage II‐III), within 10 cm from AV	Single	78	40	38	0	14/13	32 (5–53)^b^	51 (4–66)^b^
Odermatt^107^	UK	NRCT, CCS, R	January 2003–June 2013	Low rectal cancer	Single	96	48	48	4.17	7/8	55 (24–72)^c^	55 (24–72)^c^
De'Angelis^108^	France	NRCT, CCS, R	January 2005–December 2015	pT4 rectal cancer	Four	104	52	52	21.2	35/36	34.37 (23.09)^a^	43.81 (32.54)^a^
Matsuhashi^109^	Japan	NRCT, CCS, R	July 2008–April 2013	Low rectal cancer, ISR	Single	25	19	6	10.5	0	54	54
Silva‐Velazco^110^	USA	NRCT, P	January 2010–December 2014	Rectal cancer	Single	422	118	304	15.4	58/124	1	1
Wu^111^	China	NRCT, R	October 2010–December 2015	Rectal cancer	Single	233	112	121	4.46	0	36	36
Andersen^112^	Denmark	NRCT, R	January 2007–December 2013	Rectal cancer	Multiple	6189	3099	3090	ND	ND	40.8 (28.8–60)^c^	54 (27.6–69.6)^c^
Aydın^113^	Turkey	NRCT, R	January 2006–January 016	Rectal cancer	Single	121	71	50	ND	19/4	55.2^b^	56.75^b^
Chen^114^	Taiwan, China	NRCT, CCS, R	2008–2012	Rectal cancer	Multiple	108,436	5578	102,858	ND	ND	1	1
Draeger^115^	Germany	NRCT, R	January 2004–December 2013	Rectal cancer	Single	1507	428	1079	ND	186/398	85.2^b^	85.2^b^
Garfinkle^116^	Canada	NRCT, R	January 2009–March 2012	Rectal cancer	2	235	34	201	17	7/39	1	1
Hida^117^	Japan	NRCT, CCS, R	January 2010–2014	Low rectal cancer, clinical stage II and III	69	964	482	482	5.2	169/167	39.6^b^	39.6^b^
Tayar^118^	Brazil	NRCT, CCS, R	December 2008–December 2012	Rectal cancer	Single	100	50	50	ND	43/42	1	1
Wu^119^	China	NRCT, R	January 2009–December 2013	Rectal cancer	Single	891	277	614	1.3	57/42	65 (46–77)^c^	65 (46–77)^c^
Chen^120^	Taiwan, China	NRCT, R	July 2008–April 2018	Low rectal cancer, within 7 cm from AV	Single	87	64	23	1.6	31/8	37.5 (23.7)^a^	41.6 (29.1)^a^
Davis^121^	USA	NRCT, R	2005–2016	Rectal cancer	Multiple	31,427	12,335	19,092	ND	ND	1	1
Manchon‐Walsh^122^	Spain	NRCT, CCS, R	2011–2012	Rectal cancer	5	1359	842	517	13.2	524/310	60	60
Schnitzbauer^123^	Germany	NRCT, R	2007–2016	Rectal cancer	Multiple	16,378	4540	11,838	ND	1724/4819	56.4^a^	56.4^a^
Zhang XB^124^	China	NRCT, CCS, R	January 2010–September 2014	T4 rectal cancer	Single	125	86	39	2.3	54/23	48^b^	48^b^
Zhang ZZ^125^	China	NRCT, R	January 2008–December 2011	Rectal cancer	Single	228	112	116	ND	35/38	67.93 (21.89)^a^	67.97 (22.19)^a^
Zimmermann^126^	Germany	NRCT, CCS, R	January 2006–March 2016	Rectal cancer	2	496	248	248	0	109/94	63^b^	58^b^
Mar^127^	Spain	NRCT, P	June 2010–December 2012	Rectal cancer	Multiple	601	400	201	ND	201/113	24	24

Abbreviations: AV, anal verge; CCS, case–control study; DL, dentate line; nCRT, neoadjuvant chemoradiotherapy/chemoradiation; ND, no data available.

^a^ Mean (SD).

^b^ Median (range).

^c^ Median (IQR).

^d^ Mean (range).

**FIGURE 2:**
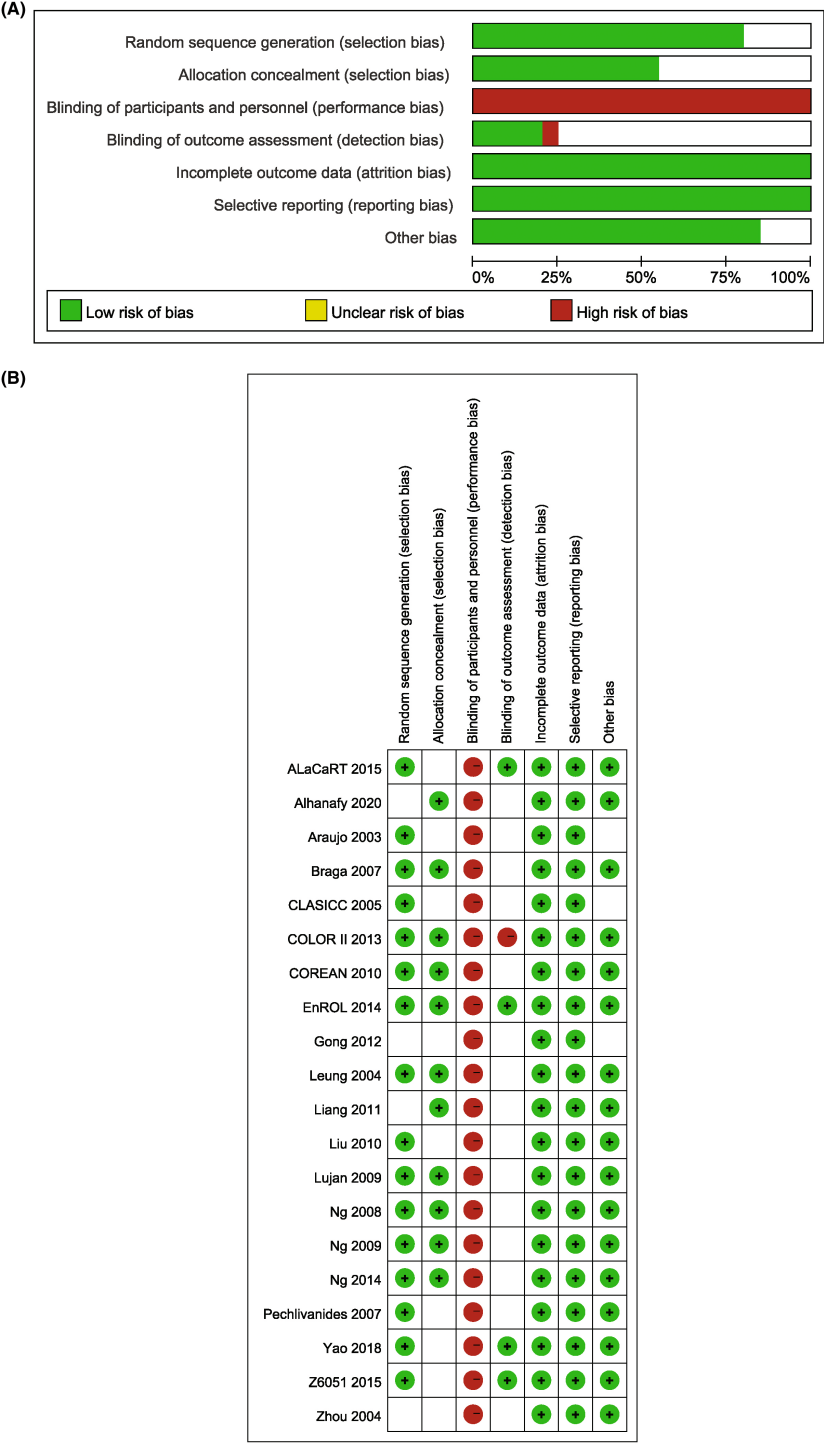
The risk of bias assessment of included RCT studies.

Quality assessment of the included articles according to the Cochrane Collaboration's tool for assessing risk of bias for RCTs and to the NOS for prospective NRCTs were shown in Table 2 and Figure 2.

**TABLE 2:** Quality assessment of the included non‐randomized controlled studies based on the Newcastle‐Ottawa Scale.

Reference	Selections				Comparability		Outcome assessment			Score
	1	2	3	4	5	6	7	8	9	
Schwandner, 1999^34^	*	*	*	*	‐	*	*	*	*	8
Leung^35^	*	*	*	*	‐	‐	*	*	*	7
Hartley^36^	*	*	*	*	‐	‐	*	*	*	7
Anthuber^37^	*	*	*	*	‐	‐	*	‐	*	6
Feliciotti^38^	*	*	*	*	*	‐	*	*	*	8
Wu^39^	*	*	*	*	*	*	*	‐	‐	7
Bretagnol^40^	*	*	*	*	‐	*	*	‐	‐	6
Breukink^41^	*	*	*	*	‐	‐	*	‐	*	6
Morino^42^	*	*	*	*	*	‐	*	*	*	8
Sahakitrungruang^43^	*	*	*	*	‐	*	*	‐	‐	6
Law^44^	*	*	*	*	*	‐	*	*	*	8
Wong^45^	*	*	*	*	*	*	*	*	*	9
Lelong^46^	*	*	*	*	*	‐	*	‐	‐	6
Staudacher^47^	*	*	*	*	*	*	*	*	*	9
Veenhof^48^	*	*	*	*	‐	*	*	*	*	8
Ströhlein^49^	*	*	*	*	‐	*	*	*	*	8
Ding^50^	*	*	*	*	*	‐	*	‐	*	7
González^51^	*	*	*	*	‐	‐	*	‐	*	6
Gouvas^52^	*	*	*	*	*	‐	*	‐	‐	6
Khaikin^53^	*	*	*	*	*	‐	*	‐	‐	6
Kim^54^	*	*	*	*	‐	‐	*	*	*	7
Koulas^55^	*	*	*	*	‐	*	*	‐	‐	6
Laurent^56^	*	*	*	*	‐	‐	*	*	*	7
Law^57^	*	*	*	*	*	‐	*	*	*	8
Biondo^58^	*	*	*	*	*	*	*	‐	‐	7
Denoya^59^	*	*	*	*	‐	*	*	‐	‐	6
Feng^60^	*	*	*	*	‐	*	*	*	*	8
Fujimoto^61^	*	*	*	*	‐	*	*	‐	*	7
Baik^62^	*	*	*	*	*	*	*	*	*	9
da Luz Moreira^63^	*	*	*	*	*	*	*	‐	‐	7
Greenblatt^64^	*	*	*	*	*	‐	*	‐	‐	6
June^65^	*	*	*	*	*	‐	*	‐	‐	6
Li^66^	*	*	*	*	*	*	*	*	*	9
Park^67^	*	*	*	*	*	*	*	*	*	9
Yamamoto^68^	*	*	*	*	*	*	*	‐	‐	7
Gunka^69^	*	*	*	*	*	*	*	*	*	9
Jefferies^70^	*	*	*	*	*	‐	*	*	*	8
Kellokumpu^71^	*	*	*	*	*	*	*	*	*	9
Laurent^72^	*	*	*	*	*	*	*	*	*	9
Li^73^	*	*	*	*	*	‐	*	*	*	8
Mckay^74^	*	*	*	*	*	‐	*	‐	‐	6
Seshadri^75^, ^76^	*	*	*	*	*	*	*	*	*	9
Siani^77^	*	*	*	*	‐	*	*	*	*	8
Kuo^78^	*	*	*	*	‐	*	*	‐	‐	6
Lee^79^	*	*	*	*	*	*	*	*	*	9
Lujan^80^	*	*	*	*	‐	‐	*	‐	*	6
Park^81^	*	*	*	*	‐	‐	*	*	*	7
Penninckx^82^	*	*	*	*	‐	‐	*	*	*	7
Yang^83^	*	*	*	*	*	‐	*	‐	‐	6
Ferko^84^	*	*	*	*	*	‐	*	‐	‐	6
Inada^85^	*	*	*	*	*	‐	*	‐	‐	6
Inomata^86^	*	*	*	*	*	‐	*	‐	‐	6
Keller^87^	*	*	*	*	‐	‐	*	*	*	7
Kusano^88^	*	*	*	*	‐	‐	*	*	*	7
Wang^89^	*	*	*	*	*	*	*	*	*	9
Zhou^90^	*	*	*	*	*	*	*	*	*	9
Chi^91^	*	*	*	*	*	*	*	*	*	9
Guo^92^	*	*	*	*	*	‐	*	*	*	8
Huang^93^	*	*	*	*	*	‐	*	*	*	8
Kim^94^	*	*	*	*	‐	‐	*	*	*	7
Li^95^	*	*	*	*	*	*	*	*	*	9
Nussbaum^96^	*	*	*	*	*	‐	*	‐	‐	6
Wang^97^	*	*	*	*	*	*	*	‐	*	8
Zeng^98^	*	*	*	*	*	*	*	*	*	9
Zhou^99^	*	*	*	*	‐	‐	*	*	*	7
Allaix^100^	*	*	*	*	‐	*	*	*	*	8
Chen ZX^101^	*	*	*	*	*	‐	*	‐	‐	6
Chen WP^102^	*	*	*	*	‐	*	*	‐	‐	6
Keskin^103^	*	*	*	*	*	‐	*	*	*	8
Kim^104^	*	*	*	*	*	‐	*	*	*	8
Liu^105^	*	*	*	*	‐	*	*	‐	‐	6
Nonaka^106^	*	*	*	*	‐	*	*	*	*	8
Odermatt^107^	*	*	*	*	*	*	*	*	*	9
De'Angelis^108^	*	*	*	*	*	*	*	*	*	9
Matsuhashi^109^	*	*	*	*	*	‐	*	‐	‐	6
Silva‐Velazco^110^	*	*	*	*	*	‐	*	‐	‐	6
Wu^111^	*	*	*	*	*	*	*	*	*	9
Andersen^112^	*	*	*	*	*	‐	*	‐	‐	6
Aydın^113^	*	*	*	*	‐	‐	*	*	*	7
Chen^114^	*	*	*	*	*	‐	*	‐	‐	6
Draeger^115^	*	*	*	*	‐	‐	*	*	*	7
Garfinkle^116^	*	*	*	*	*	‐	*	‐	‐	6
Hida^117^	*	*	*	*	‐	‐	*	*	*	7
Tayar^118^	*	*	*	*	*	‐	*	‐	‐	6
Wu^119^	*	*	*	*	*	‐	*	*	*	8
Chen^120^	*	*	*	*	‐	‐	*	*	*	7
Davis^121^	*	*	*	*	*	‐	*	‐	‐	6
Manchon‐Walsh^122^	*	*	*	*	*	‐	*	*	*	8
Schnitzbauer^123^	*	*	*	*	‐	‐	*	*	*	7
Zhang XB^124^	*	*	*	*	*	‐	*	*	*	8
Zhang ZZ^125^	*	*	*	*	*	‐	*	*	‐	7
Zimmermann^126^	*	*	*	*	*	‐	*	*	*	8
Mar^127^	*	‐	*	*	*	‐	*	*	*	7

*Note*: Selections: 1, Representativeness of laparoscopic group (if yes, one point). 2, The open surgery group was drawn from the same medical center (if yes, one point). 3, Secure record of ascertainment of exposure (surgical records, one point). 4, Demonstration that outcome of interest was not present at start of study (if yes, one point). Comparability: 5, Group comparable for age, gender, and American Society of Anesthesiologists classification of physical status or Charlson's Comorbidity Index (if yes, one points; no point if one of these characteristics was not reported or if the two groups differed). 6, Group comparable for neoadjuvant chemoradiotherapy, tumor location, stage, and surgical procedure (if yes, one points; no point if one of these characteristics was not reported or if the two groups differed). Outcome assessment: 7, Assessment of outcome (if independent blind assessment or record linkage, one point). 8, Was follow‐up long enough for outcomes to occur (if yes, one point). 9, Adequacy of follow up of cohorts (if yes, one point; no points if follow‐up not reported).

### 3.3 Meta‐analysis results

The specific data of the results of the meta‐analysis comparing laparoscopic versus open surgery for rectal cancer in the aspects of intraoperative, pathological, postoperative and survival outcomes were listed in Table 3.

**TABLE 3:** Results of the meta‐analysis comparing laparoscopic versus open surgery for rectal cancer.

Outcome variables	Studies (*n*) (RCT/NRCT)	Patients (*n*) (LAP/OP)	Analysis model	Total			RCT subgroup			NRCT subgroup		
				MD/OR [95%CI]	*I* ^2^ (%)	*p*‐Value	MD/OR [95%CI]	*I* ^2^ (%)	*p*‐Value	MD/OR [95%CI]	*I* ^2^ (%)	*p*‐Value
Intraoperative outcomes												
Operation time	82 (17/65)	24,106/37127	M	25.67 [21.02, 30.32]	97	<0.0001	36.31 [26.49, 46.14]	97	<0.0001	22.45 [17.10, 27.79]	97	<0.0001
Blood loss	66 (16/50)	7604/797	M	−131.42 [−153.61, −109.23]	99	<0.0001	−110.85 [−147.23, −74.47]	99	<0.0001	−138.53 [−166.66, −110.40]	100	<0.0001
Incision length	12 (7/5)	1945/2244	M	−12.82 [−12.85, −12.79]	100	<0.0001	−11.77 [−11.87, −11.68]	100	<0.0001	−12.94 [−12.97, −12.91]	99	<0.0001
Pathological outcomes												
Harvested lymph nodes	84 (16/68)	18,459/22609	M	−0.44 [−0.78, 0.10]	95	0.01	0.05 [−0.59, 0.69]	87	0.87	−0.60 [−1.01, −0.19]	96	0.004
CMR (+)	43 (10/33)	14,126/16844	F	0.79 [0.72, 0.85]	40	<0.0001	1.21 [0.91, 1.59]	0	0.19	0.76 [0.69, 0.82]	41	<0.0001
DMR (+)	18 (2/16)	9919/10211	F	0.75 [0.66, 0.85]	0	<0.0001	1.13 [0.35, 3.66]	0	0.84	0.74 [0.65, 0.85]	6	<0.0001
TME completeness	20 (6/14)	4891/7295	M	1.02 [0.99, 1.05]	60	0.18	0.96 [0.91, 1.01]	17	0.10	1.04 [1.01, 1.07]	58	0.02
Postoperative outcomes												
First bowel movement	49 (15/34)	6593/6878	M	−0.89 [−1.06, −0.73]	98	<0.0001	−0.83 [−1.06, −0.61]	98	<0.0001	−0.90 [−1.08, −0.73]	96	<0.0001
Postoperative analgesic need	11 (5/6)	996/1029	M	−0.88 [−1.38, −0.38]	97	0.0006	−0.60 [−1.21, 0.02]	96	0.06	−1.11 [−2.28, 0.06]	97	0.06
Days of ambulation	12 (6/6)	1415/1471	M	−0.89 [−1.09, −0.69]	72	<0.0001	−0.69 [−0.87, −0.51]	21	<0.0001	−1.03 [−1.34, −0.71]	81	<0.0001
Length of hospital stay	92 (16/76)	38,446/149664	M	−0.62 [−0.77, −0.47]	99	<0.0001	−0.63 [−0.94, −0.31]	96	<0.0001	−0.62 [−0.78, −0.45]	99	<0.0001
Postoperative complications	86 (16/70)	19,412/124070	F	0.83 [0.81, 0.85]	7	<0.0001	0.92 [0.84, 1.00]	14	0.06	0.82 [0.80, 0.84]	0	<0.0001
Hemorrhage	47 (9/38)	13,221/116441	F	0.74 [0.62, 0.89]	0	0.001	0.68 [0.40, 1.17]	3	0.16	0.75 [0.62, 0.91]	0	0.003
Wound infection	75 (14/61)	26,866/39726	F	0.50 [0.47, 0.54]	13	<0.00001	0.59 [0.46, 0.77]	28	0.0001	0.50 [0.47, 0.53]	7	<0.00001
Ileus	70 (15/55)	15,826/115502	F	0.92 [0.87, 0.96]	0	0.0008	0.71 [0.53, 0.94]	0	0.02	0.92 [0.88, 0.97]	0	0.003
Anastomotic leak	76 (16/60)	13,376/16968	F	1.01 [0.93, 1.10]	0	0.77	0.95 [0.73, 1.23]	0	0.68	1.02 [0.93, 1.12]	0	0.65
Abscess	46 (9/37)	20,199/32131	F	0.94 [0.87, 1.02]	0	0.12	1.10 [0.74, 1.63]	0	0.64	0.94 [0.87, 1.01]	0	0.09
Reintervention within 30 days	36 (9/27)	7733/13167	F	0.96 [0.86, 1.07]	0	0.44	1.01 [0.80, 1.27]	0	0.96	0.94 [0.83, 1.07]	0	0.37
Mortality within 30 days	48 (8/40)	34,690/145359	F	0.66 [0.59, 0.75]	0	<0.0001	0.79 [0.44, 1.43]	0	0.44	0.66 [0.58, 0.75]	0	<0.0001
Survival outcomes												
3‐year LR	33 (7/26)	5511/5828	F	0.81 [0.69, 0.95]	0	0.008	1.04 [0.74, 1.46]	0	0.83	0.76 [0.64, 0.90]	0	0.002
3‐year DR	21 (5/16)	3323/3671	F	0.91 [0.80, 1.02]	0	0.11	1.01 [0.82, 1.26]	0	0.90	0.86 [0.75, 1.00]	9	0.05
3‐year DFS	26 (6/20)	3947/4228	F	1.03 [1.01, 1.06]	22	0.01	1.04 [0.99, 1.09]	0	0.17	1.03 [1.00, 1.06]	32	0.03
3‐year OS	32 (7/25)	7022/7989	F	1.03 [1.01, 1.05]	35	0.007	1.01 [0.98, 1.05]	10	0.54	1.03 [1.01,1.06]	40	0.006
5‐year LR	23 (4/19)	3553/4131	F	0.66 [0.57, 0.75]	41	<0.0001	0.99 [0.53, 1.83]	0	0.97	0.64 [0.55,0.74]	44	<0.0001
5‐year DR	19 (5/14)	2444/2175	F	0.89 [0.78, 1.01]	8	0.07	0.87 [0.68, 1.11]	24	0.26	0.89 [0.77, 1.04]	8	0.16
5‐year DFS	22 (5/17)	7893/15078	M	1.06 [1.02, 1.10]	54	0.002	1.02 [0.95, 1.09]	0	0.66	1.07 [1.02, 1.11]	62	0.002
5‐year OS	29 (5/24)	10,069/17046	M	1.05 [1.02, 1.09]	69	0.001	1.01 [0.93, 1.10]	11	0.75	1.06 [1.02, 1.10]	73	0.001

### 3.4 Intraoperative outcomes

Operation time was reported in 17 RCTs and 65 NRCTs. The weighted mean operation time was significantly increased in the laparoscopic group than open surgery group (248.37 vs. 221.96 min, MD = 25.67, 95% CI: 21.02–30.32, *I* ^2^ = 97%, *p* < 0.0001) (Figure S1).

Blood loss was reported in 16 RCTs and 50 NRCTs. Significant less blood loss was found in laparoscopic surgery compared with open surgery (151.30 vs. 276.60 mL, MD = −131.42, 95% CI: −153.61 to −109.23, *I* ^2^ = 99%, *p* < 0.0001) (Figure S2).

Incision length was reported in 7 RCTs and 5 NRCTs. Significant shorter surgical incision was found in laparoscopic surgery compared with open surgery (5.36 vs. 17.05 cm, MD = −12.82, 95% CI: −12.85 to −12.79, *I* ^2^ = 100%, *p* < 0.0001) (Figure S3).

### 3.5 Pathological outcomes

The number of harvested lymph nodes were reported in 16 RCTs and 68 NRCTs. The weighted mean lymph node retrieved in laparoscopic surgery was significant less than open surgery (14.67 vs. 14.86, MD = −0.44, 95% CI: −0.78 to −0.10, *I* ^2^ = 95%, *p* = 0.01). However, the subgroup meta‐analysis showed no significant difference was found in RCT subgroup (MD = 0.05, 95% CI: −0.59 to 0.69, *I* ^2^ = 87%, *p* = 0.87) (Figure S4).

CMR positive rate was reported in 10 RCTs and 33 NRCTs. The weighted mean CMR positive rate was significantly reduced in laparoscopic surgery compared with open surgery (6.24% vs. 8.88%, RR = 0.79, 95% CI: 0.72 to 0.85, *I* ^2^ = 40%, *p* < 0.0001), but there was no significant difference in RCT subgroup (RR = 1.21, 95% CI: 0.91–1.59, *I* ^2^ = 0%, *p* = 0.19) (Figure S5).

DMR positive rate was reported in 2 RCTs and 16 NRCTs. The weighted mean DMR positive rate was significantly reduced in laparoscopic surgery compared with open surgery (3.84% vs. 4.99%, RR = 0.75, 95% CI: 0.66–0.85, *I* ^2^ = 0%, *p* < 0.0001), but there was no significant difference in RCT subgroup (RR = 1.13, 95% CI: 0.35–3.66, *I* ^2^ = 0%, *p* = 0.84) (Figure S6).

The macroscopic quality of total mesorectal excision (TME) specimen was reported in 6 RCTs and 14 NRCTs. No significant difference of TME completeness was found in laparoscopic surgery compared with open surgery (79.41% vs. 76.07%, RR = 1.02, 95% CI: 0.99–1.05, *I* ^2^ = 60%, *p* = 0.18) (Figure S7).

### 3.6 Postoperative outcomes

Bowel movement recovery was reported in 15 RCTs and 34 NRCTs. The weighted mean time before the first bowel movement or passing the first flatus was significantly shorter in laparoscopic surgery compared with open surgery (2.66 vs. 3.46 days, MD = −0.89, 95% CI: −1.06 to −0.73, *I* ^2^ = 98%, *p* < 0.0001) (Figure S8).

The days of postoperative analgesic need were reported in 5 RCTs and 6 NRCTs. The weighted mean time of postoperative analgesic need was significantly decreased in laparoscopic surgery compared with open surgery (3.063 vs. 3.61 days, MD = −0.88, 95% CI: −1.38 to −0.38, *I* ^2^ = 97%, *p* = 0.0006) (Figure S9).

The days of ambulation were reported in 6 RCTs and 6 NRCTs. Significant less days of ambulation was found in laparoscopic surgery compared with open surgery (3.28 vs. 4.49 days, MD = −0.89, 95% CI: −1.09 to −0.69, *I* ^2^ = 72%, *p* < 0.0001) (Figure S10).

The length of hospital stay was reported in 16 RCTs and 76 NRCTs. The weighted mean time of hospital stay was significantly shorter in laparoscopic surgery compared with open surgery (7.33 vs.7.44 days, MD = −0.62, 95% CI: −0.77 to −0.47, *I* ^2^ = 99%, *p* < 0.0001) (Figure S11).

Postoperative complications were reported in 16 RCTs and 70 NRCTs. The weighted mean incidence of overall postoperative complications was significantly decreased in laparoscopic surgery compared with open surgery (28.27% vs. 35.27%, RR = 0.83, 95% CI: 0.81–0.85, *I* ^2^ = 7%, *p* < 0.0001), but no significant difference was found in the RCT subgroup (RR = 0.92, 95% CI: 0.84–1.00, *I* ^2^ = 14%, *p* = 0.06) (Figure S12). Then we focused on the comparison of five major postoperative complications. The total meta‐analysis revealed significant low rate of hemorrhage (1.25% vs. 5.02%, RR = 0.74, 95% CI: 0.62–0.89, *I* ^2^ = 0%, *p* = 0.001) (Figure S13), wound infection (4.84% vs. 10.02%, RR = 0.50, 95% CI: 0.47–0.54, *I* ^2^ = 13%, *p* < 0.00001) (Figure S14) and ileus (9.19% vs. 16.81%, RR = 0.92, 95% CI: 0.87–0.96, *I* ^2^ = 0%, *p* = 0.0008) (Figure S15) in laparoscopic surgery compared with open surgery. No significant difference of incidence rate of anastomotic leak (7.19% vs. 6.70%, RR = 1.01, 95% CI: 0.93–1.10, *I* ^2^ = 0%, *p* = 0.77) (Figure S16) and intra‐abdominal, pelvic or retroperitoneal abscess after surgery (5.03% vs. 5.52%, RR = 0.94, 95% CI: 0.87–1.02, *I* ^2^ = 0%, *p* = 0.12) (Figure S17) were found between the two groups.

Reintervention within 30 days after surgery was reported in 9 RCTs and 27 NRCTs. No significant difference of reintervention rate was found in laparoscopic surgery compared with open surgery (7.02% vs. 5.54%, RR = 0.96, 95% CI: 0.86–1.07, *I* ^2^ = 0%, *p* = 0.44) (Figure S18).

Mortality within 30 days was reported in 8 RCTs and 40 NRCTs. Significant decreased mortality was significantly decreased in laparoscopic surgery compared with open surgery (0.98% vs. 1.29%, RR = 0.66, 95% CI: 0.59–0.75, *I* ^2^ = 0%, *p* < 0.0001), but no significant difference was found in the RCT subgroup (RR = 0.79, 95% CI: 0.44–1.43, *I* ^2^ = 0%, *p* = 0.44) (Figure S19).

### 3.7 Survival outcomes

Thirty‐three studies reported 3‐year local recurrence (LR) and 23 studies reported 5‐year LR. The laparoscopic surgery group showed significantly decreased 3‐year LR (5.12% vs. 6.01%, RR = 0.81, 95% CI: 0.69–0.95, *I* ^2^ = 0%, *p* = 0.008) (Figure S20) and 5‐year LR (7.46% vs. 13.73%, RR = 0.66, 95% CI: 0.57–0.75, *I* ^2^ = 41%, *p* < 0.0001) (Figure S21) compared with open surgery group. However, there were no significant differences in the RCT subgroup.

Twenty‐one studies reported 3‐year distant recurrence (DR) and 19 studies reported 5‐year DR. There were no significant differences of 3‐year DR (13.36% vs. 14.79%, RR = 0.91, 95% CI: 0.80–1.02, *I* ^2^ = 0%, *p* = 0.11) (Figure S22) or 5‐year DR (16.24% vs. 17.01%, RR = 0.89, 95% CI: 0.78–1.01, *I* ^2^ = 8%, *p* = 0.07) (Figure S23) between the two groups.

Twenty‐six studies reported 3‐year disease free survival (DFS) and 22 studies reported 5‐year DFS. The laparoscopic surgery group showed significantly increased 3‐year DFS (73.57% vs. 74.55%, RR = 1.03, 95% CI: 1.01–1.06, *I* ^2^ = 22%, *p* = 0.01) (Figure S24) and 5‐year DFS (78.41% vs. 73.28%, RR = 1.06, 95% CI: 1.02–1.10, *I* ^2^ = 54%, *p* = 0.002) (Figure S25) compared with open surgery group. However, there were no significant differences in the RCT subgroup.

Thirty‐two studies reported 3‐year overall survival (OS) and 29 studies reported 5‐year OS. The laparoscopic surgery group showed significantly increased 3‐year OS (84.88% vs. 82.78%, RR = 1.03, 95% CI: 1.01–1.05, *I* ^2^ = 35%, *p* = 0.007) (Figure S26) and 5‐year OS (79.09% vs. 75.52%, RR = 1.05, 95% CI: 1.02–1.09, *I* ^2^ = 69%, *p* = 0.001) (Figure S27) compared with open surgery group. However, there were no significant differences in the RCT subgroup.

Only three studies reported 10‐year LR and DR, four studies reported 10‐year DFS and OS. No significant differences were showed based on these 10‐year data of the two groups (data not shown).

## 4 DISCUSSION

Since laparoscopic surgery were applied to the resection of rectal cancer, numerous studies had been carried out to evaluate the surgical and oncological outcomes of this approach compared with traditional open surgery. Meanwhile, countless meta‐analyses had drawn many conclusions based on the analysis of different types of trials. However, there had been no comprehensive assessment and comparison covering all the aspects of the two procedures in the treatment of rectal cancer, especially the pathological outcomes and long‐term survival. We started this largest‐to‐date meta‐analysis including 113 studies spanning over 30 years to fill the vacuum. In order to include as many patients as possible, not only RCTs but also numerous NRCTs from different medical centers were incorporated into our research, and conditions of inclusion criteria (different locations or stages of rectal cancer, with or without nCRT, different operation styles and studies with small sample size) was relaxed to a certain extent. The 93 NRCTs showed low risk of bias which left these reports convincing. Based on the overall investigation, the genuine effectiveness of laparoscopic proctectomy could be assessed accurately.

The surgical approach for upper rectal cancer was equivalent to the sigmoid cancer, while the standard treatment for mid and low rectal cancer was TME. A successful resection of rectal cancer should meet the conditions of complete TME, a clear circumferential margin (CRM, ≥1 mm) and a clear distal resection margin (DRM, ≥1 mm).^29^ The standardization of TME improved the prognosis of rectal cancer by reducing the positive margin and local recurrence rates.^128^ This concept demands the sharp separation of the visceral fascia from the parietal plane. The resected specimen was the whole rectal tumor with an intact coverage including the main lymphatic drainage.^129^ It was evident that the magnified, illuminated images and pneumoperitoneum provided by laparoscopy facilitated the operation in the narrow pelvic space. In that case, a high‐quality of resected specimen might be more easily to obtain. The results of this meta‐analysis indicated that the 3‐year LR and 5‐year LR were both significantly decreased after the laparoscopic surgery, and eventually lead to longer DFS and OS. Part of that improvement might be attributable to the radical resection of the tumor and mesorectum under direct vision created by laparoscopy. Although there was no significant difference of TME completeness and number of harvested lymph nodes found in laparoscopic surgery compared with open surgery, the positive rate of CMR and DMR were both reduced in the laparoscopy group. These results might contribute to the decreased LR. NCCN guidelines mentioned that some studies had shown that laparoscopy is associated with higher rates of CMR and incomplete TME, which was not supported by our large‐scale meta‐analysis. Moreover, there were no significant differences of 3‐year DR and 5‐year DR between the two groups. It suggested that laparoscopy might reduce the local recurrence without promoting distant metastasis. It should be noted that no significant difference of these survival indexes was found in the RCT subgroup.

The ultimate goal of treatment for cancer was not only an optimal oncological outcome, but also rapid recovery and improved quality of life. Our study revealed that patients could benefit from laparoscopic surgery in the postoperative recovery, including earlier return of bowel function, improved cosmesis and reduced pain owing to small incisions, earlier ambulation, shorter hospital stay and less complications. The total incidence of postoperative complications, morbidity of major complications and mortality within 30 days were all significantly decreased in the laparoscopy group without increased risk of reintervention. The fast recovery after surgery enabled patients to initiate following adjuvant therapy in a shorter period, which was a favorable factor contributing to the improvement of the prognosis.

The pneumoperitoneum created by laparoscopic surgery effectively expanded the activity of abdomen. However, the expansion of pelvic cavity was limited. Accomplishment of anastomosis under laparoscopy without collateral damage required precise manipulation of the surgeons. Only the well‐trained doctors were qualified to perform laparoscopic rectal resection which could provide the equivalent oncologic outcomes and faster postoperative recovery. The COREAN trial was competed by seven highly skilled laparoscopic specialists, it suggested that better short‐term outcome could be achieved in expert hands.^20^ Several researches recommended effective self‐taught learning curve was about 50–80 laparoscopic rectal resections.^130^ The rates of conversion to laparotomy, operation time, blood loss and complications were all decreased in the cases performed by senior surgeons. Moreover, the natural orifice specimen extraction surgery and robotic‐assisted laparoscopic surgery have been applied in the surgical treatment of cancer as technology evolved. These kinds of minimal invasive surgery demanding special surgical skills could further decrease the operative trauma and promote postoperative recovery. In that case, the role of surgeons in the surgery and the outcomes of patients was crucial. The experience of surgeons should be taken into consideration in the further strictly controlled trials. The laparoscopic surgery did require longer operative time, but time gap between the two approaches could be closing as the well‐trained surgeons have perfected their surgical techniques.

Drawbacks of laparoscopic protectomy will appear when it comes to specific cases, such as super obese patients or advanced tumor with extensive invasion. The difficulty of laparoscopy will lead to the conversion into open surgery. The range of conversion rate reported in the literatures mentioned in this paper was from 0% to 23.2%, and the average rate was 7.73%. The reasons for conversion might be as follows: too large tumors, low‐located T4 tumors, previously irradiated pelvis, collateral damages of surgical operation and so on. The oncological outcomes of cases converted to open surgery is a controversial issue. Some studies showed similar outcomes, but others revealed worsen short‐term outcomes,^131^ higher rates of LR and reduced cumulative DFS.^132^ Most conversion in laparoscopic surgery are actually avoidable with adequate preoperative evaluation based on diagnostic imaging tests and a well‐trained laparoscopic surgical team. The extended laparoscopic instruments also facilitate the operation on the obese patients.

T4 tumor is always the focus of controversy. It was identified as those that invade into other organs and structures and/or perforate the visceral peritoneum. All the guidelines suggest T4 rectal cancer as a contraindication for laparoscopic surgery, because the locally advanced tumor have a high risk of CMR or need *en bloc* resection of the adjacent infiltrated organs which might increase the convention rates and collateral increasing morbidity. Some studies have assessed the pathologic and oncologic results and suggested that laparoscopic surgery was feasible for T4 colorectal cancer.^108^, ^124^ In this study, laparoscopic resection for all the stages of rectal cancer were merged and generally compared with open surgery. In that case, the analysis to some foci of controversy was ignored, such as T4 tumor, resection of primary tumor following nCRT and so on. Further stratified and grouped comparisons were urgently needed to compare the effect of these two surgical approaches in the specific population of rectal patients. Moreover, there were many more aspects in the recovery and prognosis of patients received surgical management deserves to be focused on and compared between the laparoscopy and open surgery, including the gender differences based on different anatomical structure, urinary and sexual dysfunction, incisional hernia, hospitalized cost and postoperative immunological changes. The robot‐assisted laparoscopy has become emerging trend in the treatment of rectal cancer. The surgical outcomes of robotic surgery should also be compared with traditional laparoscopy and open surgery separately.

This article incorporated a large number of NRCTs which lead to a certain degree of bias in case selection and comparability. But they had already been accessed as high quality. Moreover, some NRCTs might have a preference in selection of population for specific purpose, such as elderly patients, T4 tumors, patients received nCRT and so on. These results were all unique and meaningful in the interpretation of the effect of laparoscopic proctectomy. As it should be, more large‐scale RCTs and observational studies with stratified method, multivariable risk adjustment or propensity score analysis were urgently needed to evaluate the surgical outcomes of laparoscopy.

## 5 CONCLUSIONS

Based on the meta‐analysis of both RCTs and NRCTs, laparoscopic proctectomy was non‐inferior to open surgery in terms of pathological results, postoperative recovery and long‐term survival in the treatment of rectal cancer. It should be recommended as the priority option for the eligible patients in the surgical treatment of rectal cancer.

## AUTHOR CONTRIBUTIONS

**Ling Ma:** Conceptualization (equal); data curation (equal); formal analysis (equal); funding acquisition (equal); investigation (equal); methodology (equal); project administration (equal); resources (equal); software (equal); supervision (equal); validation (equal); visualization (equal); writing – original draft (equal); writing – review and editing (equal). **Hai‐jiao Yu:** Data curation (equal); writing – original draft (equal); writing – review and editing (equal). **Yu‐bing Zhu:** Data curation (equal); writing – original draft (equal); writing – review and editing (equal). **Wen‐xia Li:** Formal analysis (equal); software (equal); writing – original draft (equal); writing – review and editing (equal). **Kai‐yu Xu:** Formal analysis (equal). **Ai‐min Zhao:** Project administration (equal); resources (equal). **Lei Ding:** Conceptualization (equal); methodology (equal); supervision (equal); validation (equal); visualization (equal); writing – review and editing (equal). **Hong Gao:** Conceptualization (equal); methodology (equal); supervision (equal); validation (equal); visualization (equal); writing – review and editing (equal).

## FUNDING INFORMATION

None.

## CONFLICT OF INTEREST STATEMENT

All authors declare no conflicts of interest.

## PRECIS

Laparoscopy is non‐inferior to open surgery for rectal cancer in intraoperative, pathological, postoperative and long‐term outcomes. Laparoscopic surgery provides short‐term advantages, including faster recovery and less complications.

## Supporting information


Figure S1.

Data S1.

## Data Availability

The original contributions presented in the study are included in the article/supplementary material; further inquiries can be directed to the corresponding authors.
