# Supplementary material for: Laparoscopy is non‐inferior to open surgery for rectal cancer: A systematic review and meta‐analysis
Source: Cancer Med. 2024 Jul 5;13(13):e7363. doi: 10.1002/cam4.7363 (PMC11226727; doi:10.1002/cam4.7363)

FIGURE S1 Forest plot for operation time

Study or Subgroup	Laparoscopic surgery Mean	SD	Total	Open surgery Mean	SD	Total	Weight	Mean Difference IV, Random, 95% CI	Year
**RCT**									
Leung et al, 2004	189.9	55.4	203	144.2	57.8	200	1.5%	45.70 [34.64, 56.76]	2003
Zhou et al, 2004	120	18.333	82	106	25	89	1.6%	14.00 [7.46, 20.54]	2004
Braga et al, 2007	262	72	83	209	70	85	1.2%	53.00 [31.52, 74.48]	2007
Ng et al, 2008	213.5	46.2	51	163.7	43.4	48	1.3%	49.80 [32.15, 67.45]	2008
Lujan et al, 2009	193.7	45.1	101	172.9	59.4	103	1.4%	20.80 [6.34, 35.26]	2009
Ng et al, 2009	213.1	59.3	76	154	70.3	77	1.2%	59.10 [38.50, 79.70]	2009
Liu et al, 2010	161	35	98	140	20	88	1.5%	21.00 [12.91, 29.09]	2010
COREAN, 2010	244.9	75.4	170	197	62.9	170	1.4%	47.90 [33.14, 62.66]	2010
Liang et al, 2011	138.08	23.79	169	118.53	21.989	174	1.6%	19.55 [14.70, 24.40]	2011
Gong et al, 2012	216.4	68.3	67	162.7	42.5	71	1.3%	53.70 [34.59, 72.81]	2012
COLOR II, 2013	240	19.333	699	188	15	345	1.6%	52.00 [49.86, 54.14]	2013
EnROL, 2014	220	67	29	186	48	27	1.0%	34.00 [3.63, 64.37]	2014
Ng et al, 2014	211.6	53	40	153	41.1	40	1.2%	58.60 [37.82, 79.38]	2014
ALaCaRT, 2015	210	15	238	190	13.333	235	1.6%	20.00 [17.44, 22.56]	2015
Z6051, 2015	266.2	101.9	240	220.6	92.4	222	1.3%	45.60 [27.88, 63.32]	2015
Yao et al, 2018	160.5	46.1	60	132.6	38.4	60	1.4%	27.90 [12.72, 43.08]	2018
Alhanafy et al, 2020	211.5	31.8	60	200	30.2	60	1.5%	11.50 [0.40, 22.60]	2020
**Subtotal (95% CI)**			**2466**			**2094**	**23.4%**	**36.31 [26.49, 46.14]**	
Heterogeneity: Tau² = 368.59; Chi² = 514.44, df = 16 (P < 0.00001); I² = 97%									
Test for overall effect: Z = 7.24 (P < 0.00001)									
**NRCT**									
Schwandner et al, 1999	281	80	32	209	66	32	0.8%	72.00 [36.07, 107.93]	1999
Leung et al, 2000	215.6	47.9	25	166.3	36.3	34	1.2%	49.30 [26.91, 71.69]	2000
Hartley et al, 2001	189.3414	39.7517	22	124.2823	31.6995	21	1.2%	65.06 [43.62, 86.50]	2001
Anthuber et al, 2003	217.9	70.9	101	218.9	74	334	1.4%	-1.00 [-16.94, 14.94]	2003
Wu et al, 2004	189	18	18	146	22	18	1.4%	43.00 [29.87, 56.13]	2004
Sahakitrungruang et al, 2005	372.5	82.5	24	244.75	71.25	25	0.7%	127.75 [84.51, 170.99]	2005
Breukink et al, 2005	200	53.75	41	180	37.5	41	1.2%	20.00 [-0.06, 40.06]	2005
Law et al, 2006	200	43.33	98	127	50	167	1.5%	73.00 [61.55, 84.45]	2006
Wong et al, 2006	145	33.333	71	156	87.5	31	0.9%	-11.00 [-42.76, 20.76]	2006
Veenhof et al, 2007	250	18.75	50	197.5	13.75	50	1.6%	52.50 [46.06, 58.94]	2007
Staudacher et al, 2007	251	116	108	218	91	79	1.0%	33.00 [3.31, 62.69]	2007
Lelong et al, 2007	432	109	104	278	98	68	0.9%	154.00 [122.67, 185.33]	2007
González et al, 2009	181.3	23.5	28	206.1	33.5	28	1.4%	-24.80 [-39.96, -9.64]	2009
Khaikin et al, 2009	240	75	32	185	76.25	50	0.9%	55.00 [21.50, 88.50]	2009
Gouvas et al, 2009	166	26	45	143	34	43	1.4%	23.00 [10.31, 35.69]	2009
Ding et al, 2009	180	81	115	150	94.5	220	1.3%	30.00 [10.63, 49.37]	2009
Law et al, 2009	200	11.667	111	150	10	310	1.6%	50.00 [47.56, 52.44]	2009
Denoya et al, 2010	267	76	32	205	49	32	0.9%	62.00 [30.67, 93.33]	2010
Fujimoto et al, 2010	293	65.25	35	466.25	173.75	19	0.3%	-173.25 [-254.31, -92.19]	2010
Jun et al, 2011	158.1	49.2	123	233.8	59.2	88	1.4%	-75.70 [-90.82, -60.58]	2011
Yamamoto et al, 2011	393.75	48.75	22	318.75	68.25	22	0.8%	75.00 [39.95, 110.05]	2011
Park et al, 2011	281.8	94.8	130	297.8	86.4	80	1.1%	-16.00 [-40.98, 8.98]	2011
Baik et al, 2011	237.5	13.4	54	244	77.5	108	1.4%	-6.50 [-21.55, 8.55]	2011
Li et al, 2011	210	53.333	113	180	65	123	1.4%	30.00 [14.88, 45.12]	2011
Greenblatt et al, 2011	242	96	1040	219	99	4380	1.6%	23.00 [16.47, 29.53]	2011
Kellokumpu et al, 2012	165	6.667	100	155	11.667	91	1.6%	10.00 [7.27, 12.73]	2012
Siani et al, 2012	215	43	30	195	29	30	1.3%	20.00 [1.44, 38.56]	2012
Laurent et al, 2012	390	85	110	300	65	65	1.2%	90.00 [67.59, 112.41]	2012
Seshadri et al, 2012	270	40	72	240	35	72	1.4%	30.00 [17.72, 42.28]	2012
Mckay et al, 2012	226.8	72.8	157	201	57.9	388	1.4%	25.80 [13.04, 38.56]	2012
Li et al, 2012	176.4	28.3	381	144.2	20.6	276	1.6%	32.20 [28.46, 35.94]	2012
Kuo et al, 2013	374.3	90	28	416.5	100	30	0.6%	-42.20 [-91.11, 6.71]	2013
Yang et al, 2013	160	40	87	150	35	90	1.5%	10.00 [-1.09, 21.09]	2013
Park et al, 2013	215.28	76.2	406	261.5	81.1	406	1.5%	-46.22 [-57.04, -35.40]	2013
Lujan et al, 2013	217.83	103.48	1387	186.38	106.35	3018	1.6%	31.45 [24.81, 38.09]	2013
Lee et al, 2013	220.98	93.24	80	183.81	81.94	80	1.0%	37.17 [9.97, 64.37]	2013
Keller et al, 2014	267.4	76.4	62	281.4	74.6	19	0.8%	-14.00 [-52.56, 24.56]	2014
Zhou et al, 2014	167.064	48.842	57	145.784	46.619	65	1.3%	21.28 [4.27, 38.29]	2014
Wang et al, 2014	212.1	52.2	100	206.2	62	136	1.4%	5.90 [-8.70, 20.50]	2014
Inomata et al, 2014	352	282.25	38	265	103.75	27	0.2%	87.00 [-10.90, 184.90]	2014
Inada et al, 2014	277	38.837	14	242.5	56.954	14	0.8%	34.50 [-1.61, 70.61]	2014
Kusano et al, 2014	476.5	92.5	19	368.25	105.862	14	0.4%	108.25 [38.93, 177.57]	2014
Guo et al, 2015	170	18.333	191	150	13.333	191	1.6%	20.00 [16.79, 23.21]	2015
Chi et al, 2015	231	52	89	189	42	48	1.3%	42.00 [25.94, 58.06]	2015
Wang et al, 2015	180.83	47.83	106	172.07	49.16	106	1.4%	8.76 [-4.30, 21.82]	2015
Zhou et al, 2015	188.9	67.7	152	188.7	65.4	254	1.4%	0.20 [-13.24, 13.64]	2015
Zeng et al, 2015	220	43.333	112	200	33.333	182	1.5%	20.00 [10.63, 29.37]	2015
Li et al, 2015	177.9	50.5	129	170.7	62.3	152	1.4%	7.20 [-5.99, 20.39]	2015
Huang et al, 2015	143.89	50.865	492	164.86	67.993	424	1.5%	-20.97 [-28.85, -13.09]	2015
Chen W et al, 2016	217.4	69.6	75	228.8	56.3	97	1.3%	-11.40 [-30.73, 7.93]	2016
Kim et al, 2016	127	35	486	126	43	1095	1.6%	1.00 [-3.02, 5.02]	2016
Nonaka et al, 2016	316.8	103.4	40	345.3	135.6	38	0.5%	-28.50 [-82.22, 25.22]	2016
Chen Z et al, 2016	235.4	83.8	37	207.7	67.9	37	0.8%	27.70 [-7.05, 62.45]	2016
Matsuhashi et al, 2017	400.8	81.25	19	437.25	114.32	6	0.2%	-36.45 [-134.95, 62.05]	2017
De'Angelis et al, 2017	244.5	80	52	250	63.75	52	1.0%	-5.50 [-33.30, 22.30]	2017
Wu et al, 2017	254.46	61.01	112	215.21	56.54	121	1.4%	39.25 [24.11, 54.39]	2017
Wu et al, 2018	165	6.667	277	140	13.333	614	1.6%	25.00 [23.69, 26.31]	2017
Silva-Velazco et al, 2017	239	68.167	118	184	77.667	304	1.4%	55.00 [39.92, 70.08]	2017
Hida et al, 2018	330	28	482	326	27.833	482	1.6%	4.00 [0.48, 7.52]	2018
Garfinkle et al, 2018	282	114	34	198	78	201	0.7%	84.00 [44.19, 123.81]	2018
Tayar et al, 2018	289.247	65.644	50	249.831	77.857	50	1.0%	39.42 [11.19, 67.64]	2018
Davis et al, 2019	276.1	115.7	12335	246.8	127.9	19092	1.6%	29.30 [26.57, 32.03]	2019
Chen et al, 2019	184	55	64	153	42	23	1.2%	31.00 [9.18, 52.82]	2019
Zhang X et al, 2019	160	47.5	86	172.5	88.75	39	1.0%	-12.50 [-42.11, 17.11]	2019
Mar et al, 2020	242.11	157.82	400	265.84	180.55	201	1.0%	-23.73 [-53.09, 5.63]	2020
**Subtotal (95% CI)**			**21640**			**35033**	**76.6%**	**22.45 [17.10, 27.79]**	
Heterogeneity: Tau² = 355.91; Chi² = 1838.86, df = 64 (P < 0.00001); I² = 97%									
Test for overall effect: Z = 8.23 (P < 0.00001)									
**Total (95% CI)**			**24106**			**37127**	**100.0%**	**25.67 [21.02, 30.32]**	

Heterogeneity: Tau² = 350.21; Chi² = 2540.83, df = 81 (P < 0.00001); I² = 97%
Test for overall effect: Z = 10.82 (P < 0.00001)
Test for subgroup differences: Chi² = 5.90, df = 1 (P = 0.02), I² = 83.1%

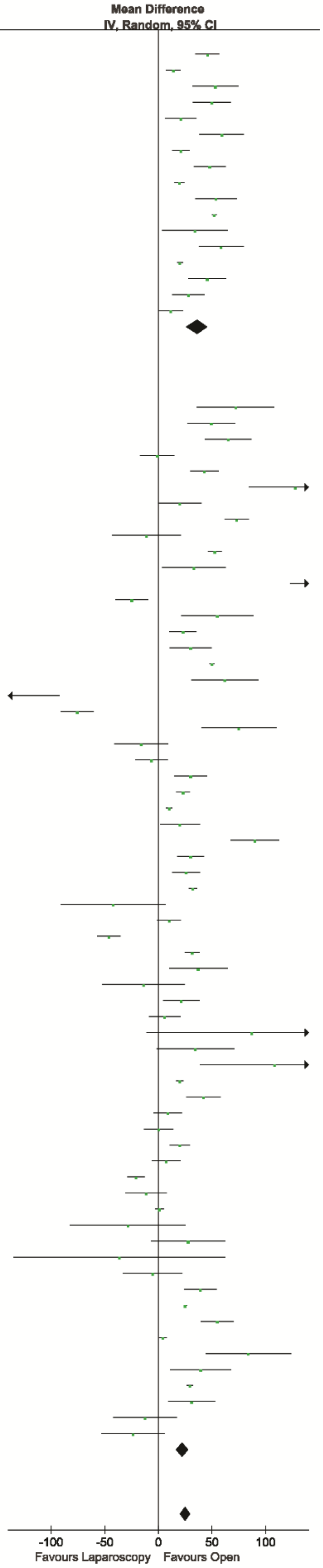

FIGURE S2 Forest plot for blood loss

	Laparoscopic surgery			Open surgery				Mean Difference	
Study or Subgroup	Mean	SD	Total	Mean	SD	Total	Weight	IV, Random, 95% CI	Year
**RCT**									
Leung et al, 2004	169	500	203	238	972.667	200	1.1%	-69.00 [-220.34, 82.34]	2003
Zhou et al, 2004	20	19.167	82	92	25	89	2.1%	-72.00 [-78.65, -65.35]	2004
Braga et al, 2007	213	236	83	396	367	85	1.5%	-183.00 [-276.09, -89.91]	2007
Ng et al, 2008	321.7	750	51	555.6	1,180	48	0.3%	-233.90 [-626.08, 158.28]	2008
Lujan et al, 2009	127.8	113.3	101	234.2	174.3	103	2.0%	-106.40 [-146.67, -66.13]	2009
Ng et al, 2009	280	500	76	337.3	423.667	77	1.1%	-57.30 [-204.24, 89.64]	2009
COREAN, 2010	200	33.333	170	217.5	41.667	170	2.1%	-17.50 [-25.52, -9.48]	2010
Liu et al, 2010	310	96	98	380	85	88	2.0%	-70.00 [-96.01, -43.99]	2010
Gong et al, 2012	86.9	37.6	67	119.1	32.7	71	2.1%	-32.20 [-43.99, -20.41]	2012
COLOR II, 2013	200	50	699	400	83.333	345	2.1%	-200.00 [-209.54, -190.46]	2013
Ng et al, 2014	141.8	500	40	361.1	623.75	40	0.6%	-219.30 [-467.04, 28.44]	2014
EnROL, 2014	181	146	29	450	397	27	1.0%	-269.00 [-427.90, -110.10]	2014
ALaCaRT, 2015	100	25	238	150	40.833	235	2.1%	-50.00 [-56.11, -43.89]	2015
Z6051, 2015	256.1	305.8	240	318.4	331.7	222	1.8%	-62.30 [-120.62, -3.98]	2015
Yao et al, 2018	135.2	60.4	60	232.5	116.3	60	2.0%	-97.30 [-130.46, -64.14]	2018
Alhanafy et al, 2020	400.5	269.9	60	840	347	60	1.4%	-439.50 [-550.73, -328.27]	2020
**Subtotal (95% CI)**			**2297**			**1920**	**25.1%**	**-110.85 [-147.23, -74.47]**	
Heterogeneity: Tau² = 3859.88; Chi² = 1020.36, df = 15 (P < 0.00001); I² = 99%									
Test for overall effect: Z = 5.97 (P < 0.00001)									
**NRCT**									
Leung et al, 2000	1,025	725	25	1,000	681.25	34	0.3%	25.00 [-339.97, 389.97]	2000
Wu et al, 2004	136	21	18	357	34	18	2.1%	-221.00 [-239.46, -202.54]	2004
Breukink et al, 2005	250	755	41	1,000	862.5	41	0.3%	-750.00 [-1100.87, -399.13]	2005
Sahakitrungruang et al, 2005	426.5	237.5	24	1,351.5	987.5	25	0.3%	-925.00 [-1323.58, -526.42]	2005
Law et al, 2006	250	391.667	98	200	175	167	1.6%	50.00 [-31.96, 131.96]	2006
Wong et al, 2006	200	246.667	71	400	1,475	31	0.2%	-200.00 [-722.39, 322.39]	2006
Veenhof et al, 2007	350	137.5	50	800	122.5	50	1.9%	-450.00 [-501.04, -398.96]	2007
Staudacher et al, 2007	208	182	108	356	292	79	1.7%	-148.00 [-220.97, -75.03]	2007
Law et al, 2009	200	50	111	350	66.667	310	2.1%	-150.00 [-161.90, -138.10]	2009
Khaikin et al, 2009	297	612.5	32	268	287.5	50	0.7%	29.00 [-197.69, 255.69]	2009
Ding et al, 2009	140	135	115	200	405	220	1.8%	-60.00 [-118.93, -1.07]	2009
González et al, 2009	139.2	67.5	28	231.8	77.5	28	2.0%	-92.60 [-130.67, -54.53]	2009
Denoya et al, 2010	313	443	32	279	229	32	0.9%	34.00 [-138.78, 206.78]	2010
Fujimoto et al, 2010	40	185	35	1,087.5	852.5	19	0.3%	-1047.50 [-1435.69, -659.31]	2010
Park et al, 2011	59.1	113.4	130	155.2	267.2	80	1.8%	-96.10 [-157.81, -34.39]	2011
Baik et al, 2011	313.2	260.9	54	420.6	314.7	108	1.5%	-107.40 [-198.86, -15.94]	2011
Li et al, 2011	50	200	113	150	183.333	123	1.9%	-100.00 [-149.09, -50.91]	2011
Yamamoto et al, 2011	200	108	22	513	258	22	1.3%	-313.00 [-429.87, -196.13]	2011
Seshadri et al, 2012	200	83.333	72	400	225	72	1.8%	-200.00 [-255.42, -144.58]	2012
Li et al, 2012	58.3	13.9	381	161.9	32.3	276	2.1%	-103.60 [-107.66, -99.54]	2012
Kellokumpu et al, 2012	175	47.833	100	500	93	91	2.0%	-325.00 [-346.28, -303.72]	2012
Siani et al, 2012	263	166	30	505	205	30	1.5%	-242.00 [-336.39, -147.61]	2012
Lee et al, 2013	132.3781	113.2442	80	200	150.9922	80	1.9%	-67.62 [-108.98, -26.26]	2013
Yang et al, 2013	28	5	87	80	20	90	2.1%	-52.00 [-56.26, -47.74]	2013
Kuo et al, 2013	103.6	55	28	265	175	30	1.8%	-161.40 [-227.25, -95.55]	2013
Park et al, 2013	158.7	83.1	406	204.2	91.7	406	2.1%	-45.50 [-57.54, -33.46]	2013
Inada et al, 2014	111.75	69.598	14	396.75	331.115	14	0.9%	-285.00 [-462.24, -107.76]	2014
Inomata et al, 2014	155	498.75	38	360	692.5	27	0.4%	-205.00 [-510.57, 100.57]	2014
Wang et al, 2014	189.6	150.9	100	296.3	274.3	136	1.9%	-106.70 [-161.47, -51.93]	2014
Keller et al, 2014	152.4	271.6	62	521.6	690.1	19	0.4%	-369.20 [-686.78, -51.62]	2014
Zhou et al, 2014	138.842	123.844	57	188.662	172.622	65	1.9%	-49.82 [-102.69, 3.05]	2014
Chi et al, 2015	50	48.333	89	100	120	48	2.0%	-50.00 [-85.40, -14.60]	2015
Zeng et al, 2015	100	128.333	112	200	245	182	1.9%	-100.00 [-142.80, -57.20]	2015
Huang et al, 2015	111.54	97.148	492	154.03	154.545	424	2.1%	-42.49 [-59.52, -25.46]	2015
Wang et al, 2015	93.87	60.04	106	88.44	55.15	106	2.1%	5.43 [-10.09, 20.95]	2015
Li et al, 2015	50	166.667	129	150	200	152	1.9%	-100.00 [-142.87, -57.13]	2015
Guo et al, 2015	170	28.333	191	250	25	191	2.1%	-80.00 [-85.36, -74.64]	2015
Zhou et al, 2015	50	246.667	152	200	490	254	1.7%	-150.00 [-221.90, -78.10]	2015
Chen Z et al, 2016	100	242.5	37	200	742.5	37	0.6%	-100.00 [-351.68, 151.68]	2016
Nonaka et al, 2016	119.8	136	40	429	251.6	38	1.5%	-309.20 [-399.62, -218.78]	2016
Chen W et al, 2016	156.3	89.1	75	214.1	185.4	97	1.9%	-57.80 [-99.85, -15.75]	2016
Wu et al, 2017	300.27	217.5	112	387.85	326.8	121	1.7%	-87.58 [-158.38, -16.78]	2017
De'Angelis et al, 2017	120	120	52	150	367.5	52	1.4%	-30.00 [-135.08, 75.08]	2017
Wu et al, 2018	20	1.667	277	40	10	614	2.1%	-20.00 [-20.81, -19.19]	2017
Silva-Velazco et al, 2017	200	497.5	118	300	2,682.5	304	0.4%	-100.00 [-414.62, 214.62]	2017
Matsuhashi et al, 2017	582.1	440	19	1,095.4	442.022	6	0.3%	-513.30 [-918.56, -108.04]	2017
Hida et al, 2018	90	30.833	482	603	131.333	482	2.1%	-513.00 [-525.04, -500.96]	2018
Chen et al, 2019	42	59	64	113	129	23	1.9%	-71.00 [-125.67, -16.33]	2019
Zhang X et al, 2019	20	49.167	86	80	172.5	39	1.8%	-60.00 [-115.13, -4.87]	2019
Zhang Z et al, 2019	194.2	137.84	112	234.95	140.44	116	2.0%	-40.75 [-76.87, -4.63]	2019
**Subtotal (95% CI)**			**5307**			**6059**	**74.9%**	**-138.53 [-166.66, -110.40]**	
Heterogeneity: Tau² = 7620.87; Chi² = 10584.64, df = 49 (P < 0.00001); I² = 100%									
Test for overall effect: Z = 9.65 (P < 0.00001)									
**Total (95% CI)**			**7604**			**7979**	**100.0%**	**-131.42 [-153.61, -109.23]**	
Heterogeneity: Tau² = 6143.51; Chi² = 12120.20, df = 65 (P < 0.00001); I² = 99%									
Test for overall effect: Z = 11.61 (P < 0.00001)									
Test for subgroup differences: Chi² = 1.39, df = 1 (P = 0.24), I² = 28.2%									

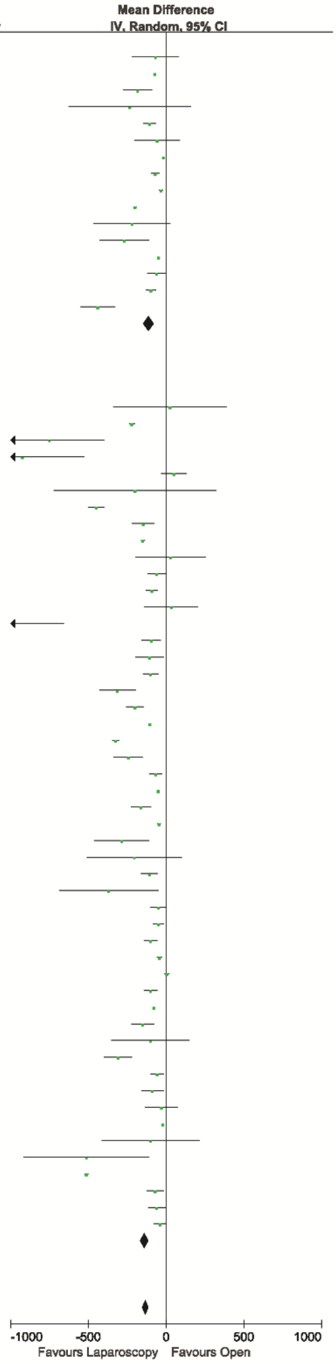

FIGURE S3 Forest plot for incision length

Study or Subgroup	Laparoscopic surgery Mean	SD	Total	Open surgery Mean	SD	Total	Weight	Mean Difference IV, Fixed, 95% CI	Year
**RCT**									
Braga et al, 2007	5.8	0.8	83	19.1	3.1	85	0.2%	-13.30 [-13.98, -12.62]	2007
COREAN, 2010	5	0.25	170	20	0.833	170	5.6%	-15.00 [-15.13, -14.87]	2010
Liu et al, 2010	6	1	98	17	2	88	0.4%	-11.00 [-11.46, -10.54]	2010
EnROL, 2014	7.6	5	29	21.7	7.4	27	0.0%	-14.10 [-17.43, -10.77]	2014
Z6051, 2015	7	5.7	240	16.5	8.4	222	0.1%	-9.50 [-10.82, -8.18]	2015
ALaCaRT, 2015	6	0.75	238	13	1	235	3.7%	-7.00 [-7.16, -6.84]	2015
Alhanafy et al, 2020	7.17	2.36	60	20.3	6.05	60	0.0%	-13.13 [-14.77, -11.49]	2020
**Subtotal (95% CI)**			**918**			**887**	**10.0%**	**-11.77 [-11.87, -11.68]**	
Heterogeneity: Chi² = 5828.36, df = 6 (P < 0.00001); I² = 100%									
Test for overall effect: Z = 237.27 (P < 0.00001)									
**NRCT**									
Ding et al, 2009	4	6.75	115	20	2.7	220	0.1%	-16.00 [-17.28, -14.72]	2009
Koulas et al, 2009	7.5	1	57	19	1.5	60	0.4%	-11.50 [-11.96, -11.04]	2009
Huang et al, 2015	4.14	0.738	492	13.8	2.603	424	1.4%	-9.66 [-9.92, -9.40]	2015
Wu et al, 2018	5	0.167	277	18	0.333	614	88.0%	-13.00 [-13.03, -12.97]	2018
Zhang X et al, 2019	5	3.833	86	20	7.5	39	0.0%	-15.00 [-17.49, -12.51]	2019
**Subtotal (95% CI)**			**1027**			**1357**	**90.0%**	**-12.94 [-12.97, -12.91]**	
Heterogeneity: Chi² = 704.52, df = 4 (P < 0.00001); I² = 99%									
Test for overall effect: Z = 780.20 (P < 0.00001)									
**Total (95% CI)**			**1945**			**2244**	**100.0%**	**-12.82 [-12.85, -12.79]**	
Heterogeneity: Chi² = 7029.86, df = 11 (P < 0.00001); I² = 100%									
Test for overall effect: Z = 815.18 (P < 0.00001)									
Test for subgroup differences: Chi² = 496.98, df = 1 (P < 0.00001), I² = 99.8%									

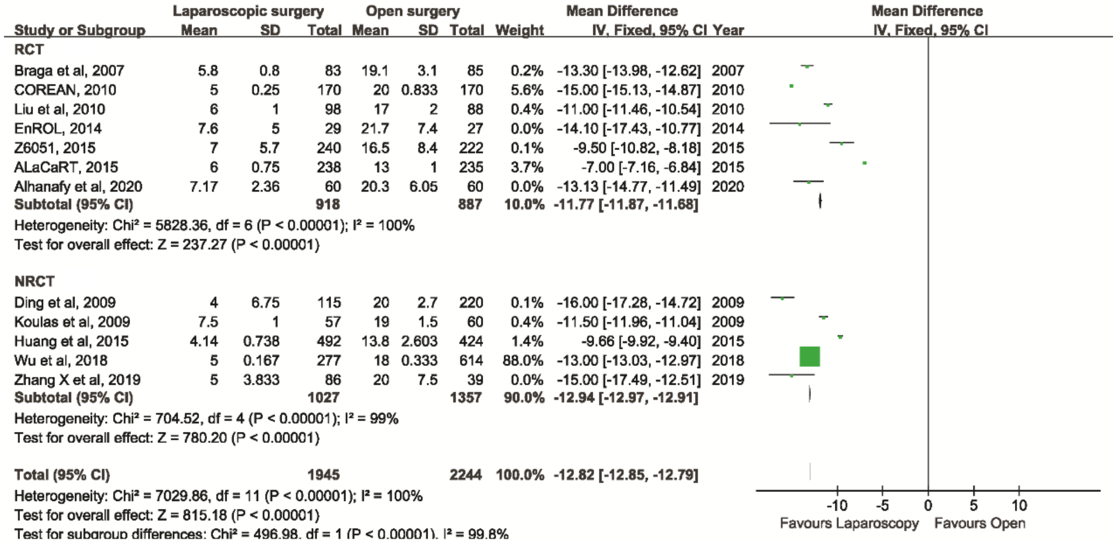

FIGURE S4 Forest plot for the number of harvested lymph nodes

Study or Subgroup	Laparoscopic surgery Mean	SD	Total	Open surgery Mean	SD	Total	Weight	Mean Difference IV, Random, 95% CI	Year
**RCT**									
Araujo et al, 2003	5	5	13	11	9	15	0.3%	-6.00 [-11.30, -0.70]	2003
Leung et al, 2004	11.1	7.9	203	12.1	7.1	200	1.5%	-1.00 [-2.47, 0.47]	2003
Braga et al, 2007	12.7	7.3	83	13.6	6.9	85	1.1%	-0.90 [-3.05, 1.25]	2007
Pechlivanides et al, 2007	19.2	8.25	34	19.2	10	39	0.5%	0.00 [-4.19, 4.19]	2007
Ng et al, 2008	12.4	6.7	51	13	7	48	0.9%	-0.60 [-3.30, 2.10]	2008
Ng et al, 2009	11.5	7.9	76	12	7	77	1.0%	-0.50 [-2.87, 1.87]	2009
Lujan et al, 2009	13.63	6.26	101	11.57	5.1	103	1.5%	2.06 [0.49, 3.63]	2009
COREAN, 2010	17	1.667	170	18	1.833	170	2.0%	-1.00 [-1.37, -0.63]	2010
Liu et al, 2010	16	1	98	15	1.667	88	2.0%	1.00 [0.60, 1.40]	2010
Liang et al, 2011	7.05	5.05	169	7.44	4.89	174	1.7%	-0.39 [-1.44, 0.66]	2011
Gong et al, 2012	20.3	8.3	67	21.1	6.7	71	1.0%	-0.80 [-3.33, 1.73]	2012
COLOR II, 2013	13	1.333	683	14	1.5	341	2.1%	-1.00 [-1.19, -0.81]	2013
EnROL, 2014	18.8	9.7	29	17.3	6.8	27	0.5%	1.50 [-2.86, 5.86]	2014
Ng et al, 2014	17.7	8.4	40	14.8	5.6	40	0.8%	2.90 [-0.23, 6.03]	2014
Z6051, 2015	17.9	10.1	240	16.5	8.4	222	1.4%	1.40 [-0.29, 3.09]	2015
Yao et al, 2018	6.3	1.7	60	6.5	2.1	60	1.9%	-0.20 [-0.88, 0.48]	2018
Alhanafy et al, 2020	24.9	3.59	60	22.5	5.91	60	1.4%	2.40 [0.65, 4.15]	2020
**Subtotal (95% CI)**			**2177**			**1820**	**21.7%**	**0.05 [-0.59, 0.69]**	
Heterogeneity: Tau² = 0.98; Chi² = 122.42, df = 16 (P < 0.00001); I² = 87%									
Test for overall effect: Z = 0.16 (P = 0.87)									
**NRCT**									
Schwandner et al, 1999	12.9	5.1	32	13	4.4	32	1.1%	-0.10 [-2.43, 2.23]	1999
Leung et al, 2000	10	7.25	25	12	10	34	0.5%	-2.00 [-6.40, 2.40]	2000
Hartley et al, 2001	6.2695	4.969	21	7.3589	4.7549	22	0.8%	-1.09 [-4.00, 1.82]	2001
Anthuber et al, 2003	15.3	5.5	101	21.9	12.9	334	1.4%	-6.60 [-8.35, -4.85]	2003
Wu et al, 2004	7.8	1.7	18	8.2	2.3	18	1.6%	-0.40 [-1.72, 0.92]	2004
Breukink et al, 2005	8	6	41	8	4.5	41	1.1%	0.00 [-2.30, 2.30]	2005
Sahakitrungruang et al, 2005	21.2	9.37	24	24.44	13.84	25	0.2%	-3.24 [-9.83, 3.35]	2005
Bretagnol et al, 2005	10	7	144	12	9.5	144	1.3%	-2.00 [-3.93, -0.07]	2005
Law et al, 2006	10	6.167	98	11	4.167	167	1.6%	-1.00 [-2.37, 0.37]	2006
Lelong et al, 2007	11.2	4.7	104	10.6	5.2	68	1.5%	0.60 [-0.93, 2.13]	2007
Veenhof et al, 2007	7	2.25	50	6	1.75	50	1.9%	1.00 [0.21, 1.79]	2007
Ströhlein et al, 2008	13.5	5.5	114	16.9	6.6	275	1.6%	-3.40 [-4.68, -2.12]	2008
Gouvas et al, 2009	18.9	6.7	45	19.8	8.7	43	0.7%	-0.90 [-4.15, 2.35]	2009
Koulas et al, 2009	14	7.16	57	15.55	5.48	60	1.1%	-1.55 [-3.87, 0.77]	2009
González et al, 2009	12.1	2	28	9.3	3	28	1.6%	2.80 [1.46, 4.14]	2009
Ding et al, 2009	8	8.1	115	9	10.8	220	1.2%	-1.00 [-3.06, 1.06]	2009
Khaikin et al, 2009	18.7	12.25	32	19.7	23.25	50	0.2%	-1.00 [-8.72, 6.72]	2009
Biondo et al, 2010	14.77	8.333	209	17.16	13.333	395	1.4%	-2.39 [-4.12, -0.66]	2010
Denoya et al, 2010	19	9	32	19	9	32	0.5%	0.00 [-4.41, 4.41]	2010
Fujimoto et al, 2010	13	11	35	27	14	19	0.2%	-14.00 [-21.27, -6.73]	2010
da Luz Moreira et al, 2011	17	9.5	91	16	8	91	1.0%	1.00 [-1.55, 3.55]	2011
Li et al, 2011	20.3	6.9	113	20.6	8.7	123	1.2%	-0.30 [-2.30, 1.70]	2011
Baik et al, 2011	10.7	4.6	54	11.2	6	108	1.4%	-0.50 [-2.17, 1.17]	2011
Park et al, 2011	14.8	9.2	130	16.5	10.6	80	0.9%	-1.70 [-4.51, 1.11]	2011
Yamamoto et al, 2011	14	6	22	15.5	6	22	0.6%	-1.50 [-5.05, 2.05]	2011
Jun et al, 2011	15.9	10.1	123	18.5	10.9	88	0.8%	-2.60 [-5.49, 0.29]	2011
Jefferies et al, 2012	16.25	7.25	16	16.25	7.25	25	0.4%	0.00 [-4.55, 4.55]	2012
Seshadri et al, 2012	7	3.833	72	7	4	72	1.6%	0.00 [-1.28, 1.28]	2012
Mckay et al, 2012	16.2	10.2	157	15.5	9.6	388	1.3%	0.70 [-1.16, 2.56]	2012
Li et al, 2012	12.5	4.2	381	13.1	5.4	276	1.9%	-0.60 [-1.36, 0.16]	2012
Gunka et al, 2012	14.2	6.5	75	11.2	8.75	70	1.0%	3.00 [0.48, 5.52]	2012
Kellokumpu et al, 2012	11	1.5	100	13	0.967	91	2.0%	-2.00 [-2.35, -1.65]	2012
Siani et al, 2012	24	12	30	26	14	30	0.2%	-2.00 [-8.60, 4.60]	2012
Lujan et al, 2013	14.53	8.36	1387	14.75	9.49	3018	2.0%	-0.22 [-0.78, 0.34]	2013
Kuo et al, 2013	13.9	6.5	28	14.7	10	30	0.5%	-0.80 [-5.11, 3.51]	2013
Lee et al, 2013	20.3524	8.3046	80	22.5	9.8145	80	0.9%	-2.15 [-4.96, 0.67]	2013
Yang et al, 2013	14	5	87	13	6	90	1.4%	1.00 [-0.62, 2.62]	2013
Penninckx et al, 2013	11	1.5	764	11	1.333	1896	2.1%	0.00 [-0.12, 0.12]	2013
Park et al, 2013	17.3	4.3	406	12.7	2.8	406	2.0%	4.60 [4.10, 5.10]	2013
Keller et al, 2014	20.7	10.3	62	25.5	17.8	19	0.2%	-4.80 [-13.20, 3.60]	2014
Ferko et al, 2014	14	13.25	53	16	8.167	72	0.5%	-2.00 [-6.04, 2.04]	2014
Wang et al, 2014	14.8	7.8	100	15.6	7.3	136	1.2%	-0.80 [-2.76, 1.16]	2014
Inada et al, 2014	18.5	5.605	14	23.75	9.558	14	0.3%	-5.25 [-11.05, 0.55]	2014
Zhou et al, 2014	13.737	7.738	57	15.354	6.802	65	0.9%	-1.62 [-4.22, 0.98]	2014
Wang et al, 2015	12.88	6.93	106	12.91	5.4	106	1.4%	-0.03 [-1.70, 1.64]	2015
Zeng et al, 2015	14	5.5	112	15	6.833	182	1.5%	-1.00 [-2.42, 0.42]	2015
Li et al, 2015	20.2	7.1	129	20.2	8.3	152	1.3%	0.00 [-1.80, 1.80]	2015
Huang et al, 2015	11.09	6.503	492	10.68	6.321	424	1.9%	0.41 [-0.42, 1.24]	2015
Nussbaum et al, 2015	15	1.667	6430	15	1.5	6430	2.1%	0.00 [-0.05, 0.05]	2015
Zhou et al, 2015	12.9	6.1	152	12.2	6.2	254	1.6%	0.70 [-0.53, 1.93]	2015
Kim et al, 2015	12	13.833	126	13	8	172	0.9%	-1.00 [-3.70, 1.70]	2015
Chi et al, 2015	15.1	6.3	89	15.6	7.8	48	1.0%	-0.50 [-3.07, 2.07]	2015
Guo et al, 2015	16	2	191	16	3	191	2.0%	0.00 [-0.51, 0.51]	2015
Chen W et al, 2016	13.8	5.1	75	12.6	5.9	97	1.4%	1.20 [-0.45, 2.85]	2016
Keskin et al, 2016	24	13	437	22	15	150	0.9%	2.00 [-0.69, 4.69]	2016
Kim et al, 2016	20.9	8.5	486	21.3	9.5	1095	1.8%	-0.40 [-1.34, 0.54]	2016
Odermatt et al, 2016	12	16.25	48	11.5	4.75	48	0.4%	0.50 [-4.29, 5.29]	2016
Allaix et al, 2016	12	3.667	153	11.5	6.5	154	1.7%	0.50 [-0.68, 1.68]	2016
Wu et al, 2017	9.82	5.06	112	12.23	7.71	121	1.4%	-2.41 [-4.07, -0.75]	2017
Silva-Velazco et al, 2017	24	21	118	23	33.5	304	0.3%	1.00 [-4.34, 6.34]	2017
Matsuhashi et al, 2017	10.35	4.25	19	28	12.124	6	0.1%	-17.65 [-27.54, -7.76]	2017
De'Angelis et al, 2017	20.98	13.21	52	19.36	10.53	52	0.4%	1.62 [-2.97, 6.21]	2017
Wu et al, 2018	11	1.5	277	9	1.333	614	2.1%	2.00 [1.79, 2.21]	2017
Aydın et al, 2018	14.5	6.6	71	14.55	4.55	50	1.2%	-0.05 [-2.04, 1.94]	2018
Hida et al, 2018	14	2	482	17	2.667	482	2.1%	-3.00 [-3.30, -2.70]	2018
Chen et al, 2019	18.8	8.1	64	23.5	8.2	23	0.6%	-4.70 [-8.59, -0.81]	2019
Zimmermann et al, 2019	14	6	248	22	8.5	248	1.6%	-8.00 [-9.29, -6.71]	2019
Zhang X et al, 2019	12	5.833	86	9	8.5	39	0.8%	3.00 [0.06, 5.94]	2019
**Subtotal (95% CI)**			**16282**			**20789**	**78.3%**	**-0.60 [-1.01, -0.19]**	
Heterogeneity: Tau² = 1.71; Chi² = 1554.69, df = 67 (P < 0.00001); I² = 96%									
Test for overall effect: Z = 2.88 (P = 0.004)									
**Total (95% CI)**			**18459**			**22609**	**100.0%**	**-0.44 [-0.78, -0.10]**	
Heterogeneity: Tau² = 1.46; Chi² = 1740.08, df = 84 (P < 0.00001); I² = 95%									
Test for overall effect: Z = 2.53 (P = 0.01)									
Test for subgroup differences: Chi² = 2.85, df = 1 (P = 0.09), I² = 65.0%									

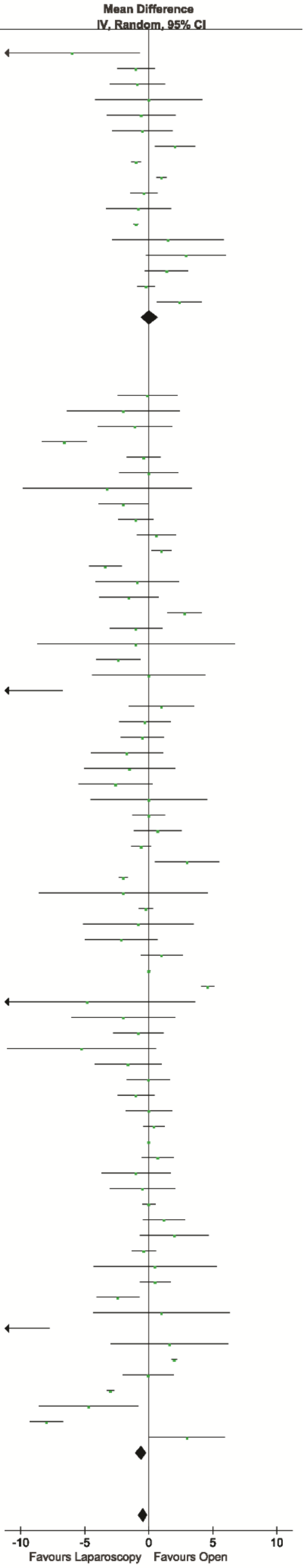

FIGURE S5 Forest plot for CMR positive rate

Study or Subgroup	Laparoscopic surgery Events	Total	Open surgery Events	Total	Weight	Risk Ratio M-H, Fixed, 95% CI	Year
**RCT**							
Braga et al, 2007	1	83	2	85	0.2%	0.51 [0.05, 5.54]	2007
Ng et al, 2008	3	51	2	48	0.2%	1.41 [0.25, 8.09]	2008
Ng et al, 2009	2	76	1	77	0.1%	2.03 [0.19, 21.88]	2009
Lujan et al, 2009	4	101	3	103	0.2%	1.36 [0.31, 5.92]	2009
COREAN, 2010	5	170	7	170	0.6%	0.71 [0.23, 2.21]	2010
Gong et al, 2012	1	67	2	71	0.2%	0.53 [0.05, 5.71]	2012
COLOR II, 2013	56	588	30	300	3.3%	0.95 [0.63, 1.45]	2013
Ng et al, 2014	3	40	2	40	0.2%	1.50 [0.26, 8.50]	2014
Z6051, 2015	29	240	17	222	1.5%	1.58 [0.89, 2.79]	2015
ALaCaRT, 2015	16	238	7	235	0.6%	2.26 [0.95, 5.39]	2015
**Subtotal (95% CI)**		**1654**		**1351**	**7.0%**	**1.21 [0.91, 1.59]**	
Total events	120		73				
Heterogeneity: Chi² = 6.14, df = 9 (P = 0.73); I² = 0%							
Test for overall effect: Z = 1.32 (P = 0.19)							
**NRCT**							
Bretagnol et al, 2005	9	144	8	144	0.7%	1.13 [0.45, 2.83]	2005
Breukink et al, 2005	3	41	5	41	0.4%	0.60 [0.15, 2.35]	2005
Lelong et al, 2007	8	104	10	68	1.0%	0.52 [0.22, 1.26]	2007
Gouvas et al, 2009	1	45	4	43	0.3%	0.24 [0.03, 2.05]	2009
Laurent et al, 2009	16	238	11	233	0.9%	1.42 [0.68, 3.00]	2009
Biondo et al, 2010	20	209	50	395	2.9%	0.76 [0.46, 1.23]	2010
Jun et al, 2011	3	123	2	88	0.2%	1.07 [0.18, 6.29]	2011
Park et al, 2011	3	130	4	80	0.4%	0.46 [0.11, 2.01]	2011
Kellokumpu et al, 2012	5	100	3	91	0.3%	1.52 [0.37, 6.17]	2012
Laurent et al, 2012	17	110	4	65	0.4%	2.51 [0.88, 7.14]	2012
Seshadri et al, 2012	1	72	7	72	0.6%	0.14 [0.02, 1.13]	2012
Lujan et al, 2013	132	1387	492	3018	25.9%	0.58 [0.49, 0.70]	2013
Kuo et al, 2013	4	28	2	30	0.2%	2.14 [0.43, 10.80]	2013
Penninckx et al, 2013	95	524	240	1325	11.4%	1.00 [0.81, 1.24]	2013
Keller et al, 2014	2	62	1	19	0.1%	0.61 [0.06, 6.39]	2014
Ferko et al, 2014	11	53	27	72	1.9%	0.55 [0.30, 1.01]	2014
Zhou et al, 2014	5	57	3	65	0.2%	1.90 [0.48, 7.60]	2014
Wang et al, 2014	2	100	3	136	0.2%	0.91 [0.15, 5.33]	2014
Nussbaum et al, 2015	315	6430	428	6430	35.8%	0.74 [0.64, 0.85]	2015
Zeng et al, 2015	2	112	4	182	0.3%	0.81 [0.15, 4.36]	2015
Chi et al, 2015	1	89	2	48	0.2%	0.27 [0.03, 2.90]	2015
Guo et al, 2015	7	191	5	191	0.4%	1.40 [0.45, 4.33]	2015
Kim et al, 2015	4	71	7	129	0.4%	1.04 [0.31, 3.43]	2015
Keskin et al, 2016	33	437	13	150	1.6%	0.87 [0.47, 1.61]	2016
Kim et al, 2016	5	486	26	1095	1.3%	0.43 [0.17, 1.12]	2016
Allaix et al, 2016	2	153	3	154	0.3%	0.67 [0.11, 3.96]	2016
De'Angelis et al, 2017	8	52	7	52	0.6%	1.14 [0.45, 2.92]	2017
Silva-Velazco et al, 2017	4	118	18	304	0.8%	0.57 [0.20, 1.66]	2017
Hida et al, 2018	22	482	21	482	1.8%	1.05 [0.58, 1.88]	2018
Chen et al, 2019	5	64	3	23	0.4%	0.60 [0.16, 2.31]	2019
Zimmermann et al, 2019	14	174	4	229	0.3%	4.61 [1.54, 13.75]	2019
Zhang X et al, 2019	3	86	6	39	0.7%	0.23 [0.06, 0.86]	2019
**Subtotal (95% CI)**		**12472**		**15493**	**93.0%**	**0.76 [0.69, 0.82]**	
Total events	762		1423				
Heterogeneity: Chi² = 52.97, df = 31 (P = 0.008); I² = 41%							
Test for overall effect: Z = 6.36 (P < 0.00001)							
**Total (95% CI)**		**14126**		**16844**	**100.0%**	**0.79 [0.72, 0.85]**	
Total events	882		1496				
Heterogeneity: Chi² = 68.04, df = 41 (P = 0.005); I² = 40%							
Test for overall effect: Z = 5.70 (P < 0.00001)							
Test for subgroup differences: Chi² = 9.89, df = 1 (P = 0.002), I² = 89.9%							

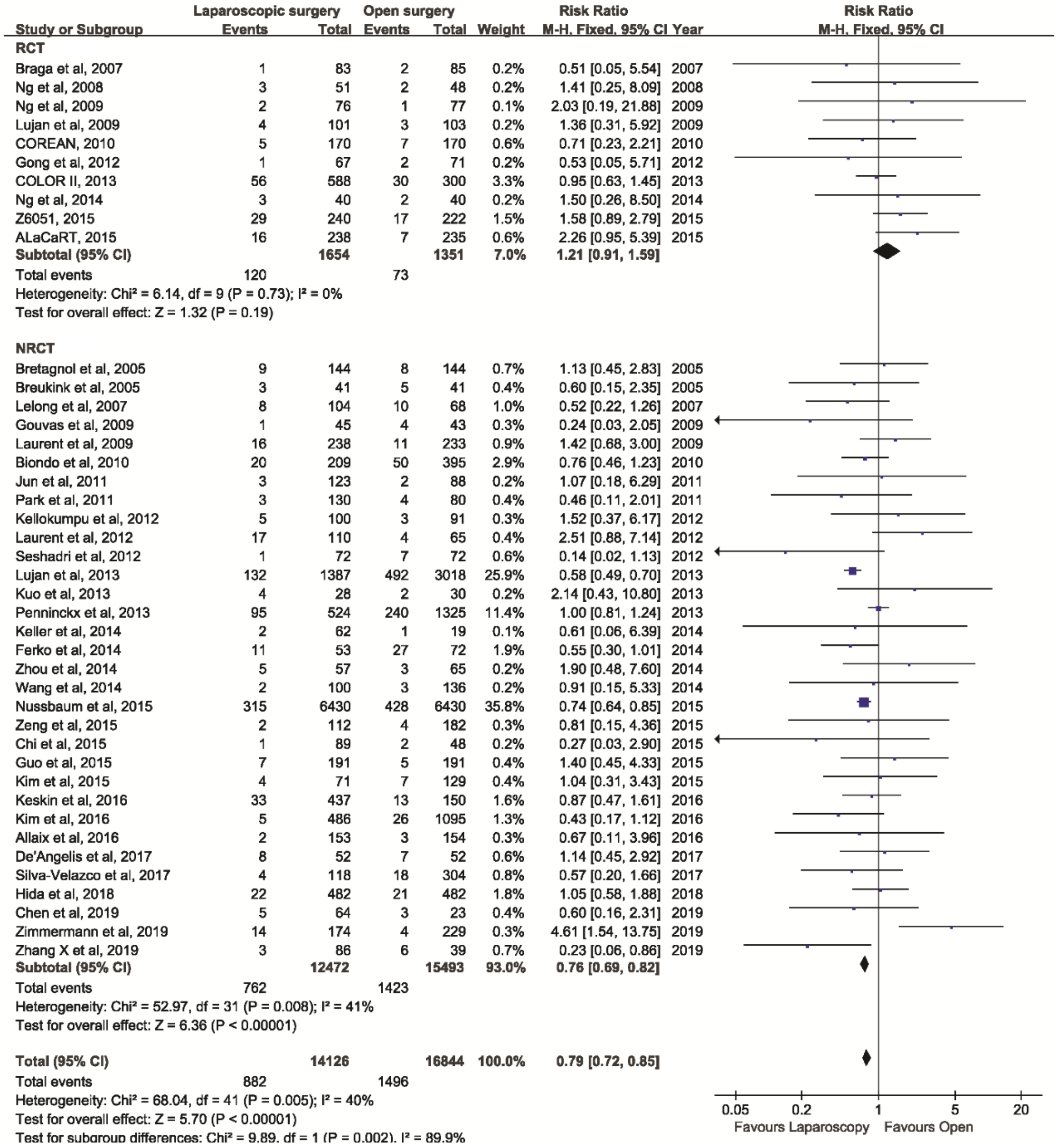

FIGURE S6 Forest plot for DMR positive rate

Study or Subgroup	Laparoscopic surgery Events	Laparoscopic surgery Total	Open surgery Events	Open surgery Total	Weight	Risk Ratio M-H, Fixed, 95% CI	Year
**RCT**							
ALaCaRT, 2015	2	238	1	235	0.2%	1.97 [0.18, 21.63]	2015
Z6051, 2015	4	240	4	222	0.8%	0.93 [0.23, 3.65]	2015
**Subtotal (95% CI)**		**478**		**457**	**1.0%**	**1.13 [0.35, 3.66]**	
Total events	6		5				
Heterogeneity: Chi² = 0.29, df = 1 (P = 0.59); I² = 0%							
Test for overall effect: Z = 0.20 (P = 0.84)							
**NRCT**							
Leung et al, 2000	2	25	1	34	0.2%	2.72 [0.26, 28.36]	2000
Bretagnol et al, 2005	3	144	3	144	0.6%	1.00 [0.21, 4.87]	2005
Lelong et al, 2007	3	104	3	104	0.6%	1.00 [0.21, 4.84]	2007
Laurent et al, 2009	7	238	2	233	0.4%	3.43 [0.72, 16.32]	2009
Gouvas et al, 2009	0	45	2	43	0.5%	0.19 [0.01, 3.87]	2009
Li et al, 2011	3	113	5	123	0.9%	0.65 [0.16, 2.67]	2011
Ferko et al, 2014	3	53	10	72	1.7%	0.41 [0.12, 1.41]	2014
Nussbaum et al, 2015	321	6430	438	6430	85.9%	0.73 [0.64, 0.84]	2015
Zeng et al, 2015	0	112	2	182	0.4%	0.32 [0.02, 6.69]	2015
Kim et al, 2016	6	486	13	1095	1.6%	1.04 [0.40, 2.72]	2016
Keskin et al, 2016	5	437	5	150	1.5%	0.34 [0.10, 1.17]	2016
Silva-Velazco et al, 2017	0	118	4	304	0.5%	0.28 [0.02, 5.25]	2017
De'Angelis et al, 2017	0	52	1	52	0.3%	0.33 [0.01, 8.00]	2017
Manchon-Walsh et al, 2019	17	825	13	505	3.2%	0.80 [0.39, 1.63]	2019
Zhang X et al, 2019	0	86	2	39	0.7%	0.09 [0.00, 1.87]	2019
Zimmermann et al, 2019	5	173	1	244	0.2%	7.05 [0.83, 59.83]	2019
**Subtotal (95% CI)**		**9441**		**9754**	**99.0%**	**0.74 [0.65, 0.85]**	
Total events	375		505				
Heterogeneity: Chi² = 15.97, df = 15 (P = 0.38); I² = 6%							
Test for overall effect: Z = 4.48 (P < 0.00001)							
**Total (95% CI)**		**9919**		**10211**	**100.0%**	**0.75 [0.66, 0.85]**	
Total events	381		510				
Heterogeneity: Chi² = 16.72, df = 17 (P = 0.47); I² = 0%							
Test for overall effect: Z = 4.42 (P < 0.00001)							
Test for subgroup differences: Chi² = 0.48, df = 1 (P = 0.49), I² = 0%							

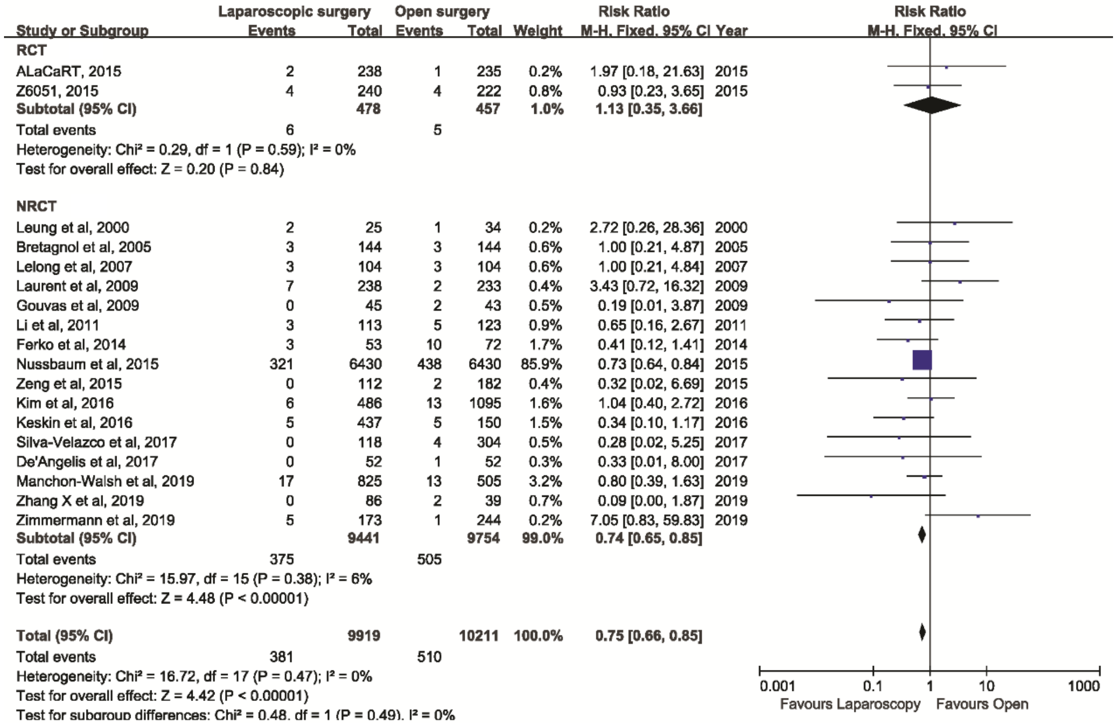

FIGURE S7 Forest plot for macroscopic quality of total mesorectal excision

Study or Subgroup	Laparoscopic surgery Events	Laparoscopic surgery Total	Open surgery Events	Open surgery Total	Weight	Risk Ratio M-H, Random, 95% CI	Year
**RCT**							
COREAN, 2010	123	170	127	170	3.4%	0.97 [0.85, 1.10]	2010
Ng et al, 2014	36	40	37	40	3.1%	0.97 [0.85, 1.11]	2014
EnROL, 2014	11	29	11	27	0.2%	0.93 [0.49, 1.79]	2014
Z6051, 2015	175	240	181	222	4.6%	0.89 [0.81, 0.99]	2015
ALaCaRT, 2015	206	238	216	235	7.1%	0.94 [0.88, 1.00]	2015
Alhanafy et al, 2020	57	60	54	60	4.5%	1.06 [0.95, 1.17]	2020
**Subtotal (95% CI)**		**777**		**754**	**22.9%**	**0.96 [0.91, 1.01]**	
Total events	608		626				
Heterogeneity: Tau² = 0.00; Chi² = 6.04, df = 5 (P = 0.30); I² = 17%							
Test for overall effect: Z = 1.66 (P = 0.10)							
**NRCT**							
Biondo et al, 2010	166	209	298	395	5.2%	1.05 [0.96, 1.15]	2010
Li et al, 2011	106	113	114	123	6.7%	1.01 [0.94, 1.08]	2011
Penninckx et al, 2013	325	524	743	1227	5.8%	1.02 [0.94, 1.11]	2013
Yang et al, 2013	83	87	72	90	3.9%	1.19 [1.06, 1.34]	2013
Lujan et al, 2013	1143	1387	2282	3018	9.7%	1.09 [1.06, 1.12]	2013
Ferko et al, 2014	29	53	32	72	0.6%	1.23 [0.86, 1.76]	2014
Kim et al, 2015	126	131	163	176	7.9%	1.04 [0.98, 1.10]	2015
Zeng et al, 2015	86	112	130	182	3.0%	1.07 [0.94, 1.23]	2015
Li et al, 2015	118	129	141	152	6.6%	0.99 [0.92, 1.06]	2015
Keskin et al, 2016	270	367	54	81	2.3%	1.10 [0.93, 1.30]	2016
De'Angelis et al, 2017	43	52	41	52	1.9%	1.05 [0.87, 1.27]	2017
Silva-Velazco et al, 2017	103	118	267	304	5.8%	0.99 [0.92, 1.08]	2017
Manchon-Walsh et al, 2019	590	744	351	428	7.6%	0.97 [0.91, 1.02]	2019
Zimmermann et al, 2019	88	88	235	241	10.2%	1.02 [1.00, 1.05]	2019
**Subtotal (95% CI)**		**4114**		**6541**	**77.1%**	**1.04 [1.01, 1.07]**	
Total events	3276		4923				
Heterogeneity: Tau² = 0.00; Chi² = 30.67, df = 13 (P = 0.004); I² = 58%							
Test for overall effect: Z = 2.39 (P = 0.02)							
**Total (95% CI)**		**4891**		**7295**	**100.0%**	**1.02 [0.99, 1.05]**	
Total events	3884		5549				
Heterogeneity: Tau² = 0.00; Chi² = 46.96, df = 19 (P = 0.0004); I² = 60%							
Test for overall effect: Z = 1.33 (P = 0.18)							
Test for subgroup differences: Chi² = 7.03, df = 1 (P = 0.008), I² = 85.8%							

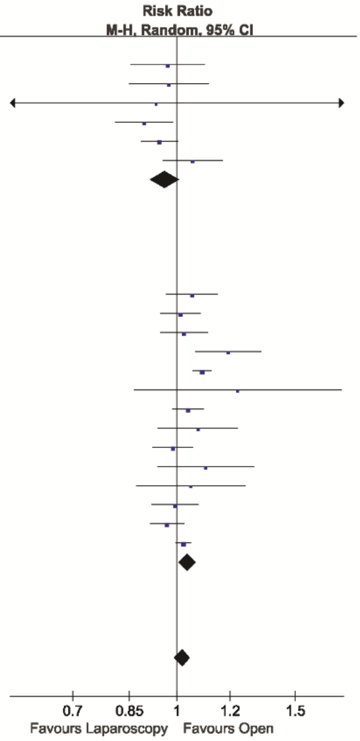

FIGURE S8 Forest plot for bowel movement recovery

Study or Subgroup	Laparoscopic surgery Mean	SD	Total	Open surgery Mean	SD	Total	Weight	Mean Difference IV, Random, 95% CI	Year
**RCT**									
Leung et al, 2004	4	2	203	4.6	2.5	200	2.1%	-0.60 [-1.04, -0.16]	2003
Zhou et al, 2004	1.5	1.3	82	2.7	1.5	89	2.2%	-1.20 [-1.62, -0.78]	2004
CLASICC, 2005	5	0.5	253	6	0.5	128	2.5%	-1.00 [-1.11, -0.89]	2005
Ng et al, 2008	4.3	5.25	51	4.6	2.75	48	0.7%	-0.30 [-1.94, 1.34]	2008
Ng et al, 2009	4.1	1.5	76	4.7	1.833	77	2.0%	-0.60 [-1.13, -0.07]	2009
COREAN, 2010	1.6	0.208	170	2.5	0.208	170	2.5%	-0.90 [-0.94, -0.86]	2010
Liu et al, 2010	3	1	98	4	1	88	2.4%	-1.00 [-1.29, -0.71]	2010
Liang et al, 2011	3.9	0.85	169	4.24	0.788	174	2.5%	-0.34 [-0.51, -0.17]	2011
Gong et al, 2012	1.95	0.62	67	3.98	2.28	71	2.0%	-2.03 [-2.58, -1.48]	2012
COLOR II, 2013	2	0.333	666	3	0.333	337	2.5%	-1.00 [-1.04, -0.96]	2013
Ng et al, 2014	3.1	2	40	3.1	2.25	40	1.4%	0.00 [-0.93, 0.93]	2014
ALaCaRT, 2015	2	0.333	234	2	0.5	233	2.5%	0.00 [-0.08, 0.08]	2015
Z6051, 2015	2	2.5	240	3	2	222	2.2%	-1.00 [-1.41, -0.59]	2015
Yao et al, 2018	3.1	1	60	3.9	0.9	60	2.3%	-0.80 [-1.14, -0.46]	2018
Alhanafy et al, 2020	3.95	0.82	60	5.15	0.81	60	2.3%	-1.20 [-1.49, -0.91]	2020
**Subtotal (95% CI)**			**2469**			**1997**	**32.2%**	**-0.83 [-1.06, -0.61]**	
Heterogeneity: Tau² = 0.15; Chi² = 588.28, df = 14 (P < 0.00001); I² = 98%									
Test for overall effect: Z = 7.25 (P < 0.00001)									
**NRCT**									
Schwandner et al, 1999	4.1	1.7	32	5.1	1.5	32	1.6%	-1.00 [-1.79, -0.21]	1999
Wu et al, 2004	1.79	0.21	18	3.25	0.5	18	2.4%	-1.46 [-1.71, -1.21]	2004
Sahakitrungruang et al, 2005	2.45	1	24	2.95	1	25	2.0%	-0.50 [-1.06, 0.06]	2005
Morino et al, 2005	2.6	2.667	98	3.9	2	93	1.8%	-1.30 [-1.97, -0.63]	2005
Law et al, 2006	3	1.167	98	4	4.833	167	1.7%	-1.00 [-1.77, -0.23]	2006
Veenhof et al, 2007	4	0.5	50	5	0.5	50	2.4%	-1.00 [-1.20, -0.80]	2007
Ding et al, 2009	3	2.7	115	4	1.35	220	2.0%	-1.00 [-1.52, -0.48]	2009
Koulas et al, 2009	2.9	0.875	57	3.8	1	60	2.3%	-0.90 [-1.24, -0.56]	2009
Khaikin et al, 2009	3	1.5	32	4	2	50	1.7%	-1.00 [-1.76, -0.24]	2009
Gouvas et al, 2009	2.7	2	45	7.3	3.7	43	1.1%	-4.60 [-5.85, -3.35]	2009
Denoya et al, 2010	1.9	1	32	3.3	2.4	32	1.5%	-1.40 [-2.30, -0.50]	2010
Jun et al, 2011	3	1.1	123	4.4	3	88	1.8%	-1.40 [-2.06, -0.74]	2011
Park et al, 2011	2.6	0.9	130	3.2	1.6	80	2.2%	-0.60 [-0.98, -0.22]	2011
Baik et al, 2011	3.6	1.8	54	4.7	2.5	108	1.8%	-1.10 [-1.77, -0.43]	2011
da Luz Moreira et al, 2011	3	1.667	91	4	1.833	91	2.1%	-1.00 [-1.51, -0.49]	2011
Siani et al, 2012	2.3	0.8	30	4.6	1.9	30	1.7%	-2.30 [-3.04, -1.56]	2012
Li et al, 2012	2.5	1.8	381	3.7	2.9	276	2.2%	-1.20 [-1.59, -0.81]	2012
Seshadri et al, 2012	2	0.5	72	3	0.833	72	2.4%	-1.00 [-1.22, -0.78]	2012
Kellokumpu et al, 2012	2	0.333	100	2	0.167	91	2.5%	0.00 [-0.07, 0.07]	2012
Lee et al, 2013	2.44	0.94	80	3.54	1.49	80	2.2%	-1.10 [-1.49, -0.71]	2013
Yang et al, 2013	2.1	1.5	87	3.5	1.6	90	2.1%	-1.40 [-1.86, -0.94]	2013
Kuo et al, 2013	3.4	2.5	28	3.2	1.5	30	1.2%	0.20 [-0.87, 1.27]	2013
Zhou et al, 2014	3	1.75	57	4	2	65	1.8%	-1.00 [-1.67, -0.33]	2014
Wang et al, 2014	2.8	1.5	100	3.9	2.1	136	2.1%	-1.10 [-1.56, -0.64]	2014
Wang et al, 2015	2.39	0.33	106	2.65	0.38	106	2.5%	-0.26 [-0.36, -0.16]	2015
Zhou et al, 2015	2.8	1	152	3.7	1.1	254	2.4%	-0.90 [-1.11, -0.69]	2015
Huang et al, 2015	1.91	0.89	407	2.41	11.13	326	1.1%	-0.50 [-1.71, 0.71]	2015
Keskin et al, 2016	1.9	1.2	437	2.6	1.4	150	2.4%	-0.70 [-0.95, -0.45]	2016
Kim et al, 2016	1.9	0.9	486	2.5	1	1095	2.5%	-0.60 [-0.70, -0.50]	2016
Chen W et al, 2016	3	0.6	75	3.3	0.8	97	2.4%	-0.30 [-0.51, -0.09]	2016
Wu et al, 2017	3.84	0.82	112	4.24	1.12	121	2.4%	-0.40 [-0.65, -0.15]	2017
De'Angelis et al, 2017	3.13	2.75	52	4.97	4.82	52	0.8%	-1.84 [-3.35, -0.33]	2017
Wu et al, 2018	4	0.167	277	4	0.167	614	2.5%	0.00 [-0.02, 0.02]	2018
Zhang X et al, 2019	3.3	1.117	86	4	1.575	39	2.0%	-0.70 [-1.25, -0.15]	2019
**Subtotal (95% CI)**			**4124**			**4881**	**67.8%**	**-0.90 [-1.08, -0.73]**	
Heterogeneity: Tau² = 0.20; Chi² = 844.17, df = 33 (P < 0.00001); I² = 96%									
Test for overall effect: Z = 10.03 (P < 0.00001)									
**Total (95% CI)**			**6593**			**6878**	**100.0%**	**-0.89 [-1.06, -0.73]**	
Heterogeneity: Tau² = 0.29; Chi² = 3123.46, df = 48 (P < 0.00001); I² = 98%									
Test for overall effect: Z = 10.43 (P < 0.00001)									
Test for subgroup differences: Chi² = 0.25, df = 1 (P = 0.62), I² = 0%									

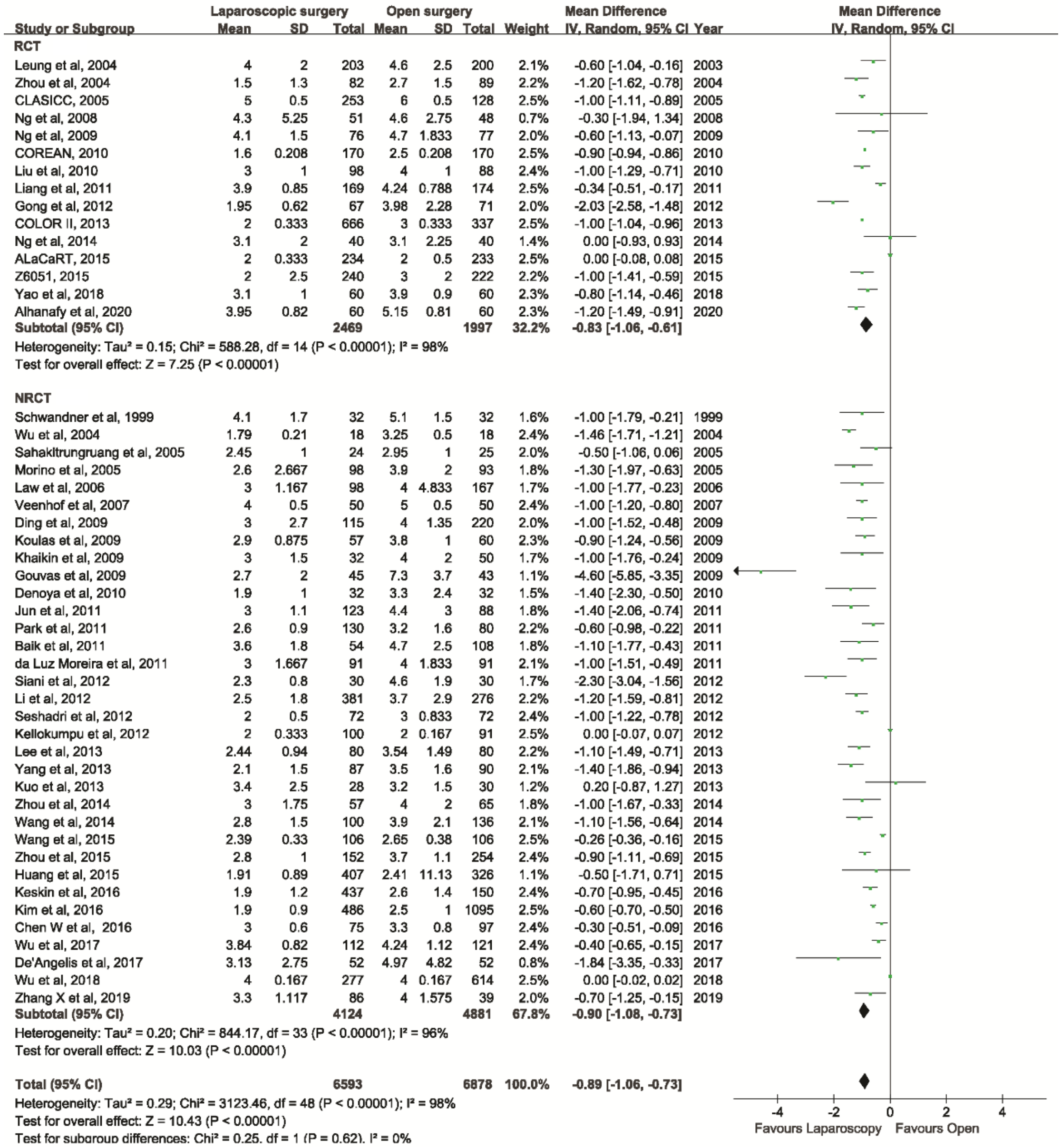

FIGURE S9 Forest plot for days of postoperative analgesic need

Study or Subgroup	Laparoscopic surgery Mean	SD	Total	Open surgery Mean	SD	Total	Weight	Mean Difference IV, Random, 95% CI	Year
**RCT**									
Leung et al, 2004	4	2	203	4.6	2.5	200	2.1%	-0.60 [-1.04, -0.16]	2003
Zhou et al, 2004	1.5	1.3	82	2.7	1.5	89	2.2%	-1.20 [-1.62, -0.78]	2004
CLASICC, 2005	5	0.5	253	6	0.5	128	2.5%	-1.00 [-1.11, -0.89]	2005
Ng et al, 2008	4.3	5.25	51	4.6	2.75	48	0.7%	-0.30 [-1.94, 1.34]	2008
Ng et al, 2009	4.1	1.5	76	4.7	1.833	77	2.0%	-0.60 [-1.13, -0.07]	2009
COREAN, 2010	1.6	0.208	170	2.5	0.208	170	2.5%	-0.90 [-0.94, -0.86]	2010
Liu et al, 2010	3	1	98	4	1	88	2.4%	-1.00 [-1.29, -0.71]	2010
Liang et al, 2011	3.9	0.85	169	4.24	0.788	174	2.5%	-0.34 [-0.51, -0.17]	2011
Gong et al, 2012	1.95	0.62	67	3.98	2.28	71	2.0%	-2.03 [-2.58, -1.48]	2012
COLOR II, 2013	2	0.333	666	3	0.333	337	2.5%	-1.00 [-1.04, -0.96]	2013
Ng et al, 2014	3.1	2	40	3.1	2.25	40	1.4%	0.00 [-0.93, 0.93]	2014
ALaCaRT, 2015	2	0.333	234	2	0.5	233	2.5%	0.00 [-0.08, 0.08]	2015
Z6051, 2015	2	2.5	240	3	2	222	2.2%	-1.00 [-1.41, -0.59]	2015
Yao et al, 2018	3.1	1	60	3.9	0.9	60	2.3%	-0.80 [-1.14, -0.46]	2018
Alhanafy et al, 2020	3.95	0.82	60	5.15	0.81	60	2.3%	-1.20 [-1.49, -0.91]	2020
**Subtotal (95% CI)**			**2469**			**1997**	**32.2%**	**-0.83 [-1.06, -0.61]**	
Heterogeneity: $Tau^2 = 0.15$; $Chi^2 = 588.28$, df = 14 ($P < 0.00001$); $I^2 = 98\%$									
Test for overall effect: Z = 7.25 ($P < 0.00001$)									
**NRCT**									
Schwandner et al, 1999	4.1	1.7	32	5.1	1.5	32	1.6%	-1.00 [-1.79, -0.21]	1999
Wu et al, 2004	1.79	0.21	18	3.25	0.5	18	2.4%	-1.46 [-1.71, -1.21]	2004
Sahakitrungruang et al, 2005	2.45	1	24	2.95	1	25	2.0%	-0.50 [-1.06, 0.06]	2005
Morino et al, 2005	2.6	2.667	98	3.9	2	93	1.8%	-1.30 [-1.97, -0.63]	2005
Law et al, 2006	3	1.167	98	4	4.833	167	1.7%	-1.00 [-1.77, -0.23]	2006
Veenhof et al, 2007	4	0.5	50	5	0.5	50	2.4%	-1.00 [-1.20, -0.80]	2007
Ding et al, 2009	3	2.7	115	4	1.35	220	2.0%	-1.00 [-1.52, -0.48]	2009
Koulas et al, 2009	2.9	0.875	57	3.8	1	60	2.3%	-0.90 [-1.24, -0.56]	2009
Khaikin et al, 2009	3	1.5	32	4	2	50	1.7%	-1.00 [-1.76, -0.24]	2009
Gouvas et al, 2009	2.7	2	45	7.3	3.7	43	1.1%	-4.60 [-5.85, -3.35]	2009
Denoya et al, 2010	1.9	1	32	3.3	2.4	32	1.5%	-1.40 [-2.30, -0.50]	2010
Jun et al, 2011	3	1.1	123	4.4	3	88	1.8%	-1.40 [-2.06, -0.74]	2011
Park et al, 2011	2.6	0.9	130	3.2	1.6	80	2.2%	-0.60 [-0.98, -0.22]	2011
Baik et al, 2011	3.6	1.8	54	4.7	2.5	108	1.8%	-1.10 [-1.77, -0.43]	2011
da Luz Moreira et al, 2011	3	1.667	91	4	1.833	91	2.1%	-1.00 [-1.51, -0.49]	2011
Siani et al, 2012	2.3	0.8	30	4.6	1.9	30	1.7%	-2.30 [-3.04, -1.56]	2012
Li et al, 2012	2.5	1.8	381	3.7	2.9	276	2.2%	-1.20 [-1.59, -0.81]	2012
Seshadri et al, 2012	2	0.5	72	3	0.833	72	2.4%	-1.00 [-1.22, -0.78]	2012
Kellokumpu et al, 2012	2	0.333	100	2	0.167	91	2.5%	0.00 [-0.07, 0.07]	2012
Lee et al, 2013	2.44	0.94	80	3.54	1.49	80	2.2%	-1.10 [-1.49, -0.71]	2013
Yang et al, 2013	2.1	1.5	87	3.5	1.6	90	2.1%	-1.40 [-1.86, -0.94]	2013
Kuo et al, 2013	3.4	2.5	28	3.2	1.5	30	1.2%	0.20 [-0.87, 1.27]	2013
Zhou et al, 2014	3	1.75	57	4	2	65	1.8%	-1.00 [-1.67, -0.33]	2014
Wang et al, 2014	2.8	1.5	100	3.9	2.1	136	2.1%	-1.10 [-1.56, -0.64]	2014
Wang et al, 2015	2.39	0.33	106	2.65	0.38	106	2.5%	-0.26 [-0.36, -0.16]	2015
Zhou et al, 2015	2.8	1	152	3.7	1.1	254	2.4%	-0.90 [-1.11, -0.69]	2015
Huang et al, 2015	1.91	0.89	407	2.41	11.13	326	1.1%	-0.50 [-1.71, 0.71]	2015
Keskin et al, 2016	1.9	1.2	437	2.6	1.4	150	2.4%	-0.70 [-0.95, -0.45]	2016
Kim et al, 2016	1.9	0.9	486	2.5	1	1095	2.5%	-0.60 [-0.70, -0.50]	2016
Chen W et al, 2016	3	0.6	75	3.3	0.8	97	2.4%	-0.30 [-0.51, -0.09]	2016
Wu et al, 2017	3.84	0.82	112	4.24	1.12	121	2.4%	-0.40 [-0.65, -0.15]	2017
De'Angelis et al, 2017	3.13	2.75	52	4.97	4.82	52	0.8%	-1.84 [-3.35, -0.33]	2017
Wu et al, 2018	4	0.167	277	4	0.167	614	2.5%	0.00 [-0.02, 0.02]	2018
Zhang X et al, 2019	3.3	1.117	86	4	1.575	39	2.0%	-0.70 [-1.25, -0.15]	2019
**Subtotal (95% CI)**			**4124**			**4881**	**67.8%**	**-0.90 [-1.08, -0.73]**	
Heterogeneity: $Tau^2 = 0.20$; $Chi^2 = 844.17$, df = 33 ($P < 0.00001$); $I^2 = 96\%$									
Test for overall effect: Z = 10.03 ($P < 0.00001$)									
**Total (95% CI)**			**6593**			**6878**	**100.0%**	**-0.89 [-1.06, -0.73]**	
Heterogeneity: $Tau^2 = 0.29$; $Chi^2 = 3123.46$, df = 48 ($P < 0.00001$); $I^2 = 98\%$									
Test for overall effect: Z = 10.43 ($P < 0.00001$)									
Test for subgroup differences: $Chi^2 = 0.25$, df = 1 ($P = 0.62$), $I^2 = 0\%$									

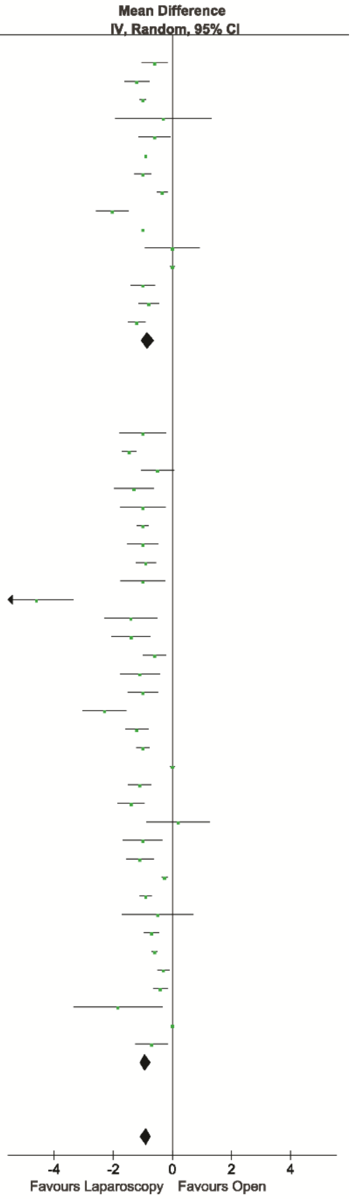

FIGURE S10 Forest plot for days of ambulation

Study or Subgroup	Laparoscopic surgery Mean	SD	Total	Open surgery Mean	SD	Total	Weight	Mean Difference IV, Random, 95% CI	Year
**RCT**									
Leung et al, 2004	3.5	2.333	203	4.5	2.167	200	9.1%	-1.00 [-1.44, -0.56]	2003
Ng et al, 2008	4.4	2.25	51	5.9	4.5	48	1.8%	-1.50 [-2.91, -0.09]	2008
Ng et al, 2009	4	2.167	76	4.7	2	77	5.8%	-0.70 [-1.36, -0.04]	2009
Liang et al, 2011	3.5	0.832	169	4.13	0.983	174	14.3%	-0.63 [-0.82, -0.44]	2011
Ng et al, 2014	4.7	2.5	40	6.2	3.25	40	2.1%	-1.50 [-2.77, -0.23]	2014
Alhanafy et al, 2020	1.35	0.58	60	1.9	0.71	60	13.5%	-0.55 [-0.78, -0.32]	2020
**Subtotal (95% CI)**			**599**			**599**	**46.6%**	**-0.69 [-0.87, -0.51]**	
Heterogeneity: Tau² = 0.01; Chi² = 6.30, df = 5 (P = 0.28); I² = 21%									
Test for overall effect: Z = 7.57 (P < 0.00001)									
**NRCT**									
Sahakitrungruang et al, 2005	2.7	1	24	3.55	1.5	25	5.3%	-0.85 [-1.56, -0.14]	2005
Morino et al, 2005	1.7	0.5	98	3.3	2.333	93	8.3%	-1.60 [-2.08, -1.12]	2005
Ding et al, 2009	7	6.75	115	8	5.4	220	1.7%	-1.00 [-2.43, 0.43]	2009
Seshadri et al, 2012	1	0.333	72	2	0.5	72	15.2%	-1.00 [-1.14, -0.86]	2012
Wang et al, 2014	3	1.2	100	4.3	2.6	136	8.1%	-1.30 [-1.80, -0.80]	2014
Huang et al, 2015	2.8	1.26	407	3.38	1.07	326	14.7%	-0.58 [-0.75, -0.41]	2015
**Subtotal (95% CI)**			**816**			**872**	**53.4%**	**-1.03 [-1.34, -0.71]**	
Heterogeneity: Tau² = 0.10; Chi² = 26.28, df = 5 (P < 0.0001); I² = 81%									
Test for overall effect: Z = 6.32 (P < 0.00001)									
**Total (95% CI)**			**1415**			**1471**	**100.0%**	**-0.89 [-1.09, -0.69]**	
Heterogeneity: Tau² = 0.06; Chi² = 39.42, df = 11 (P < 0.0001); I² = 72%									
Test for overall effect: Z = 8.81 (P < 0.00001)									
Test for subgroup differences: Chi² = 3.30, df = 1 (P = 0.07), I² = 69.7%									

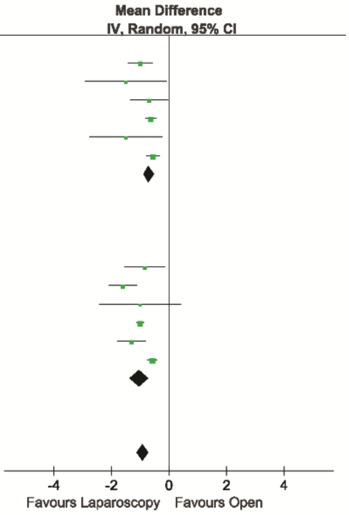

FIGURE S11 Forest plot for length of hospital stay

	Laparoscopic surgery			Open surgery				Std. Mean Difference	
Study or Subgroup	Mean	SD	Total	Mean	SD	Total	Weight	IV, Random, 95% CI	Year
**RCT**									
Leung et al, 2004	8.2	16.167	203	8.7	6	200	1.2%	-0.04 [-0.24, 0.15]	2003
Zhou et al, 2004	8.1	3.1	82	13.3	3.4	89	1.1%	-1.59 [-1.93, -1.24]	2004
CLASICC, 2005	11	1	253	13	1.5	128	1.1%	-1.68 [-1.92, -1.43]	2005
Braga et al, 2007	10	4.9	83	13.6	10	85	1.1%	-0.45 [-0.76, -0.15]	2007
Ng et al, 2008	10.8	5.5	51	11.5	8.25	48	1.1%	-0.10 [-0.49, 0.29]	2008
Lujan et al, 2009	8.2	7.3	101	9.9	6.8	103	1.1%	-0.24 [-0.52, 0.04]	2009
Ng et al, 2009	8.4	5	76	10	6	77	1.1%	-0.29 [-0.61, 0.03]	2009
COREAN, 2010	8	0.833	170	9	0.667	170	1.1%	-1.32 [-1.56, -1.09]	2010
Liu et al, 2010	12	2	98	15	3	88	1.1%	-1.18 [-1.50, -0.87]	2010
Gong et al, 2012	10.4	4.3	67	13.8	5.9	71	1.1%	-0.65 [-0.99, -0.31]	2012
COLOR II, 2013	11.9	11.8	684	12.1	10.6	337	1.2%	-0.02 [-0.15, 0.11]	2013
Ng et al, 2014	10.5	8	40	15	40.25	40	1.1%	-0.15 [-0.59, 0.29]	2014
ALaCaRT, 2015	8	1	238	8	1	235	1.2%	0.00 [-0.18, 0.18]	2015
Z6051, 2015	7.3	5.4	240	7	3.4	222	1.2%	0.07 [-0.12, 0.25]	2015
Yao et al, 2018	11.8	3.2	60	14.9	4.1	60	1.1%	-0.84 [-1.21, -0.46]	2018
Alhanafy et al, 2020	7.15	2.43	60	11.1	2.46	60	1.1%	-1.61 [-2.02, -1.19]	2020
**Subtotal (95% CI)**			**2506**			**2013**	**18.0%**	**-0.63 [-0.94, -0.31]**	
Heterogeneity: Tau² = 0.39; Chi² = 369.08, df = 15 (P < 0.00001); I² = 96%									
Test for overall effect: Z = 3.90 (P < 0.0001)									
**NRCT**									
Schwandner et al, 1999	15.3	6.1	32	21.9	18.9	32	1.0%	-0.46 [-0.96, 0.03]	1999
Leung et al, 2000	26.25	14.75	25	25.5	14	34	1.0%	0.05 [-0.46, 0.57]	2000
Hartley et al, 2001	17.1827	13.3168	21	18.6784	13.2742	22	1.0%	-0.11 [-0.71, 0.49]	2001
Anthuber et al, 2003	14.4	10.1	101	19.9	15.7	334	1.1%	-0.38 [-0.60, -0.15]	2003
Wu et al, 2004	7.8	1.5	18	9.1	3.3	18	1.0%	-0.50 [-1.16, 0.17]	2004
Sahakitrungruang et al, 2005	15	6	24	16.2	6.25	25	1.0%	-0.19 [-0.75, 0.37]	2005
Morino et al, 2005	11.4	9.5	98	13	3.667	93	1.1%	-0.22 [-0.50, 0.07]	2005
Breukink et al, 2005	12	11	41	19	4.25	41	1.1%	-0.83 [-1.28, -0.38]	2005
Wong et al, 2006	15	10.333	71	15	9.5	31	1.1%	0.00 [-0.42, 0.42]	2006
Law et al, 2006	7	5	98	8	9.167	167	1.1%	-0.13 [-0.38, 0.12]	2006
Veenhof et al, 2007	10.5	1.5	50	12	1.5	50	1.1%	-0.99 [-1.41, -0.58]	2007
Lelong et al, 2007	12.6	9.6	104	16	6.9	68	1.1%	-0.39 [-0.70, -0.08]	2007
Ströhlein et al, 2008	15.1	8.6	114	18.7	15	275	1.1%	-0.27 [-0.49, -0.05]	2008
Law et al, 2009	7	0.833	111	9	0.833	310	1.1%	-2.40 [-2.67, -2.13]	2009
Koulas et al, 2009	8	1.5	57	11	2	60	1.1%	-1.68 [-2.10, -1.26]	2009
Ding et al, 2009	12	14.85	115	14	6.667	220	1.1%	-0.19 [-0.42, 0.03]	2009
González et al, 2009	5.5	2	28	7.9	2.25	28	1.0%	-1.11 [-1.68, -0.55]	2009
Laurent et al, 2009	9	14.667	238	16	10.5	233	1.2%	-0.55 [-0.73, -0.36]	2009
Khaikin et al, 2009	6	4	32	7	3.5	50	1.1%	-0.27 [-0.71, 0.18]	2009
Gouvas et al, 2009	5.6	2.2	45	9.4	3.8	43	1.1%	-1.22 [-1.68, -0.76]	2009
Feng et al, 2010	16	6	87	23	11	86	1.1%	-0.79 [-1.10, -0.48]	2010
Denoya et al, 2010	6.1	2.4	32	7.6	2.3	32	1.0%	-0.63 [-1.13, -0.13]	2010
Biondo et al, 2010	7	11	209	10	29.833	395	1.2%	-0.12 [-0.29, 0.05]	2010
Baik et al, 2011	7	3.8	54	8.8	8.7	108	1.1%	-0.24 [-0.57, 0.09]	2011
Jun et al, 2011	9.8	3.8	123	12.8	7.1	88	1.1%	-0.55 [-0.83, -0.27]	2011
Yamamoto et al, 2011	8.25	0.75	22	19.5	7.5	22	0.9%	-2.07 [-2.82, -1.33]	2011
Greenblatt et al, 2011	5	0.667	1040	7	0.833	4380	1.2%	-2.49 [-2.57, -2.41]	2011
Li et al, 2011	9	7.5	113	10	8.5	123	1.1%	-0.12 [-0.38, 0.13]	2011
da Luz Moreira et al, 2011	5	5.333	91	7	15.667	91	1.1%	-0.17 [-0.46, 0.12]	2011
Siani et al, 2012	11.3	1.8	30	15.8	4.3	30	1.0%	-1.35 [-1.91, -0.78]	2012
Kellokumpu et al, 2012	7	0.667	100	9	0.667	91	1.1%	-2.99 [-3.40, -2.57]	2012
Mckay et al, 2012	9	14.6	157	10	11.3	388	1.2%	-0.08 [-0.27, 0.10]	2012
Laurent et al, 2012	9	8.833	110	16	17	65	1.1%	-0.56 [-0.87, -0.25]	2012
Seshadri et al, 2012	12	6.5	72	15	6.667	72	1.1%	-0.45 [-0.78, -0.12]	2012
Jefferies et al, 2012	7.5	2.5	16	19.75	7.75	25	0.9%	-1.91 [-2.68, -1.15]	2012
Lujan et al, 2013	8	1.167	1387	11	1.5	3018	1.2%	-2.14 [-2.21, -2.06]	2013
Park et al, 2013	8.9	10.5	406	12.6	11.8	406	1.2%	-0.33 [-0.47, -0.19]	2013
Yang et al, 2013	5.2	1.8	87	7	2.1	90	1.1%	-0.92 [-1.23, -0.61]	2013
Penninckx et al, 2013	14.6	13.6	764	15.7	12.1	1896	1.2%	-0.09 [-0.17, -0.00]	2013
Lee et al, 2013	8.7049	3.0198	80	11	3.0198	80	1.1%	-0.76 [-1.08, -0.44]	2013
Kuo et al, 2013	15.1	12.5	28	11.7	5	30	1.0%	0.36 [-0.16, 0.88]	2013
Zhou et al, 2014	7	4.25	57	8	6.25	65	1.1%	-0.18 [-0.54, 0.17]	2014
Inada et al, 2014	9.5	1.936	14	22.5	10.508	14	0.8%	-1.67 [-2.55, -0.79]	2014
Wang et al, 2014	7.5	2.4	100	10.8	5.2	136	1.1%	-0.77 [-1.04, -0.51]	2014
Keller et al, 2014	4.8	3.4	62	8.3	6.1	19	1.0%	-0.83 [-1.36, -0.30]	2014
Inomata et al, 2014	17	20.75	38	18	11.75	27	1.0%	-0.06 [-0.55, 0.44]	2014
Kusano et al, 2014	38.5	19.5	19	38.5	16.292	14	0.9%	0.00 [-0.69, 0.69]	2014
Chi et al, 2015	8.4	3.7	89	10.4	5.8	48	1.1%	-0.44 [-0.79, -0.08]	2015
Huang et al, 2015	21.5	4.991	407	25.81	7.868	326	1.2%	-0.67 [-0.82, -0.52]	2015
Li et al, 2015	9	7	129	10	7.333	152	1.1%	-0.14 [-0.37, 0.10]	2015
Zhou et al, 2015	11.7	3.7	152	13.7	4.3	254	1.2%	-0.49 [-0.69, -0.28]	2015
Nussbaum et al, 2015	5	0.5	6430	6	0.667	6430	1.2%	-1.70 [-1.74, -1.66]	2015
Guo et al, 2015	7	2.5	191	8	2.667	191	1.2%	-0.39 [-0.59, -0.18]	2015
Zeng et al, 2015	8	5.667	112	11	9.833	182	1.1%	-0.35 [-0.59, -0.12]	2015
Wang et al, 2015	11.15	4.72	106	12.63	3.96	106	1.1%	-0.34 [-0.61, -0.07]	2015
Keskin et al, 2016	8.9	3.3	437	10.4	5.6	150	1.2%	-0.37 [-0.56, -0.19]	2016
Odermatt et al, 2016	10	10	48	14	30	48	1.1%	-0.18 [-0.58, 0.22]	2016
Chen W et al, 2016	11.7	4.5	75	12.5	4.6	97	1.1%	-0.17 [-0.48, 0.13]	2016
Kim et al, 2016	6.2	1.8	486	7.6	3.5	1095	1.2%	-0.45 [-0.56, -0.35]	2016
Chen Z et al, 2016	14.5	9.75	37	19.9	21	37	1.1%	-0.33 [-0.79, 0.13]	2016
Silva-Velazco et al, 2017	6	5	118	8	12.833	304	1.1%	-0.18 [-0.39, 0.03]	2017
Wu et al, 2017	15.33	6.68	112	15.87	7.09	121	1.1%	-0.08 [-0.34, 0.18]	2017
De'Angelis et al, 2017	15.49	17.17	52	17.96	13.82	52	1.1%	-0.16 [-0.54, 0.23]	2017
Matsuhashi et al, 2017	17.3	5.75	19	20.35	4.716	6	0.8%	-0.53 [-1.46, 0.40]	2017
Garfinkle et al, 2018	5	1.5	34	10	1.5	201	1.1%	-3.32 [-3.80, -2.85]	2018
Tayar et al, 2018	8.3	5.1	50	9.2	4.5	50	1.1%	-0.19 [-0.58, 0.21]	2018
Hida et al, 2018	19	2.333	482	19	2.167	482	1.2%	0.00 [-0.13, 0.13]	2018
Aydın et al, 2018	5.8	1.7	71	7.2	4.3	50	1.1%	-0.46 [-0.82, -0.09]	2018
Chen et al, 2018	4.858	2.573	5578	6.481	3.38	102858	1.2%	-0.49 [-0.51, -0.46]	2018
Wu et al, 2018	9	0.5	277	9	0.667	614	1.2%	0.00 [-0.14, 0.14]	2018
Davis et al, 2019	6.5	7.1	12335	8.9	8.5	19092	1.2%	-0.30 [-0.32, -0.28]	2019
Manchon-Walsh et al, 2019	11.4	13.8	842	13.3	11.6	517	1.2%	-0.15 [-0.26, -0.04]	2019
Zhang X et al, 2019	9	7	86	9	2.75	39	1.1%	0.00 [-0.38, 0.38]	2019
Chen et al, 2019	9.6	4.6	64	9.4	2.4	23	1.1%	0.05 [-0.43, 0.52]	2019
Mar et al, 2020	10	1	400	12	1.833	201	1.2%	-1.49 [-1.68, -1.30]	2020
**Subtotal (95% CI)**			**35940**			**147651**	**82.0%**	**-0.62 [-0.78, -0.45]**	
Heterogeneity: Tau² = 0.50; Chi² = 8343.68, df = 74 (P < 0.00001); I² = 99%									
Test for overall effect: Z = 7.29 (P < 0.00001)									
**Total (95% CI)**			**38446**			**149664**	**100.0%**	**-0.62 [-0.77, -0.47]**	
Heterogeneity: Tau² = 0.49; Chi² = 8760.17, df = 90 (P < 0.00001); I² = 99%									
Test for overall effect: Z = 8.16 (P < 0.00001)									
Test for subgroup differences: Chi² = 0.00, df = 1 (P = 0.97), I² = 0%									

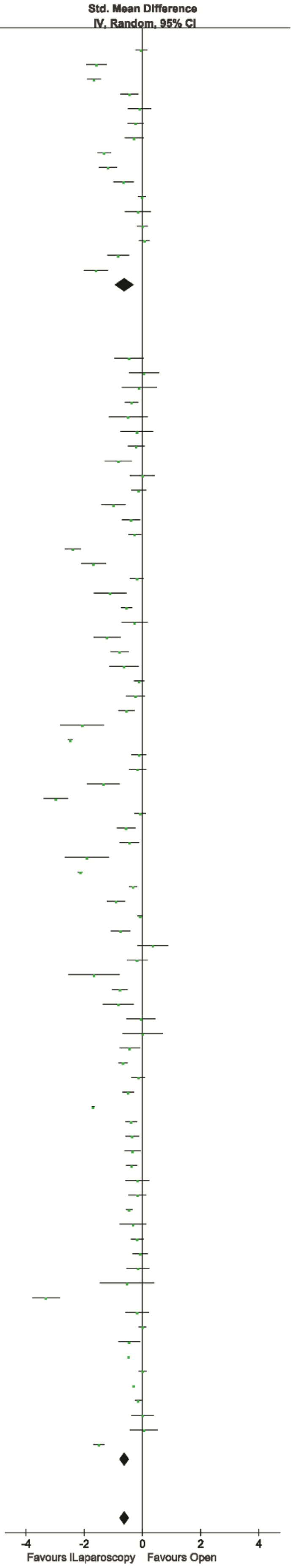

FIGURE S12 Forest plot for overall postoperative complications

Study or Subgroup	Laparoscopic surgery Events	Laparoscopic surgery Total	Open surgery Events	Open surgery Total	Weight	Risk Ratio M-H, Fixed, 95% CI	Year
**RCT**							
Leung et al, 2004	40	203	45	200	0.5%	0.88 [0.60, 1.28]	2003
Araujo et al, 2003	9	13	7	15	0.1%	1.48 [0.77, 2.85]	2003
Zhou et al, 2004	5	82	11	89	0.1%	0.49 [0.18, 1.36]	2004
CLASICC, 2005	101	253	47	128	0.7%	1.09 [0.83, 1.43]	2005
Braga et al, 2007	24	83	34	85	0.4%	0.72 [0.47, 1.11]	2007
Ng et al, 2008	23	51	25	48	0.3%	0.87 [0.58, 1.30]	2008
Ng et al, 2009	23	76	24	77	0.3%	0.97 [0.60, 1.56]	2009
Lujan et al, 2009	34	101	34	103	0.4%	1.02 [0.69, 1.50]	2009
COREAN, 2010	36	170	40	170	0.5%	0.90 [0.61, 1.34]	2010
Liu et al, 2010	5	98	9	88	0.1%	0.50 [0.17, 1.43]	2010
Gong et al, 2012	4	67	6	71	0.1%	0.71 [0.21, 2.39]	2012
COLOR II, 2013	278	697	128	345	2.0%	1.08 [0.91, 1.27]	2013
Ng et al, 2014	13	40	22	40	0.3%	0.59 [0.35, 1.00]	2014
ALaCaRT, 2015	44	238	62	235	0.7%	0.70 [0.50, 0.99]	2015
Z6051, 2015	111	240	112	222	1.3%	0.92 [0.76, 1.11]	2015
Alhanafy et al, 2020	19	60	25	60	0.3%	0.76 [0.47, 1.22]	2020
**Subtotal (95% CI)**		**2472**		**1976**	**7.9%**	**0.92 [0.84, 1.00]**	
Total events	769		631				
Heterogeneity: Chi² = 17.35, df = 15 (P = 0.30); I² = 14%							
Test for overall effect: Z = 1.91 (P = 0.06)							
**NRCT**							
Schwandner et al, 1999	10	32	10	32	0.1%	1.00 [0.48, 2.07]	1999
Leung et al, 2000	12	25	21	34	0.2%	0.78 [0.48, 1.26]	2000
Anthuber et al, 2003	20	101	134	334	0.7%	0.49 [0.33, 0.75]	2003
Wu et al, 2004	4	18	8	18	0.1%	0.50 [0.18, 1.37]	2004
Breukink et al, 2005	15	41	21	41	0.2%	0.71 [0.43, 1.18]	2005
Morino et al, 2005	24	98	22	93	0.3%	1.04 [0.63, 1.71]	2005
Sahakitrungruang et al, 2005	3	24	10	25	0.1%	0.31 [0.10, 1.00]	2005
Law et al, 2006	25	98	49	167	0.4%	0.87 [0.58, 1.31]	2006
Wong et al, 2006	29	71	15	31	0.2%	0.84 [0.53, 1.34]	2006
Lelong et al, 2007	45	104	33	68	0.5%	0.89 [0.64, 1.24]	2007
Veenhof et al, 2007	10	50	16	50	0.2%	0.63 [0.31, 1.24]	2007
Staudacher et al, 2007	32	108	22	79	0.3%	1.06 [0.67, 1.68]	2007
Ströhlein et al, 2008	16	114	59	275	0.4%	0.65 [0.39, 1.09]	2008
Laurent et al, 2009	77	238	88	233	1.0%	0.86 [0.67, 1.10]	2009
Koulas et al, 2009	13	57	15	60	0.2%	0.91 [0.48, 1.74]	2009
Khaikin et al, 2009	25	32	38	50	0.3%	1.03 [0.81, 1.31]	2009
Law et al, 2009	34	111	96	310	0.6%	0.99 [0.71, 1.37]	2009
Ding et al, 2009	18	115	38	220	0.3%	0.91 [0.54, 1.51]	2009
Gouvas et al, 2009	15	45	27	43	0.3%	0.53 [0.33, 0.85]	2009
Biondo et al, 2010	81	209	176	395	1.4%	0.87 [0.71, 1.07]	2010
Fujimoto et al, 2010	3	35	4	19	0.1%	0.41 [0.10, 1.63]	2010
Feng et al, 2010	25	87	33	86	0.4%	0.75 [0.49, 1.15]	2010
Li et al, 2011	21	113	24	123	0.3%	0.95 [0.56, 1.61]	2011
da Luz Moreira et al, 2011	32	91	35	91	0.4%	0.91 [0.62, 1.34]	2011
Jun et al, 2011	15	123	18	88	0.2%	0.60 [0.32, 1.12]	2011
Greenblatt et al, 2011	189	1040	907	4380	4.0%	0.88 [0.76, 1.01]	2011
Baik et al, 2011	12	54	35	185	0.2%	1.17 [0.66, 2.10]	2011
Yamamoto et al, 2011	7	22	13	22	0.1%	0.54 [0.27, 1.09]	2011
Park et al, 2011	17	130	14	80	0.2%	0.75 [0.39, 1.43]	2011
Mckay et al, 2012	65	157	234	388	1.5%	0.69 [0.56, 0.84]	2012
Kellokumpu et al, 2012	31	100	39	91	0.5%	0.72 [0.50, 1.05]	2012
Jefferies et al, 2012	4	16	9	25	0.1%	0.69 [0.26, 1.88]	2012
Seshadri et al, 2012	14	72	16	72	0.2%	0.88 [0.46, 1.66]	2012
Laurent et al, 2012	45	110	35	65	0.5%	0.76 [0.55, 1.04]	2012
Siani et al, 2012	6	30	11	30	0.1%	0.55 [0.23, 1.28]	2012
Park et al, 2013	84	406	93	406	1.1%	0.90 [0.70, 1.17]	2013
Lujan et al, 2013	531	1387	1375	3018	9.9%	0.84 [0.78, 0.91]	2013
Penninckx et al, 2013	241	759	763	1881	5.0%	0.78 [0.70, 0.88]	2013
Yang et al, 2013	2	87	5	90	0.1%	0.41 [0.08, 2.08]	2013
Lee et al, 2013	20	80	25	80	0.3%	0.80 [0.49, 1.32]	2013
Zhou et al, 2014	21	57	29	65	0.3%	0.83 [0.53, 1.28]	2014
Kusano et al, 2014	5	19	5	14	0.1%	0.74 [0.26, 2.06]	2014
Wang et al, 2014	4	100	20	136	0.2%	0.27 [0.10, 0.77]	2014
Inada et al, 2014	7	14	8	14	0.1%	0.88 [0.44, 1.75]	2014
Keller et al, 2014	20	62	7	19	0.1%	0.88 [0.44, 1.75]	2014
Inomata et al, 2014	7	38	6	27	0.1%	0.83 [0.31, 2.19]	2014
Huang et al, 2015	85	492	98	424	1.2%	0.75 [0.58, 0.97]	2015
Zeng et al, 2015	17	112	48	182	0.4%	0.58 [0.35, 0.95]	2015
Wang et al, 2015	9	106	13	106	0.1%	0.69 [0.31, 1.55]	2015
Chi et al, 2015	18	89	13	48	0.2%	0.75 [0.40, 1.39]	2015
Li et al, 2015	27	129	42	152	0.4%	0.76 [0.50, 1.16]	2015
Guo et al, 2015	19	191	23	191	0.3%	0.83 [0.47, 1.47]	2015
Zhou et al, 2015	38	152	66	254	0.6%	0.96 [0.68, 1.36]	2015
Kim et al, 2016	63	486	211	1095	1.5%	0.67 [0.52, 0.87]	2016
Nonaka et al, 2016	4	40	17	38	0.2%	0.22 [0.08, 0.60]	2016
Keskin et al, 2016	132	437	49	150	0.8%	0.92 [0.71, 1.21]	2016
Chen Z et al, 2016	10	37	19	37	0.2%	0.53 [0.28, 0.97]	2016
Chen W et al, 2016	18	75	29	97	0.3%	0.80 [0.48, 1.33]	2016
Wu et al, 2017	44	112	48	121	0.5%	0.99 [0.72, 1.36]	2017
Matsuhashi et al, 2017	3	19	1	6	0.0%	0.95 [0.12, 7.50]	2017
Silva-Velazco et al, 2017	52	118	168	304	1.1%	0.80 [0.64, 1.00]	2017
De'Angelis et al, 2017	16	52	25	52	0.3%	0.64 [0.39, 1.05]	2017
Garfinkle et al, 2018	11	34	102	201	0.3%	0.64 [0.38, 1.06]	2018
Wu et al, 2018	13	277	34	614	0.2%	0.85 [0.45, 1.58]	2018
Chen et al, 2018	1671	5578	37011	102858	43.6%	0.83 [0.80, 0.87]	2018
Hida et al, 2018	146	482	189	482	2.2%	0.77 [0.65, 0.92]	2018
Tayar et al, 2018	34	50	38	50	0.4%	0.89 [0.70, 1.14]	2018
Chen et al, 2019	7	64	2	23	0.0%	1.26 [0.28, 5.62]	2019
Zhang X et al, 2019	9	86	9	39	0.1%	0.45 [0.20, 1.05]	2019
Manchon-Walsh et al, 2019	336	842	218	517	3.1%	0.95 [0.83, 1.08]	2019
**Subtotal (95% CI)**		**16940**		**122094**	**92.1%**	**0.82 [0.80, 0.84]**	
Total events	4718		43134				
Heterogeneity: Chi² = 66.83, df = 69 (P = 0.55); I² = 0%							
Test for overall effect: Z = 13.97 (P < 0.00001)							
**Total (95% CI)**		**19412**		**124070**	**100.0%**	**0.83 [0.81, 0.85]**	
Total events	5487		43765				
Heterogeneity: Chi² = 90.94, df = 85 (P = 0.31); I² = 7%							
Test for overall effect: Z = 13.96 (P < 0.00001)							
Test for subgroup differences: Chi² = 5.85, df = 1 (P = 0.02), I² = 82.9%							

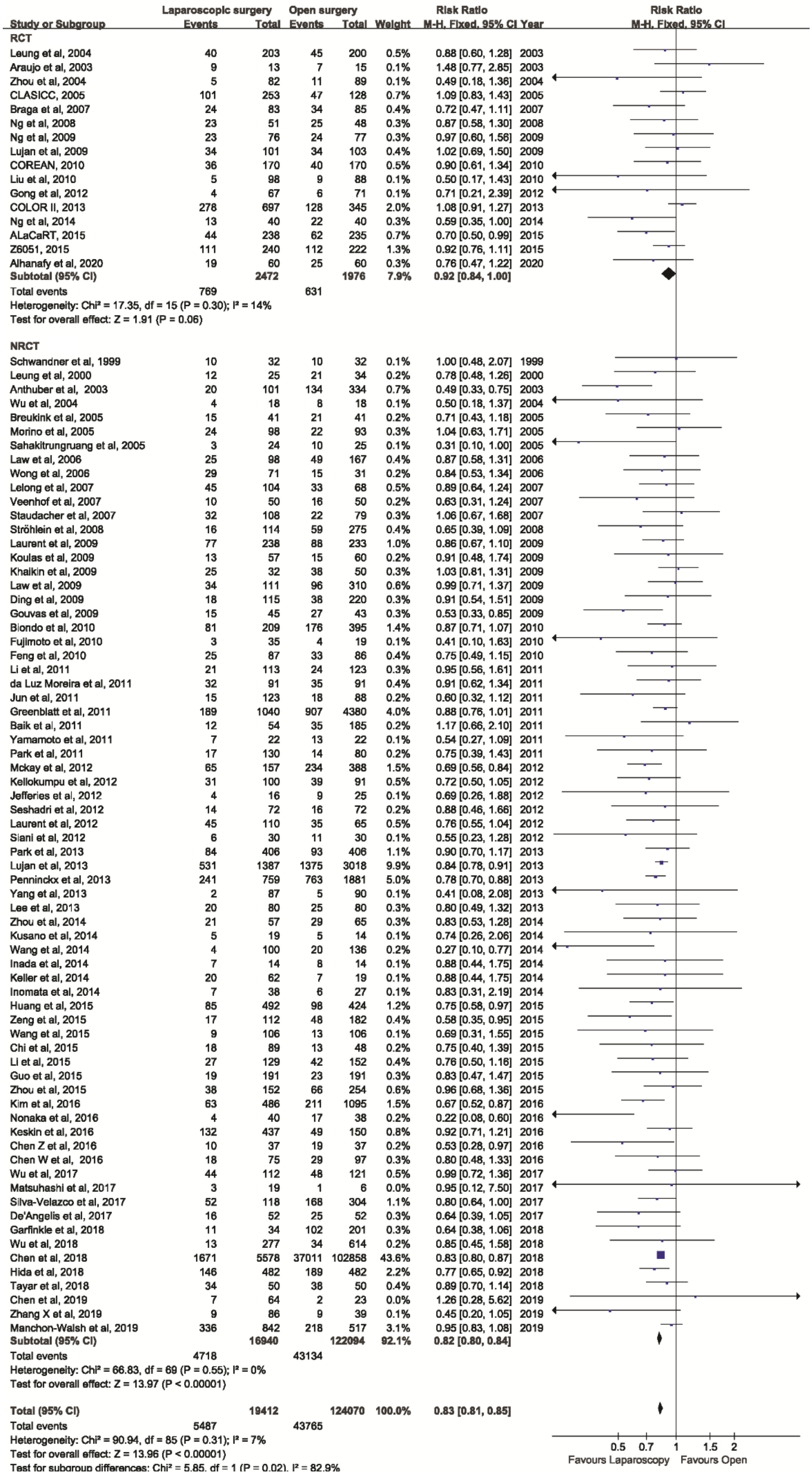

FIGURE S13 Forest plot for hemorrhage

Study or Subgroup	Laparoscopic surgery Events	Laparoscopic surgery Total	Open surgery Events	Open surgery Total	Weight	Risk Ratio M-H, Fixed, 95% CI	Year
**RCT**							
Leung et al, 2004	2	203	4	200	1.4%	0.49 [0.09, 2.66]	2003
Braga et al, 2007	0	83	4	85	1.5%	0.11 [0.01, 2.08]	2007
Ng et al, 2008	0	51	2	48	0.9%	0.19 [0.01, 3.83]	2008
Ng et al, 2009	1	76	3	77	1.0%	0.34 [0.04, 3.18]	2009
Lujan et al, 2009	1	101	1	103	0.3%	1.02 [0.06, 16.08]	2009
COREAN, 2010	1	170	3	170	1.0%	0.33 [0.04, 3.17]	2010
ALaCaRT, 2015	10	238	4	235	1.4%	2.47 [0.79, 7.76]	2015
Yao et al, 2018	3	60	6	60	2.1%	0.50 [0.13, 1.91]	2018
Alhanafy et al, 2020	3	60	4	60	1.4%	0.75 [0.18, 3.21]	2020
**Subtotal (95% CI)**		**1042**		**1038**	**11.1%**	**0.68 [0.40, 1.17]**	
Total events	21		31				
Heterogeneity: Chi² = 8.21, df = 8 (P = 0.41); I² = 3%							
Test for overall effect: Z = 1.39 (P = 0.16)							
**NRCT**							
Schwandner et al, 1999	1	32	1	32	0.3%	1.00 [0.07, 15.30]	1999
Leung et al, 2000	1	25	0	34	0.1%	4.04 [0.17, 95.20]	2000
Wu et al, 2004	0	18	2	18	0.9%	0.20 [0.01, 3.89]	2004
Breukink et al, 2005	2	41	1	41	0.3%	2.00 [0.19, 21.21]	2005
Sahakitrungruang et al, 2005	1	24	0	25	0.2%	3.12 [0.13, 73.04]	2005
Lelong et al, 2007	5	104	4	68	1.7%	0.82 [0.23, 2.94]	2007
Veenhof et al, 2007	0	50	1	50	0.5%	0.33 [0.01, 7.99]	2007
Laurent et al, 2009	6	238	7	233	2.4%	0.84 [0.29, 2.46]	2009
Ding et al, 2009	3	115	2	220	0.5%	2.87 [0.49, 16.93]	2009
Gouvas et al, 2009	2	45	4	43	1.4%	0.48 [0.09, 2.48]	2009
Koulas et al, 2009	2	57	2	60	0.7%	1.05 [0.15, 7.22]	2009
Feng et al, 2010	7	87	5	86	1.7%	1.38 [0.46, 4.19]	2010
da Luz Moreira et al, 2011	1	91	2	91	0.7%	0.50 [0.05, 5.42]	2011
Greenblatt et al, 2011	52	1040	307	4380	40.7%	0.71 [0.54, 0.95]	2011
Baik et al, 2011	0	54	1	108	0.3%	0.66 [0.03, 15.95]	2011
Kellokumpu et al, 2012	2	100	0	91	0.2%	4.55 [0.22, 93.63]	2012
Mckay et al, 2012	10	157	34	388	6.8%	0.73 [0.37, 1.44]	2012
Lujan et al, 2013	12	1387	12	3018	2.6%	2.18 [0.98, 4.83]	2013
Zhou et al, 2014	0	57	1	65	0.5%	0.38 [0.02, 9.13]	2014
Keller et al, 2014	2	62	1	19	0.5%	0.61 [0.06, 6.39]	2014
Wang et al, 2014	0	100	1	136	0.4%	0.45 [0.02, 10.98]	2014
Inada et al, 2014	0	14	2	14	0.9%	0.20 [0.01, 3.82]	2014
Guo et al, 2015	2	191	1	191	0.3%	2.00 [0.18, 21.87]	2015
Zhou et al, 2015	4	152	17	254	4.4%	0.39 [0.13, 1.15]	2015
Chi et al, 2015	1	89	0	48	0.2%	1.63 [0.07, 39.34]	2015
Huang et al, 2015	10	492	6	424	2.2%	1.44 [0.53, 3.92]	2015
Li et al, 2015	5	129	8	152	2.5%	0.74 [0.25, 2.20]	2015
Keskin et al, 2016	1	437	0	150	0.3%	1.03 [0.04, 25.25]	2016
Odermatt et al, 2016	0	48	1	48	0.5%	0.33 [0.01, 7.98]	2016
Chen W et al, 2016	1	75	2	97	0.6%	0.65 [0.06, 7.00]	2016
Liu et al, 2016	2	58	3	39	1.2%	0.45 [0.08, 2.56]	2016
Kim et al, 2016	2	486	17	1095	3.6%	0.27 [0.06, 1.14]	2016
De'Angelis et al, 2017	0	52	3	52	1.2%	0.14 [0.01, 2.70]	2017
Matsuhashi et al, 2017	1	19	0	6	0.3%	1.05 [0.05, 22.91]	2017
Chen et al, 2018	0	5578	91	102858	3.3%	0.10 [0.01, 1.62]	2018
Wu et al, 2018	4	277	10	614	2.1%	0.89 [0.28, 2.80]	2018
Zhang X et al, 2019	0	86	1	39	0.7%	0.15 [0.01, 3.68]	2019
Zhang Z et al, 2019	2	112	3	116	1.0%	0.69 [0.12, 4.05]	2019
**Subtotal (95% CI)**		**12179**		**115403**	**88.9%**	**0.75 [0.62, 0.91]**	
Total events	144		553				
Heterogeneity: Chi² = 27.76, df = 37 (P = 0.86); I² = 0%							
Test for overall effect: Z = 3.00 (P = 0.003)							
**Total (95% CI)**		**13221**		**116441**	**100.0%**	**0.74 [0.62, 0.89]**	
Total events	165		584				
Heterogeneity: Chi² = 36.06, df = 46 (P = 0.85); I² = 0%							
Test for overall effect: Z = 3.28 (P = 0.001)							
Test for subgroup differences: Chi² = 0.10, df = 1 (P = 0.75), I² = 0%							

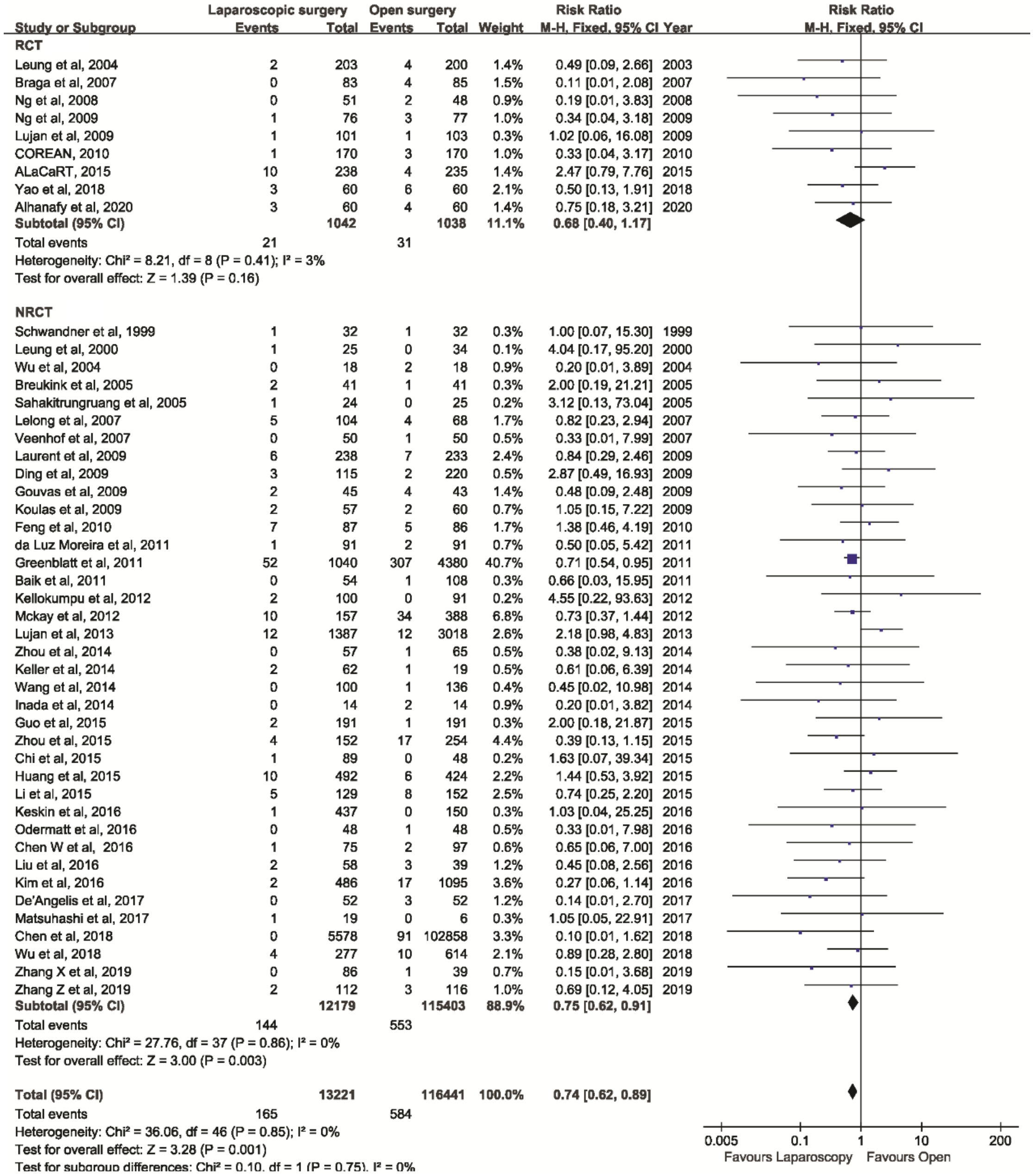

FIGURE S14 Forest plot for wound infection

Study or Subgroup	Laparoscopic surgery Events	Laparoscopic surgery Total	Open surgery Events	Open surgery Total	Weight	Risk Ratio M-H, Fixed, 95% CI	Year
**RCT**							
Leung et al, 2004	1	203	4	200	0.4%	0.25 [0.03, 2.18]	2003
Zhou et al, 2004	1	82	3	89	0.3%	0.36 [0.04, 3.41]	2004
CLASICC, 2005	26	253	9	128	1.2%	1.46 [0.71, 3.03]	2005
Braga et al, 2007	8	83	9	85	0.9%	0.91 [0.37, 2.25]	2007
Ng et al, 2009	1	76	4	77	0.4%	0.25 [0.03, 2.21]	2009
Lujan et al, 2009	5	101	10	103	1.0%	0.51 [0.18, 1.44]	2009
Liu et al, 2010	2	96	3	85	0.3%	0.59 [0.10, 3.45]	2010
COREAN, 2010	2	170	0	170	0.1%	5.00 [0.24, 103.38]	2010
Liang et al, 2011	4	169	6	174	0.6%	0.69 [0.20, 2.39]	2011
Gong et al, 2012	1	67	1	71	0.1%	1.06 [0.07, 16.60]	2012
COLOR II, 2013	58	461	25	240	3.4%	1.21 [0.78, 1.88]	2013
Ng et al, 2014	1	40	2	40	0.2%	0.50 [0.05, 5.30]	2014
ALaCaRT, 2015	7	238	8	235	0.8%	0.86 [0.32, 2.34]	2015
Z6051, 2015	5	240	5	222	0.5%	0.93 [0.27, 3.15]	2015
Yao et al, 2018	1	60	3	60	0.3%	0.33 [0.04, 3.11]	2018
Alhanafy et al, 2020	6	60	5	60	0.5%	1.20 [0.39, 3.72]	2020
**Subtotal (95% CI)**		**2399**		**2039**	**11.1%**	**0.95 [0.73, 1.23]**	
Total events	129		97				
Heterogeneity: Chi² = 10.51, df = 15 (P = 0.79); I² = 0%							
Test for overall effect: Z = 0.41 (P = 0.68)							
**NRCT**							
Schwandner et al, 1999	0	32	1	32	0.2%	0.33 [0.01, 7.89]	1999
Hartley et al, 2001	4	21	1	22	0.1%	4.19 [0.51, 34.50]	2001
Anthuber et al, 2003	9	101	25	334	1.2%	1.19 [0.57, 2.47]	2003
Sahakitrungruang et al, 2005	2	24	2	25	0.2%	1.04 [0.16, 6.81]	2005
Breukink et al, 2005	2	23	4	28	0.4%	0.61 [0.12, 3.03]	2005
Morino et al, 2005	10	98	3	93	0.3%	3.16 [0.90, 11.14]	2005
Law et al, 2006	1	98	5	167	0.4%	0.34 [0.04, 2.88]	2006
Staudacher et al, 2007	16	108	10	79	1.2%	1.17 [0.56, 2.44]	2007
Veenhof et al, 2007	5	50	3	50	0.3%	1.67 [0.42, 6.60]	2007
Lelong et al, 2007	12	104	14	68	1.7%	0.56 [0.28, 1.14]	2007
Ströhlein et al, 2008	9	114	42	275	2.5%	0.52 [0.26, 1.03]	2008
González et al, 2009	2	28	3	28	0.3%	0.67 [0.12, 3.69]	2009
Laurent et al, 2009	12	238	15	233	1.6%	0.78 [0.37, 1.64]	2009
Ding et al, 2009	8	115	9	220	0.6%	1.70 [0.67, 4.29]	2009
Koulas et al, 2009	2	57	3	60	0.3%	0.70 [0.12, 4.05]	2009
Gouvas et al, 2009	7	45	4	43	0.4%	1.67 [0.53, 5.31]	2009
Biondo et al, 2010	14	134	20	249	1.4%	1.30 [0.68, 2.49]	2010
Feng et al, 2010	5	87	3	86	0.3%	1.65 [0.41, 6.68]	2010
Fujimoto et al, 2010	0	35	1	19	0.2%	0.19 [0.01, 4.34]	2010
da Luz Moreira et al, 2011	2	91	5	91	0.5%	0.40 [0.08, 2.01]	2011
Jun et al, 2011	7	123	8	88	1.0%	0.63 [0.24, 1.66]	2011
Park et al, 2011	5	130	5	80	0.6%	0.62 [0.18, 2.06]	2011
Yamamoto et al, 2011	1	22	1	22	0.1%	1.00 [0.07, 15.00]	2011
Li et al, 2011	21	113	24	123	2.4%	0.95 [0.56, 1.61]	2011
Baik et al, 2011	4	39	6	82	0.4%	1.40 [0.42, 4.68]	2011
Mckay et al, 2012	8	157	19	388	1.1%	1.04 [0.47, 2.33]	2012
Li et al, 2012	12	381	35	276	4.2%	0.25 [0.13, 0.47]	2012
Seshadri et al, 2012	3	72	6	72	0.6%	0.50 [0.13, 1.92]	2012
Kellokumpu et al, 2012	11	71	8	70	0.8%	1.36 [0.58, 3.17]	2012
Lee et al, 2013	2	80	0	80	0.1%	5.00 [0.24, 102.53]	2013
Penninckx et al, 2013	44	524	97	1309	5.7%	1.13 [0.81, 1.59]	2013
Yang et al, 2013	1	87	1	90	0.1%	1.03 [0.07, 16.28]	2013
Lujan et al, 2013	81	1387	172	3018	11.1%	1.02 [0.79, 1.32]	2013
Park et al, 2013	37	406	30	406	3.1%	1.23 [0.78, 1.96]	2013
Zhou et al, 2014	1	57	2	65	0.2%	0.57 [0.05, 6.12]	2014
Keller et al, 2014	3	48	1	11	0.2%	0.69 [0.08, 6.00]	2014
Inomata et al, 2014	2	38	0	27	0.1%	3.59 [0.18, 71.91]	2014
Kusano et al, 2014	0	19	1	14	0.2%	0.25 [0.01, 5.72]	2014
Guo et al, 2015	7	191	11	191	1.1%	0.64 [0.25, 1.61]	2015
Chi et al, 2015	3	89	1	48	0.1%	1.62 [0.17, 15.13]	2015
Zhou et al, 2015	6	152	5	254	0.4%	2.01 [0.62, 6.46]	2015
Zeng et al, 2015	2	112	6	182	0.5%	0.54 [0.11, 2.64]	2015
Huang et al, 2015	24	492	15	424	1.7%	1.38 [0.73, 2.59]	2015
Li et al, 2015	8	129	11	152	1.0%	0.86 [0.36, 2.07]	2015
Keskin et al, 2016	28	437	9	150	1.4%	1.07 [0.52, 2.21]	2016
Nonaka et al, 2016	2	40	4	38	0.4%	0.47 [0.09, 2.44]	2016
Liu et al, 2016	3	58	2	39	0.2%	1.01 [0.18, 5.76]	2016
Allaix et al, 2016	10	153	8	154	0.8%	1.26 [0.51, 3.10]	2016
Chen W et al, 2016	1	75	3	97	0.3%	0.43 [0.05, 4.06]	2016
Chen Z et al, 2016	1	37	2	37	0.2%	0.50 [0.05, 5.28]	2016
Kim et al, 2016	21	486	34	1095	2.1%	1.39 [0.82, 2.37]	2016
Silva-Velazco et al, 2017	3	118	10	304	0.6%	0.77 [0.22, 2.76]	2017
De'Angelis et al, 2017	4	52	5	52	0.5%	0.80 [0.23, 2.81]	2017
Wu et al, 2017	3	112	3	121	0.3%	1.08 [0.22, 5.24]	2017
Andersen et al, 2018	269	1896	206	1716	22.2%	1.18 [1.00, 1.40]	2018
Garfinkle et al, 2018	3	34	20	201	0.6%	0.89 [0.28, 2.82]	2018
Wu et al, 2018	11	277	24	614	1.5%	1.02 [0.50, 2.04]	2018
Hida et al, 2018	52	482	57	482	5.9%	0.91 [0.64, 1.30]	2018
Zhang X et al, 2019	1	86	0	39	0.1%	1.38 [0.06, 33.12]	2019
Zhang Z et al, 2019	6	112	8	116	0.8%	0.78 [0.28, 2.17]	2019
**Subtotal (95% CI)**		**10977**		**14929**	**88.9%**	**1.02 [0.93, 1.12]**	
Total events	833		1033				
Heterogeneity: Chi² = 57.82, df = 59 (P = 0.52); I² = 0%							
Test for overall effect: Z = 0.45 (P = 0.65)							
**Total (95% CI)**		**13376**		**16968**	**100.0%**	**1.01 [0.93, 1.10]**	
Total events	962		1130				
Heterogeneity: Chi² = 68.54, df = 75 (P = 0.69); I² = 0%							
Test for overall effect: Z = 0.29 (P = 0.77)							
Test for subgroup differences: Chi² = 0.28, df = 1 (P = 0.60), I² = 0%							

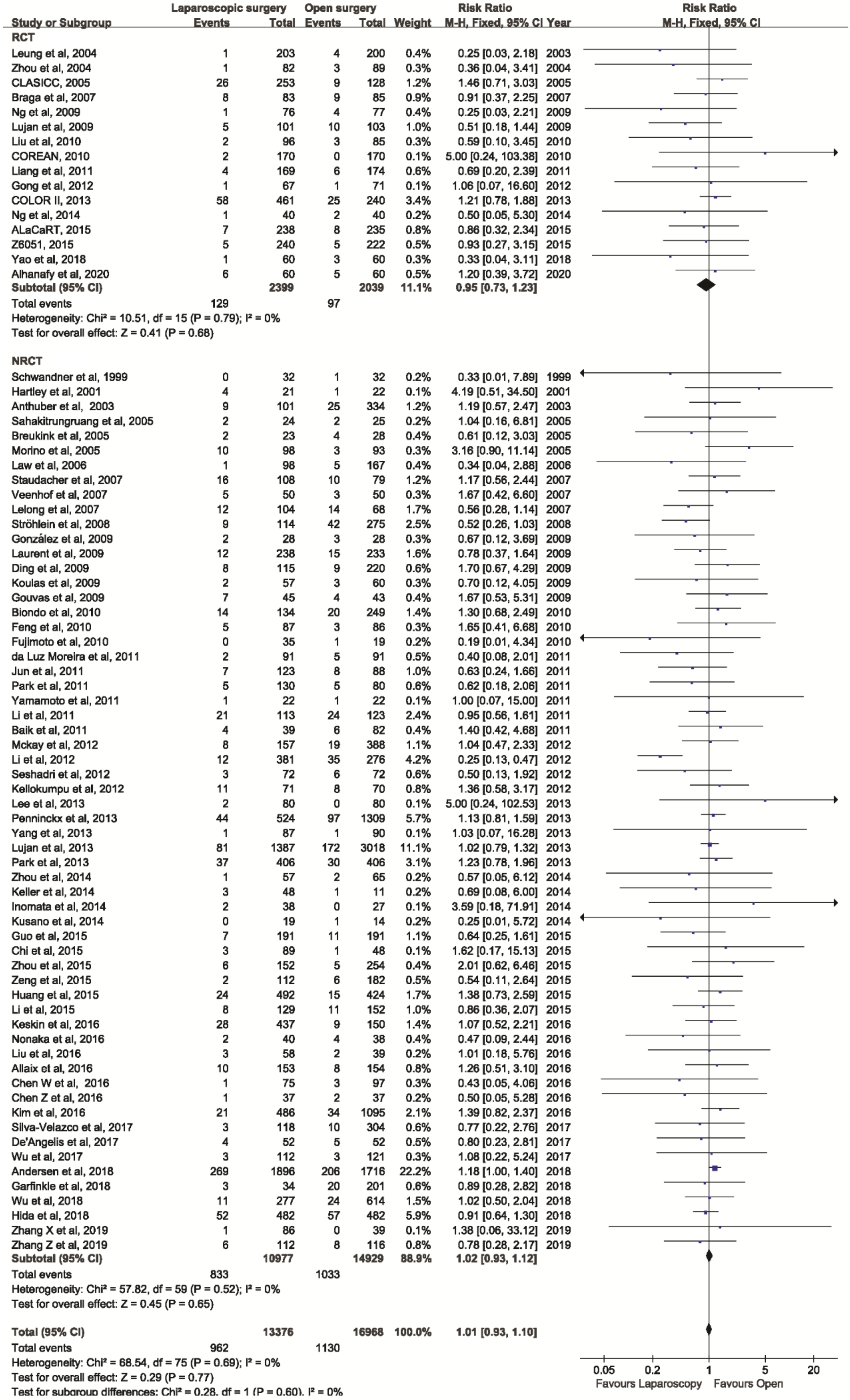

FIGURE S15 Forest plot for ileus

Study or Subgroup	Laparoscopic surgery Events	Laparoscopic surgery Total	Open surgery Events	Open surgery Total	Weight	Risk Ratio M-H, Fixed, 95% CI	Year
**RCT**							
Leung et al, 2004	1	203	2	200	0.2%	0.49 [0.05, 5.39]	2003
Braga et al, 2007	3	83	4	85	0.3%	0.77 [0.18, 3.33]	2007
Ng et al, 2008	1	51	1	48	0.1%	0.94 [0.06, 14.63]	2008
Lujan et al, 2009	3	101	2	103	0.2%	1.53 [0.26, 8.96]	2009
COREAN, 2010	0	170	1	170	0.1%	0.33 [0.01, 8.13]	2010
Liang et al, 2011	2	169	1	174	0.1%	2.06 [0.19, 22.50]	2011
COLOR II, 2013	51	697	22	345	2.3%	1.15 [0.71, 1.86]	2013
Ng et al, 2014	1	40	1	40	0.1%	1.00 [0.06, 15.44]	2014
Alhanafy et al, 2020	4	60	3	60	0.2%	1.33 [0.31, 5.70]	2020
**Subtotal (95% CI)**		**1574**		**1225**	**3.5%**	**1.10 [0.74, 1.63]**	
Total events	66		37				
Heterogeneity: Chi² = 1.71, df = 8 (P = 0.99); I² = 0%							
Test for overall effect: Z = 0.47 (P = 0.64)							
**NRCT**							
Leung et al, 2000	1	25	0	34	0.0%	4.04 [0.17, 95.20]	2000
Breukink et al, 2005	1	41	4	41	0.3%	0.25 [0.03, 2.14]	2005
Staudacher et al, 2007	1	108	0	79	0.0%	2.20 [0.09, 53.35]	2007
Veenhof et al, 2007	0	50	2	50	0.2%	0.20 [0.01, 4.06]	2007
Ströhleln et al, 2008	2	114	2	275	0.1%	2.41 [0.34, 16.92]	2008
Ding et al, 2009	1	115	1	220	0.1%	1.91 [0.12, 30.30]	2009
Laurent et al, 2009	10	238	10	233	0.8%	0.98 [0.42, 2.31]	2009
Feng et al, 2010	2	87	4	86	0.3%	0.49 [0.09, 2.63]	2010
Greenblatt et al, 2011	79	1040	324	4380	9.7%	1.03 [0.81, 1.30]	2011
Park et al, 2011	1	130	1	80	0.1%	0.62 [0.04, 9.70]	2011
Baik et al, 2011	3	54	3	108	0.2%	2.00 [0.42, 9.58]	2011
da Luz Moreira et al, 2011	2	91	4	91	0.3%	0.50 [0.09, 2.66]	2011
Li et al, 2011	2	113	2	123	0.1%	1.09 [0.16, 7.60]	2011
Jun et al, 2011	1	123	2	88	0.2%	0.36 [0.03, 3.88]	2011
Yamamoto et al, 2011	1	22	1	22	0.1%	1.00 [0.07, 15.00]	2011
Kellokumpu et al, 2012	13	71	9	70	0.7%	1.42 [0.65, 3.12]	2012
Laurent et al, 2012	28	110	13	65	1.3%	1.27 [0.71, 2.28]	2012
Mckay et al, 2012	10	157	23	388	1.0%	1.07 [0.52, 2.21]	2012
Lujan et al, 2013	3	1387	19	3018	0.9%	0.34 [0.10, 1.16]	2013
Keller et al, 2014	3	62	0	19	0.1%	2.22 [0.12, 41.21]	2014
Inada et al, 2014	3	14	3	14	0.2%	1.00 [0.24, 4.13]	2014
Inomata et al, 2014	4	38	1	27	0.1%	2.84 [0.34, 24.04]	2014
Kusano et al, 2014	2	19	2	14	0.2%	0.74 [0.12, 4.61]	2014
Huang et al, 2015	1	492	1	424	0.1%	0.86 [0.05, 13.74]	2015
Chi et al, 2015	1	89	1	48	0.1%	0.54 [0.03, 8.43]	2015
Zhou et al, 2015	5	152	2	254	0.1%	4.18 [0.82, 21.27]	2015
Li et al, 2015	2	129	3	152	0.2%	0.79 [0.13, 4.63]	2015
Chen W et al, 2016	1	75	4	97	0.3%	0.32 [0.04, 2.83]	2016
Keskin et al, 2016	5	437	0	150	0.1%	3.79 [0.21, 68.18]	2016
Chen Z et al, 2016	2	37	2	37	0.2%	1.00 [0.15, 6.73]	2016
Liu et al, 2016	4	58	3	39	0.3%	0.90 [0.21, 3.79]	2016
Matsuhashi et al, 2017	0	19	1	6	0.2%	0.12 [0.01, 2.55]	2017
Wu et al, 2017	1	112	0	121	0.0%	3.24 [0.13, 78.70]	2017
Silva-Velazco et al, 2017	5	118	23	308	1.0%	0.57 [0.22, 1.46]	2017
Wu et al, 2018	1	277	16	614	0.8%	0.14 [0.02, 1.04]	2018
Zhang X et al, 2019	1	86	0	39	0.1%	1.38 [0.06, 33.12]	2019
Davis et al, 2019	749	12335	1250	19092	76.3%	0.93 [0.85, 1.01]	2019
**Subtotal (95% CI)**		**18625**		**30906**	**96.5%**	**0.94 [0.87, 1.01]**	
Total events	951		1736				
Heterogeneity: Chi² = 26.69, df = 36 (P = 0.87); I² = 0%							
Test for overall effect: Z = 1.69 (P = 0.09)							
**Total (95% CI)**		**20199**		**32131**	**100.0%**	**0.94 [0.87, 1.02]**	
Total events	1017		1773				
Heterogeneity: Chi² = 28.99, df = 45 (P = 0.97); I² = 0%							
Test for overall effect: Z = 1.57 (P = 0.12)							
Test for subgroup differences: Chi² = 0.61, df = 1 (P = 0.43), I² = 0%							

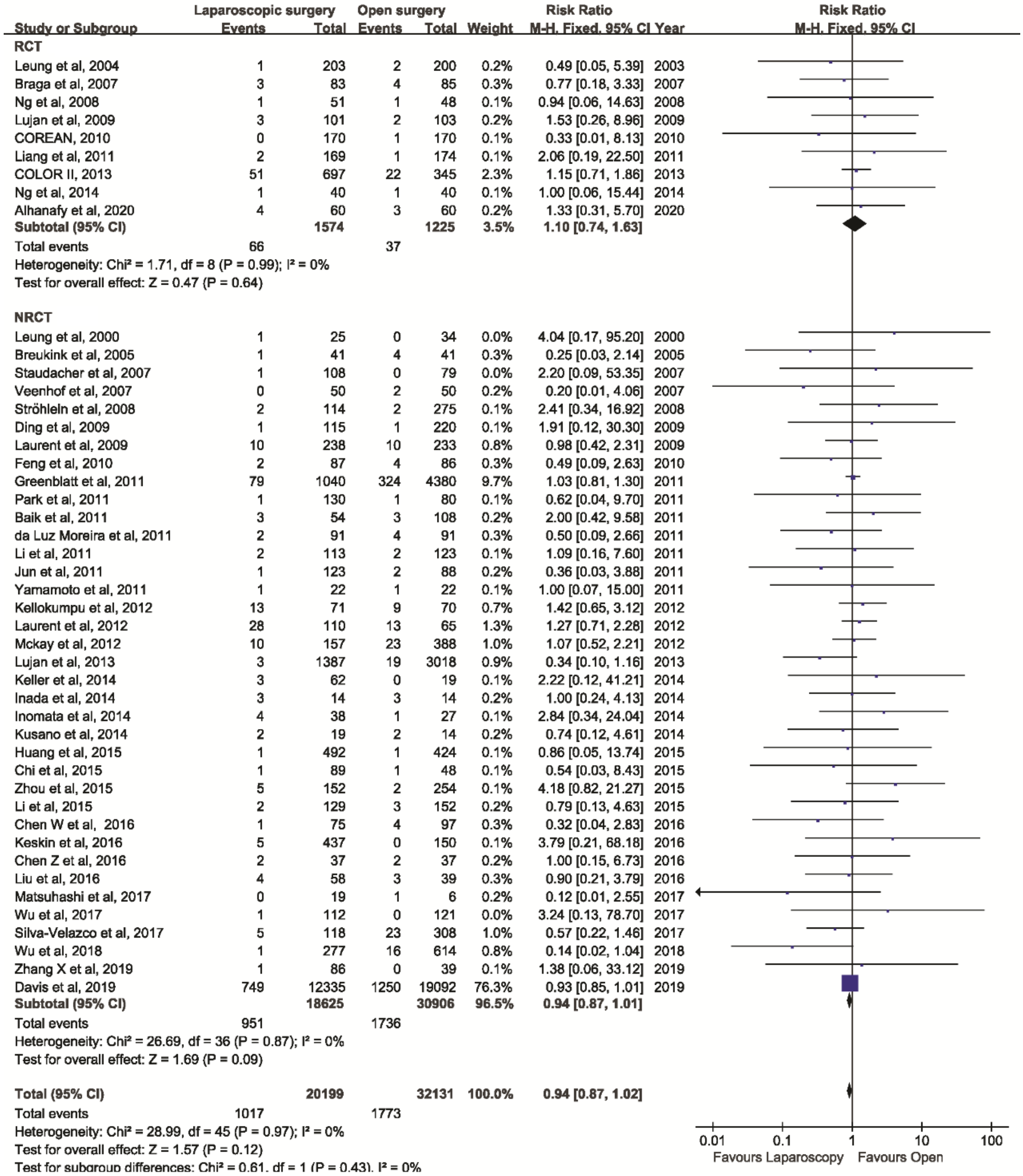

FIGURE S16 Forest plot for anastomotic leak

Study or Subgroup	Laparoscopic surgery Events	Laparoscopic surgery Total	Open surgery Events	Open surgery Total	Weight	Risk Ratio M-H, Fixed, 95% CI	Year
**RCT**							
Leung et al, 2004	9	203	15	200	0.5%	0.59 [0.26, 1.32]	2003
CLASICC, 2005	33	253	15	128	0.7%	1.11 [0.63, 1.97]	2005
Braga et al, 2007	6	83	13	85	0.4%	0.47 [0.19, 1.18]	2007
Ng et al, 2008	0	51	4	48	0.2%	0.10 [0.01, 1.89]	2008
Lujan et al, 2009	0	101	2	103	0.1%	0.20 [0.01, 4.20]	2009
Ng et al, 2009	5	76	9	77	0.3%	0.56 [0.20, 1.60]	2009
Liu et al, 2010	3	98	4	88	0.1%	0.67 [0.16, 2.93]	2010
COREAN, 2010	2	170	11	170	0.4%	0.18 [0.04, 0.81]	2010
Liang et al, 2011	9	169	8	174	0.3%	1.16 [0.46, 2.93]	2011
Gong et al, 2012	1	67	2	71	0.1%	0.53 [0.05, 5.71]	2012
COLOR II, 2013	28	697	17	345	0.8%	0.82 [0.45, 1.47]	2013
Ng et al, 2014	1	40	7	40	0.2%	0.14 [0.02, 1.11]	2014
Yao et al, 2018	1	60	4	60	0.1%	0.25 [0.03, 2.17]	2018
Alhanafy et al, 2020	3	60	15	60	0.5%	0.20 [0.06, 0.66]	2020
**Subtotal (95% CI)**		**2128**		**1649**	**4.6%**	**0.59 [0.46, 0.77]**	
Total events	101		126				
Heterogeneity: Chi² = 18.00, df = 13 (P = 0.16); I² = 28%							
Test for overall effect: Z = 3.88 (P = 0.0001)							
**NRCT**							
Schwandner et al, 1999	3	32	3	32	0.1%	1.00 [0.22, 4.59]	1999
Leung et al, 2000	9	25	16	34	0.5%	0.77 [0.41, 1.44]	2000
Hartley et al, 2001	0	21	1	22	0.0%	0.35 [0.01, 8.11]	2001
Wu et al, 2004	0	18	1	18	0.0%	0.33 [0.01, 7.68]	2004
Sahakitrungruang et al, 2005	0	24	6	25	0.2%	0.08 [0.00, 1.35]	2005
Breukink et al, 2005	2	41	5	41	0.2%	0.40 [0.08, 1.95]	2005
Morino et al, 2005	3	98	6	93	0.2%	0.47 [0.12, 1.84]	2005
Wong et al, 2006	2	71	5	31	0.2%	0.17 [0.04, 0.85]	2006
Law et al, 2006	2	98	4	167	0.1%	0.85 [0.16, 4.57]	2006
Staudacher et al, 2007	5	108	11	79	0.4%	0.33 [0.12, 0.92]	2007
Veenhof et al, 2007	4	50	6	50	0.2%	0.67 [0.20, 2.22]	2007
Lelong et al, 2007	4	104	5	68	0.2%	0.52 [0.15, 1.88]	2007
Ströhlein et al, 2008	4	114	13	275	0.3%	0.74 [0.25, 2.23]	2008
Koulas et al, 2009	0	57	3	60	0.1%	0.15 [0.01, 2.85]	2009
Laurent et al, 2009	4	238	9	233	0.3%	0.44 [0.14, 1.39]	2009
González et al, 2009	3	28	4	28	0.1%	0.75 [0.18, 3.05]	2009
Gouvas et al, 2009	4	45	14	43	0.5%	0.27 [0.10, 0.76]	2009
Ding et al, 2009	0	115	2	220	0.1%	0.38 [0.02, 7.87]	2009
Khaikin et al, 2009	1	32	3	50	0.1%	0.52 [0.06, 4.79]	2009
Feng et al, 2010	3	87	6	86	0.2%	0.49 [0.13, 1.91]	2010
Fujimoto et al, 2010	1	35	2	19	0.1%	0.27 [0.03, 2.80]	2010
Biondo et al, 2010	18	209	34	395	0.8%	1.00 [0.58, 1.73]	2010
da Luz Moreira et al, 2011	1	91	9	91	0.3%	0.11 [0.01, 0.86]	2011
Baik et al, 2011	3	54	6	108	0.1%	1.00 [0.26, 3.85]	2011
Li et al, 2011	2	113	4	123	0.1%	0.54 [0.10, 2.91]	2011
Yamamoto et al, 2011	1	22	4	22	0.1%	0.25 [0.03, 2.06]	2011
Jun et al, 2011	1	123	3	88	0.1%	0.24 [0.03, 2.25]	2011
Greenblatt et al, 2011	62	1040	517	4380	6.6%	0.51 [0.39, 0.65]	2011
Kellokumpu et al, 2012	6	100	17	91	0.6%	0.32 [0.13, 0.78]	2012
Mckay et al, 2012	17	157	60	388	1.2%	0.70 [0.42, 1.16]	2012
Li et al, 2012	12	381	35	276	1.4%	0.25 [0.13, 0.47]	2012
Seshadri et al, 2012	10	72	25	72	0.8%	0.40 [0.21, 0.77]	2012
Lee et al, 2013	0	80	6	80	0.2%	0.08 [0.00, 1.34]	2013
Park et al, 2013	9	406	17	406	0.6%	0.53 [0.24, 1.17]	2013
Lujan et al, 2013	92	1387	311	3018	6.5%	0.64 [0.51, 0.81]	2013
Keller et al, 2014	3	53	1	19	0.0%	1.08 [0.12, 9.72]	2014
Wang et al, 2014	0	100	6	136	0.2%	0.10 [0.01, 1.83]	2014
Zhou et al, 2014	2	57	5	65	0.2%	0.46 [0.09, 2.26]	2014
Inomata et al, 2014	5	38	3	27	0.1%	1.18 [0.31, 4.54]	2014
Kusano et al, 2014	2	19	0	14	0.0%	3.75 [0.19, 72.49]	2014
Inada et al, 2014	1	14	4	14	0.1%	0.25 [0.03, 1.97]	2014
Zeng et al, 2015	8	112	31	182	0.8%	0.42 [0.20, 0.88]	2015
Wang et al, 2015	3	106	5	106	0.2%	0.60 [0.15, 2.45]	2015
Huang et al, 2015	7	492	27	424	1.0%	0.22 [0.10, 0.51]	2015
Chi et al, 2015	1	89	0	48	0.0%	1.63 [0.07, 39.34]	2015
Zhou et al, 2015	6	152	11	254	0.3%	0.91 [0.34, 2.41]	2015
Odermatt et al, 2016	0	48	1	48	0.0%	0.33 [0.01, 7.98]	2016
Chen W et al, 2016	3	75	6	97	0.2%	0.65 [0.17, 2.50]	2016
Nonaka et al, 2016	1	40	3	38	0.1%	0.32 [0.03, 2.91]	2016
Kim et al, 2016	2	486	28	1095	0.6%	0.16 [0.04, 0.67]	2016
Chen Z et al, 2016	0	37	3	37	0.1%	0.14 [0.01, 2.67]	2016
Keskin et al, 2016	11	437	3	150	0.1%	1.26 [0.36, 4.45]	2016
De'Angelis et al, 2017	0	52	1	52	0.0%	0.33 [0.01, 8.00]	2017
Silva-Velazco et al, 2017	2	118	28	304	0.5%	0.18 [0.04, 0.76]	2017
Wu et al, 2017	23	112	30	121	1.0%	0.83 [0.51, 1.34]	2017
Wu et al, 2018	16	277	63	614	1.3%	0.56 [0.33, 0.96]	2018
Hida et al, 2018	28	482	38	482	1.3%	0.74 [0.46, 1.18]	2018
Andersen et al, 2018	119	3099	208	3090	6.9%	0.57 [0.46, 0.71]	2018
Garfinkle et al, 2018	1	34	14	201	0.1%	0.42 [0.06, 3.11]	2018
Zhang Z et al, 2019	2	112	10	116	0.3%	0.21 [0.05, 0.92]	2019
Zhang X et al, 2019	4	86	2	39	0.1%	0.91 [0.17, 4.74]	2019
Davis et al, 2019	660	12335	2150	19092	56.2%	0.48 [0.44, 0.52]	2019
**Subtotal (95% CI)**		**24738**		**38077**	**95.4%**	**0.50 [0.47, 0.53]**	
Total events	1198		3854				
Heterogeneity: Chi² = 65.41, df = 61 (P = 0.33); I² = 7%							
Test for overall effect: Z = 21.45 (P < 0.00001)							
**Total (95% CI)**		**26866**		**39726**	**100.0%**	**0.50 [0.47, 0.54]**	
Total events	1299		3980				
Heterogeneity: Chi² = 85.99, df = 75 (P = 0.18); I² = 13%							
Test for overall effect: Z = 21.79 (P < 0.00001)							
Test for subgroup differences: Chi² = 1.52, df = 1 (P = 0.22), I² = 34.1%							

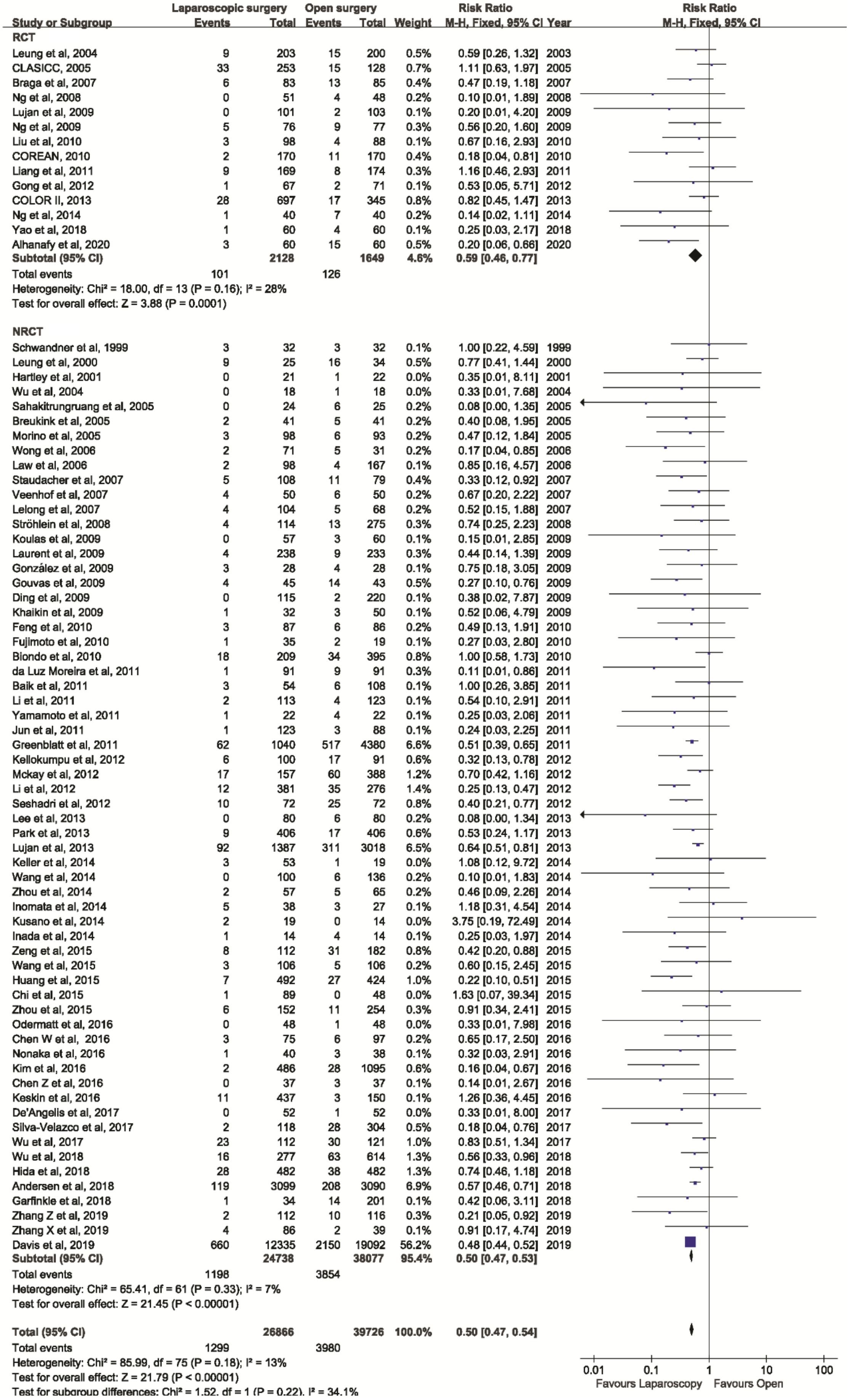

FIGURE S17 Forest plot for intra-abdominal, pelvic or retroperitoneal abscess

Study or Subgroup	Laparoscopic surgery Events	Laparoscopic surgery Total	Open surgery Events	Open surgery Total	Weight	Risk Ratio M-H, Fixed, 95% CI	Year
**RCT**							
Leung et al, 2004	9	203	15	200	0.5%	0.59 [0.26, 1.32]	2003
CLASICC, 2005	33	253	15	128	0.7%	1.11 [0.63, 1.97]	2005
Braga et al, 2007	6	83	13	85	0.4%	0.47 [0.19, 1.18]	2007
Ng et al, 2008	0	51	4	48	0.2%	0.10 [0.01, 1.89]	2008
Lujan et al, 2009	0	101	2	103	0.1%	0.20 [0.01, 4.20]	2009
Ng et al, 2009	5	76	9	77	0.3%	0.56 [0.20, 1.60]	2009
Liu et al, 2010	3	98	4	88	0.1%	0.67 [0.16, 2.93]	2010
COREAN, 2010	2	170	11	170	0.4%	0.18 [0.04, 0.81]	2010
Liang et al, 2011	9	169	8	174	0.3%	1.16 [0.46, 2.93]	2011
Gong et al, 2012	1	67	2	71	0.1%	0.53 [0.05, 5.71]	2012
COLOR II, 2013	28	697	17	345	0.8%	0.82 [0.45, 1.47]	2013
Ng et al, 2014	1	40	7	40	0.2%	0.14 [0.02, 1.11]	2014
Yao et al, 2018	1	60	4	60	0.1%	0.25 [0.03, 2.17]	2018
Alhanafy et al, 2020	3	60	15	60	0.5%	0.20 [0.06, 0.66]	2020
**Subtotal (95% CI)**		**2128**		**1649**	**4.6%**	**0.59 [0.46, 0.77]**	
Total events	101		126				
Heterogeneity: Chi² = 18.00, df = 13 (P = 0.16); I² = 28%							
Test for overall effect: Z = 3.88 (P = 0.0001)							
**NRCT**							
Schwandner et al, 1999	3	32	3	32	0.1%	1.00 [0.22, 4.59]	1999
Leung et al, 2000	9	25	16	34	0.5%	0.77 [0.41, 1.44]	2000
Hartley et al, 2001	0	21	1	22	0.0%	0.35 [0.01, 8.11]	2001
Wu et al, 2004	0	18	1	18	0.0%	0.33 [0.01, 7.68]	2004
Sahakitrungruang et al, 2005	0	24	6	25	0.2%	0.08 [0.00, 1.35]	2005
Breukink et al, 2005	2	41	5	41	0.2%	0.40 [0.08, 1.95]	2005
Morino et al, 2005	3	98	6	93	0.2%	0.47 [0.12, 1.84]	2005
Wong et al, 2006	2	71	5	31	0.2%	0.17 [0.04, 0.85]	2006
Law et al, 2006	2	98	4	167	0.1%	0.85 [0.16, 4.57]	2006
Staudacher et al, 2007	5	108	11	79	0.4%	0.33 [0.12, 0.92]	2007
Veenhof et al, 2007	4	50	6	50	0.2%	0.67 [0.20, 2.22]	2007
Lelong et al, 2007	4	104	5	68	0.2%	0.52 [0.15, 1.88]	2007
Ströhlein et al, 2008	4	114	13	275	0.3%	0.74 [0.25, 2.23]	2008
Koulas et al, 2009	0	57	3	60	0.1%	0.15 [0.01, 2.85]	2009
Laurent et al, 2009	4	238	9	233	0.3%	0.44 [0.14, 1.39]	2009
González et al, 2009	3	28	4	28	0.1%	0.75 [0.18, 3.05]	2009
Gouvas et al, 2009	4	45	14	43	0.5%	0.27 [0.10, 0.76]	2009
Ding et al, 2009	0	115	2	220	0.1%	0.38 [0.02, 7.87]	2009
Khaikin et al, 2009	1	32	3	50	0.1%	0.52 [0.06, 4.79]	2009
Feng et al, 2010	3	87	6	86	0.2%	0.49 [0.13, 1.91]	2010
Fujimoto et al, 2010	1	35	2	19	0.1%	0.27 [0.03, 2.80]	2010
Biondo et al, 2010	18	209	34	395	0.8%	1.00 [0.58, 1.73]	2010
da Luz Moreira et al, 2011	1	91	9	91	0.3%	0.11 [0.01, 0.86]	2011
Baik et al, 2011	3	54	6	108	0.1%	1.00 [0.26, 3.85]	2011
Li et al, 2011	2	113	4	123	0.1%	0.54 [0.10, 2.91]	2011
Yamamoto et al, 2011	1	22	4	22	0.1%	0.25 [0.03, 2.06]	2011
Jun et al, 2011	1	123	3	88	0.1%	0.24 [0.03, 2.25]	2011
Greenblatt et al, 2011	62	1040	517	4380	6.6%	0.51 [0.39, 0.65]	2011
Kellokumpu et al, 2012	6	100	17	91	0.6%	0.32 [0.13, 0.78]	2012
Mckay et al, 2012	17	157	60	388	1.2%	0.70 [0.42, 1.16]	2012
Li et al, 2012	12	381	35	276	1.4%	0.25 [0.13, 0.47]	2012
Seshadri et al, 2012	10	72	25	72	0.8%	0.40 [0.21, 0.77]	2012
Lee et al, 2013	0	80	6	80	0.2%	0.08 [0.00, 1.34]	2013
Park et al, 2013	9	406	17	406	0.6%	0.53 [0.24, 1.17]	2013
Lujan et al, 2013	92	1387	311	3018	6.5%	0.64 [0.51, 0.81]	2013
Keller et al, 2014	3	53	1	19	0.0%	1.08 [0.12, 9.72]	2014
Wang et al, 2014	0	100	6	136	0.2%	0.10 [0.01, 1.83]	2014
Zhou et al, 2014	2	57	5	65	0.2%	0.46 [0.09, 2.26]	2014
Inomata et al, 2014	5	38	3	27	0.1%	1.18 [0.31, 4.54]	2014
Kusano et al, 2014	2	19	0	14	0.0%	3.75 [0.19, 72.49]	2014
Inada et al, 2014	1	14	4	14	0.1%	0.25 [0.03, 1.97]	2014
Zeng et al, 2015	8	112	31	182	0.8%	0.42 [0.20, 0.88]	2015
Wang et al, 2015	3	106	5	106	0.2%	0.60 [0.15, 2.45]	2015
Huang et al, 2015	7	492	27	424	1.0%	0.22 [0.10, 0.51]	2015
Chi et al, 2015	1	89	0	48	0.0%	1.63 [0.07, 39.34]	2015
Zhou et al, 2015	6	152	11	254	0.3%	0.91 [0.34, 2.41]	2015
Odermatt et al, 2016	0	48	1	48	0.0%	0.33 [0.01, 7.98]	2016
Chen W et al, 2016	3	75	6	97	0.2%	0.65 [0.17, 2.50]	2016
Nonaka et al, 2016	1	40	3	38	0.1%	0.32 [0.03, 2.91]	2016
Kim et al, 2016	2	486	28	1095	0.6%	0.16 [0.04, 0.67]	2016
Chen Z et al, 2016	0	37	3	37	0.1%	0.14 [0.01, 2.67]	2016
Keskin et al, 2016	11	437	3	150	0.1%	1.26 [0.36, 4.45]	2016
De'Angelis et al, 2017	0	52	1	52	0.0%	0.33 [0.01, 8.00]	2017
Silva-Velazco et al, 2017	2	118	28	304	0.5%	0.18 [0.04, 0.76]	2017
Wu et al, 2017	23	112	30	121	1.0%	0.83 [0.51, 1.34]	2017
Wu et al, 2018	16	277	63	614	1.3%	0.56 [0.33, 0.96]	2018
Hida et al, 2018	28	482	38	482	1.3%	0.74 [0.46, 1.18]	2018
Andersen et al, 2018	119	3099	208	3090	6.9%	0.57 [0.46, 0.71]	2018
Garfinkle et al, 2018	1	34	14	201	0.1%	0.42 [0.06, 3.11]	2018
Zhang Z et al, 2019	2	112	10	116	0.3%	0.21 [0.05, 0.92]	2019
Zhang X et al, 2019	4	86	2	39	0.1%	0.91 [0.17, 4.74]	2019
Davis et al, 2019	660	12335	2150	19092	56.2%	0.48 [0.44, 0.52]	2019
**Subtotal (95% CI)**		**24738**		**38077**	**95.4%**	**0.50 [0.47, 0.53]**	
Total events	1198		3854				
Heterogeneity: Chi² = 65.41, df = 61 (P = 0.33); I² = 7%							
Test for overall effect: Z = 21.45 (P < 0.00001)							
**Total (95% CI)**		**26866**		**39726**	**100.0%**	**0.50 [0.47, 0.54]**	
Total events	1299		3980				
Heterogeneity: Chi² = 85.99, df = 75 (P = 0.18); I² = 13%							
Test for overall effect: Z = 21.79 (P < 0.00001)							
Test for subgroup differences: Chi² = 1.52, df = 1 (P = 0.22), I² = 34.1%							

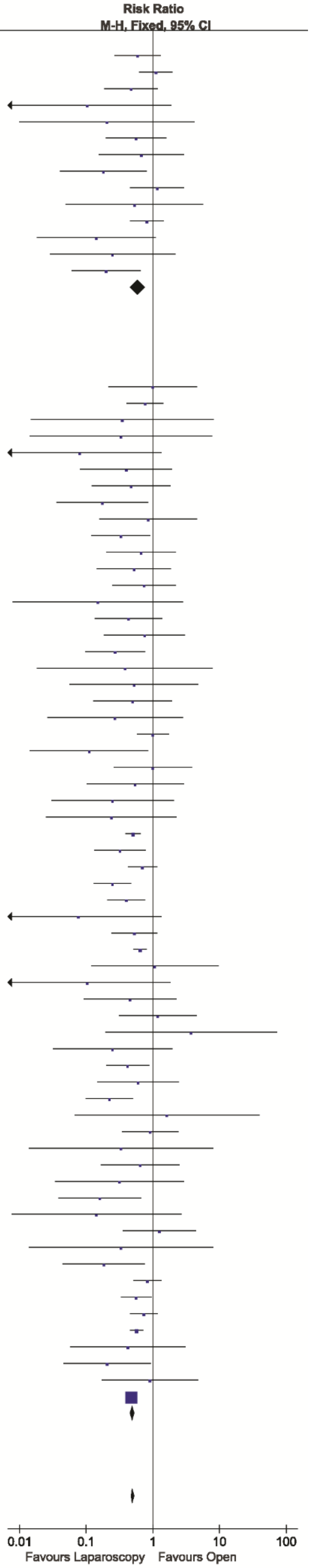

FIGURE S18 Forest plot for reintervention within 30 days

Study or Subgroup	Laparoscopic surgery Events	Laparoscopic surgery Total	Open surgery Events	Open surgery Total	Weight	Risk Ratio M-H, Fixed, 95% CI	Year
**RCT**							
Leung et al, 2004	6	203	5	200	0.9%	1.18 [0.37, 3.81]	2003
Braga et al, 2007	6	83	11	85	1.9%	0.56 [0.22, 1.44]	2007
Ng et al, 2008	1	51	4	48	0.7%	0.24 [0.03, 2.03]	2008
Ng et al, 2009	2	76	4	77	0.7%	0.51 [0.10, 2.68]	2009
Lujan et al, 2009	8	101	10	103	1.7%	0.82 [0.34, 1.98]	2009
COREAN, 2010	3	170	3	170	0.5%	1.00 [0.20, 4.88]	2010
COLOR II, 2013	113	697	52	345	12.1%	1.08 [0.80, 1.45]	2013
Z6051, 2015	12	240	5	222	0.9%	2.22 [0.79, 6.20]	2015
Alhanafy et al, 2020	15	60	15	60	2.6%	1.00 [0.54, 1.86]	2020
**Subtotal (95% CI)**		**1681**		**1310**	**22.1%**	**1.01 [0.80, 1.27]**	
Total events	166		109				
Heterogeneity: Chi² = 6.63, df = 8 (P = 0.58); I² = 0%							
Test for overall effect: Z = 0.05 (P = 0.96)							
**NRCT**							
Morino et al, 2005	6	98	3	93	0.5%	1.90 [0.49, 7.37]	2005
Breukink et al, 2005	5	41	8	41	1.4%	0.63 [0.22, 1.75]	2005
Lelong et al, 2007	9	104	5	68	1.1%	1.18 [0.41, 3.36]	2007
Veenhof et al, 2007	7	50	7	50	1.2%	1.00 [0.38, 2.64]	2007
Law et al, 2009	6	111	13	310	1.2%	1.29 [0.50, 3.31]	2009
Ding et al, 2009	6	115	12	220	1.4%	0.96 [0.37, 2.48]	2009
Feng et al, 2010	2	87	5	86	0.9%	0.40 [0.08, 1.98]	2010
Biondo et al, 2010	16	209	40	395	4.8%	0.76 [0.43, 1.32]	2010
da Luz Moreira et al, 2011	3	91	5	91	0.9%	0.60 [0.15, 2.44]	2011
Greenblatt et al, 2011	6	1040	48	4380	3.2%	0.53 [0.23, 1.23]	2011
Laurent et al, 2012	20	110	14	65	3.1%	0.84 [0.46, 1.55]	2012
Kellokumpu et al, 2012	6	100	8	91	1.5%	0.68 [0.25, 1.89]	2012
Mckay et al, 2012	17	157	25	388	2.5%	1.68 [0.93, 3.02]	2012
Lujan et al, 2013	117	1387	290	3018	31.9%	0.88 [0.72, 1.08]	2013
Zhou et al, 2014	0	57	2	65	0.4%	0.23 [0.01, 4.64]	2014
Keller et al, 2014	4	62	2	19	0.5%	0.61 [0.12, 3.09]	2014
Li et al, 2015	10	129	12	152	1.9%	0.98 [0.44, 2.20]	2015
Zeng et al, 2015	1	112	2	182	0.3%	0.81 [0.07, 8.86]	2015
Chen Z et al, 2016	2	37	5	37	0.9%	0.40 [0.08, 1.93]	2016
Odermatt et al, 2016	2	48	3	48	0.5%	0.67 [0.12, 3.81]	2016
Silva-Velazco et al, 2017	11	118	19	304	1.9%	1.49 [0.73, 3.04]	2017
De'Angelis et al, 2017	6	52	4	52	0.7%	1.50 [0.45, 5.01]	2017
Wu et al, 2018	10	277	29	614	3.1%	0.76 [0.38, 1.55]	2018
Hida et al, 2018	17	482	22	482	3.8%	0.77 [0.42, 1.44]	2018
Tayar et al, 2018	0	50	3	50	0.6%	0.14 [0.01, 2.70]	2018
Manchon-Walsh et al, 2019	87	842	35	517	7.6%	1.53 [1.05, 2.22]	2019
Zhang X et al, 2019	1	86	0	39	0.1%	1.38 [0.06, 33.12]	2019
**Subtotal (95% CI)**		**6052**		**11857**	**77.9%**	**0.94 [0.83, 1.07]**	
Total events	377		621				
Heterogeneity: Chi² = 24.15, df = 26 (P = 0.57); I² = 0%							
Test for overall effect: Z = 0.89 (P = 0.37)							
**Total (95% CI)**		**7733**		**13167**	**100.0%**	**0.96 [0.86, 1.07]**	
Total events	543		730				
Heterogeneity: Chi² = 30.97, df = 35 (P = 0.66); I² = 0%							
Test for overall effect: Z = 0.77 (P = 0.44)							
Test for subgroup differences: Chi² = 0.23, df = 1 (P = 0.63), I² = 0%							

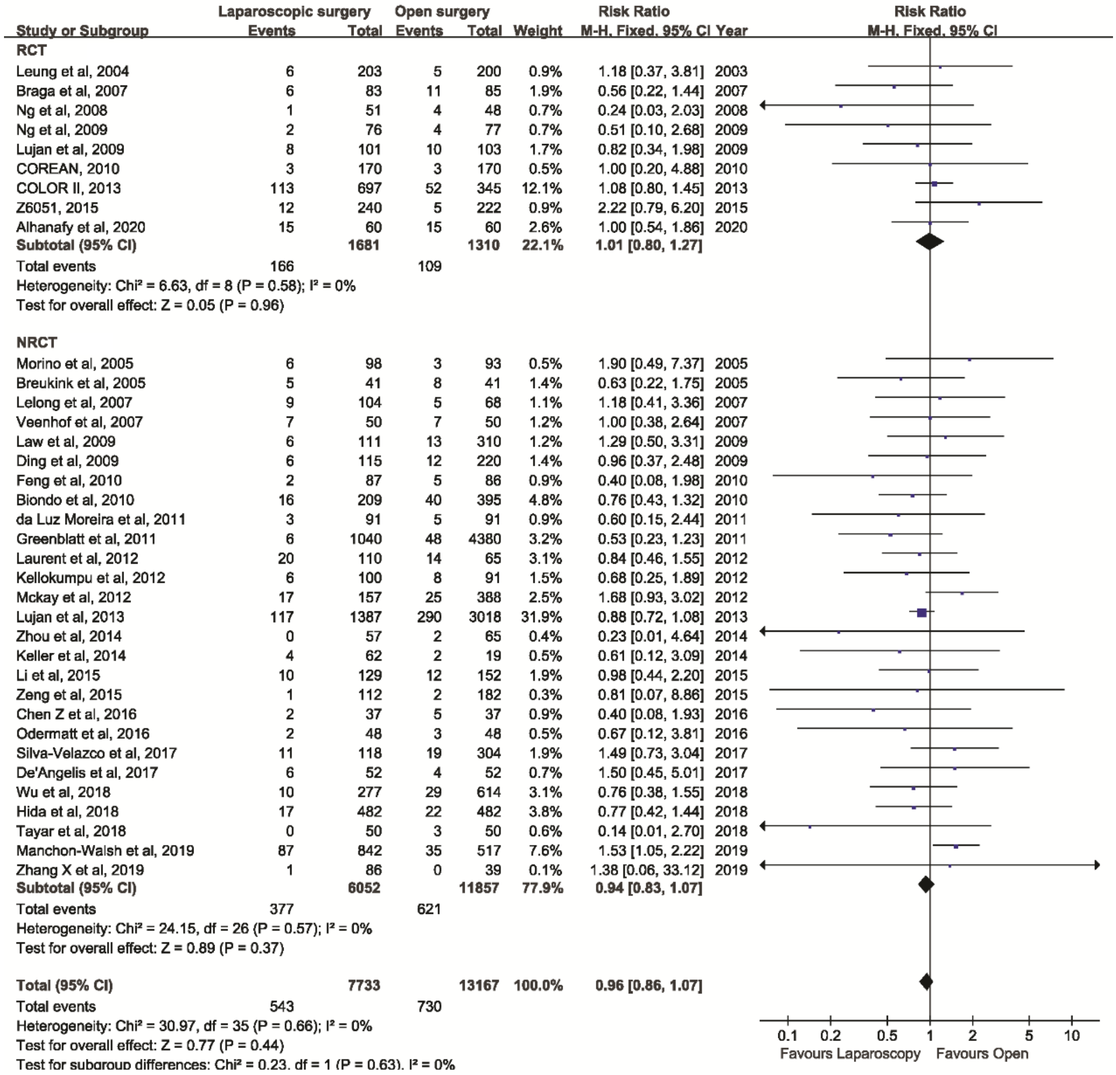

FIGURE S19 Forest plot for mortality within 30 days

	Laparoscopic surgery		Open surgery			Risk Ratio	
Study or Subgroup	Events	Total	Events	Total	Weight	M-H, Fixed, 95% CI	Year
**RCT**							
Leung et al, 2004	5	203	4	200	0.6%	1.23 [0.34, 4.52]	2003
Braga et al, 2007	1	83	1	85	0.2%	1.02 [0.07, 16.10]	2007
Ng et al, 2008	1	51	1	48	0.2%	0.94 [0.06, 14.63]	2008
Lujan et al, 2009	2	101	3	103	0.5%	0.68 [0.12, 3.98]	2009
Ng et al, 2009	2	76	3	77	0.5%	0.68 [0.12, 3.93]	2009
COLOR II, 2013	8	699	6	345	1.2%	0.66 [0.23, 1.88]	2013
Z6051, 2015	2	240	2	222	0.3%	0.93 [0.13, 6.51]	2015
Alhanafy et al, 2020	0	60	1	60	0.2%	0.33 [0.01, 8.02]	2020
**Subtotal (95% CI)**		**1513**		**1140**	**3.6%**	**0.79 [0.44, 1.43]**	
Total events	21		21				
Heterogeneity: Chi² = 0.98, df = 7 (P = 1.00); I² = 0%							
Test for overall effect: Z = 0.77 (P = 0.44)							
**NRCT**							
Anthuber et al, 2003	0	101	5	334	0.4%	0.30 [0.02, 5.35]	2003
Morino et al, 2005	1	98	2	93	0.3%	0.47 [0.04, 5.15]	2005
Breukink et al, 2005	0	41	1	41	0.2%	0.33 [0.01, 7.95]	2005
Law et al, 2006	1	98	4	167	0.5%	0.43 [0.05, 3.76]	2006
Veenhof et al, 2007	1	50	1	50	0.2%	1.00 [0.06, 15.55]	2007
Lelong et al, 2007	1	104	2	68	0.4%	0.33 [0.03, 3.54]	2007
Ströhlein et al, 2008	0	114	8	275	0.8%	0.14 [0.01, 2.43]	2008
Law et al, 2009	0	111	3	310	0.3%	0.40 [0.02, 7.62]	2009
Ding et al, 2009	0	115	2	220	0.3%	0.38 [0.02, 7.87]	2009
Laurent et al, 2009	2	238	6	233	0.9%	0.33 [0.07, 1.60]	2009
Biondo et al, 2010	1	209	14	395	1.5%	0.13 [0.02, 1.02]	2010
da Luz Moreira et al, 2011	0	91	1	91	0.2%	0.33 [0.01, 8.08]	2011
Li et al, 2011	1	113	1	123	0.1%	1.09 [0.07, 17.20]	2011
Baik et al, 2011	0	54	2	108	0.3%	0.40 [0.02, 8.11]	2011
Park et al, 2011	0	130	1	80	0.3%	0.21 [0.01, 5.00]	2011
Jun et al, 2011	0	123	1	88	0.3%	0.24 [0.01, 5.81]	2011
Greenblatt et al, 2011	8	1040	44	4380	2.6%	0.77 [0.36, 1.62]	2011
Mckay et al, 2012	3	157	3	388	0.3%	2.47 [0.50, 12.11]	2012
Kellokumpu et al, 2012	1	100	3	91	0.5%	0.30 [0.03, 2.86]	2012
Jefferies et al, 2012	4	16	9	25	1.1%	0.69 [0.26, 1.88]	2012
Penninckx et al, 2013	11	764	28	1896	2.5%	0.97 [0.49, 1.95]	2013
Park et al, 2013	1	406	2	406	0.3%	0.50 [0.05, 5.49]	2013
Lujan et al, 2013	16	1387	108	3018	10.4%	0.32 [0.19, 0.54]	2013
Lee et al, 2013	1	80	0	80	0.1%	3.00 [0.12, 72.56]	2013
Zhou et al, 2014	0	57	2	65	0.4%	0.23 [0.01, 4.64]	2014
Keller et al, 2014	1	62	0	19	0.1%	0.95 [0.04, 22.47]	2014
Zeng et al, 2015	5	112	9	182	1.0%	0.90 [0.31, 2.63]	2015
Huang et al, 2015	2	492	2	424	0.3%	0.86 [0.12, 6.09]	2015
Li et al, 2015	2	129	2	152	0.3%	1.18 [0.17, 8.25]	2015
Nussbaum et al, 2015	84	6430	108	6430	16.5%	0.78 [0.59, 1.03]	2015
Zhou et al, 2015	4	152	5	254	0.6%	1.34 [0.36, 4.90]	2015
Keskin et al, 2016	1	437	0	150	0.1%	1.03 [0.04, 25.25]	2016
Odermatt et al, 2016	1	48	2	48	0.3%	0.50 [0.05, 5.33]	2016
De'Angelis et al, 2017	3	52	6	52	0.9%	0.50 [0.13, 1.89]	2017
Garfinkle et al, 2018	1	34	3	201	0.1%	1.97 [0.21, 18.40]	2018
Chen et al, 2018	62	5578	1205	102858	18.9%	0.95 [0.74, 1.22]	2018
Wu et al, 2018	1	277	4	614	0.4%	0.55 [0.06, 4.94]	2018
Manchon-Walsh et al, 2019	11	842	17	517	3.2%	0.40 [0.19, 0.84]	2019
Davis et al, 2019	83	12335	233	19092	27.9%	0.55 [0.43, 0.71]	2019
Mar et al, 2020	6	400	5	201	1.0%	0.60 [0.19, 1.95]	2020
**Subtotal (95% CI)**		**33177**		**144219**	**96.4%**	**0.66 [0.58, 0.75]**	
Total events	320		1854				
Heterogeneity: Chi² = 36.22, df = 39 (P = 0.60); I² = 0%							
Test for overall effect: Z = 6.55 (P < 0.00001)							
**Total (95% CI)**		**34690**		**145359**	**100.0%**	**0.66 [0.59, 0.75]**	
Total events	341		1875				
Heterogeneity: Chi² = 37.43, df = 47 (P = 0.84); I² = 0%							
Test for overall effect: Z = 6.58 (P < 0.00001)							
Test for subgroup differences: Chi² = 0.36, df = 1 (P = 0.55), I² = 0%							

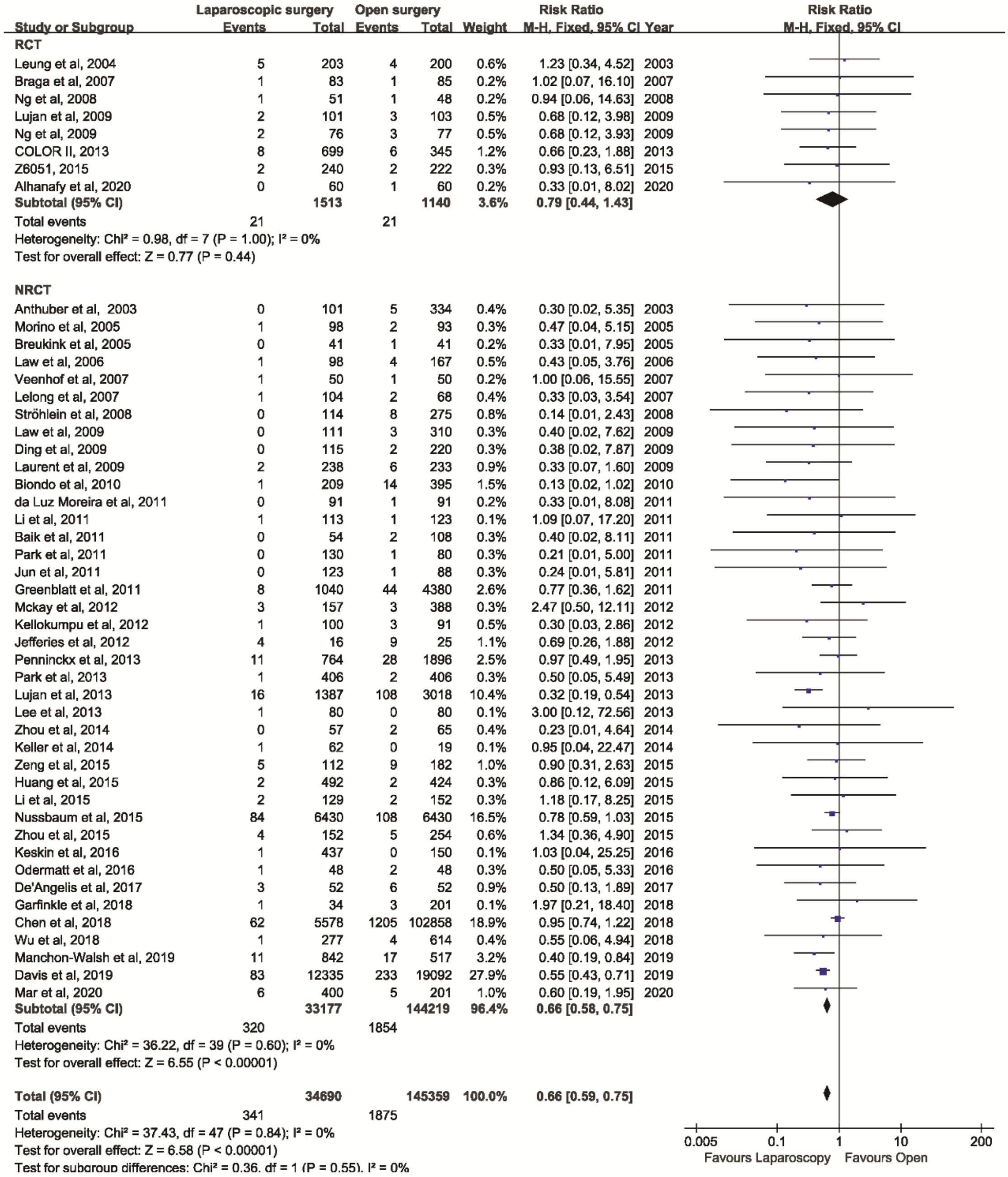

FIGURE S20 Forest plot for 3-year local recurrence

Study or Subgroup	Laparoscopic surgery Events	Laparoscopic surgery Total	Open surgery Events	Open surgery Total	Weight	Risk Ratio M-H, Fixed, 95% CI	Year
**RCT**							
Araujo et al, 2003	0	13	2	13	0.8%	0.20 [0.01, 3.80]	2003
CLASICC, 2005	25	253	13	128	5.2%	0.97 [0.52, 1.84]	2005
Braga et al, 2007	3	83	4	85	1.2%	0.77 [0.18, 3.33]	2007
COREAN, 2010	2	170	4	170	1.2%	0.50 [0.09, 2.69]	2010
COLOR II, 2013	31	588	15	300	6.0%	1.05 [0.58, 1.92]	2013
Z6051, 2015	5	240	4	222	1.3%	1.16 [0.31, 4.25]	2015
ALaCaRT, 2015	15	225	9	225	2.7%	1.67 [0.74, 3.73]	2015
**Subtotal (95% CI)**		**1572**		**1143**	**18.4%**	**1.04 [0.74, 1.46]**	
Total events	81		51				
Heterogeneity: Chi² = 3.48, df = 6 (P = 0.75); I² = 0%							
Test for overall effect: Z = 0.22 (P = 0.83)							
**NRCT**							
Schwandner et al, 1999	1	32	0	32	0.2%	3.00 [0.13, 71.00]	1999
Leung et al, 2000	1	25	2	34	0.5%	0.68 [0.07, 7.09]	2000
Hartley et al, 2001	1	21	1	22	0.3%	1.05 [0.07, 15.69]	2001
Feliciotti et al, 2003	10	48	6	33	2.2%	1.15 [0.46, 2.85]	2003
Law et al, 2006	3	97	8	163	1.8%	0.63 [0.17, 2.32]	2006
Wong et al, 2006	3	71	4	31	1.7%	0.33 [0.08, 1.38]	2006
Staudacher et al, 2007	7	108	4	79	1.4%	1.28 [0.39, 4.22]	2007
Veenhof et al, 2007	2	49	6	49	1.8%	0.33 [0.07, 1.57]	2007
Ströhlein et al, 2008	6	114	26	267	4.7%	0.54 [0.23, 1.28]	2008
Ding et al, 2009	4	109	10	206	2.1%	0.76 [0.24, 2.35]	2009
Park et al, 2011	3	110	6	79	2.1%	0.36 [0.09, 1.39]	2011
Seshadri et al, 2012	4	72	7	72	2.1%	0.57 [0.17, 1.87]	2012
Park et al, 2013	15	405	24	404	7.3%	0.62 [0.33, 1.17]	2013
Kusano et al, 2014	1	19	3	14	1.0%	0.25 [0.03, 2.12]	2014
Keller et al, 2014	1	61	1	19	0.5%	0.31 [0.02, 4.75]	2014
Chi et al, 2015	3	89	3	48	1.2%	0.54 [0.11, 2.57]	2015
Zeng et al, 2015	10	107	15	173	3.5%	1.08 [0.50, 2.31]	2015
Wang et al, 2015	3	106	1	106	0.3%	3.00 [0.32, 28.38]	2015
Liu et al, 2016	6	58	5	39	1.8%	0.81 [0.26, 2.46]	2016
Kim et al, 2016	17	486	30	1095	5.6%	1.28 [0.71, 2.29]	2016
Odermatt et al, 2016	4	47	4	46	1.2%	0.98 [0.26, 3.68]	2016
Nonaka et al, 2016	3	40	6	38	1.9%	0.47 [0.13, 1.77]	2016
Hida et al, 2018	49	482	41	482	12.5%	1.20 [0.81, 1.77]	2018
Wu et al, 2018	12	277	46	614	8.7%	0.58 [0.31, 1.07]	2018
Chen et al, 2019	3	64	2	23	0.9%	0.54 [0.10, 3.02]	2019
Manchon-Walsh et al, 2019	29	842	38	517	14.3%	0.47 [0.29, 0.75]	2019
**Subtotal (95% CI)**		**3939**		**4685**	**81.6%**	**0.76 [0.64, 0.90]**	
Total events	201		299				
Heterogeneity: Chi² = 24.72, df = 25 (P = 0.48); I² = 0%							
Test for overall effect: Z = 3.09 (P = 0.002)							
**Total (95% CI)**		**5511**		**5828**	**100.0%**	**0.81 [0.69, 0.95]**	
Total events	282		350				
Heterogeneity: Chi² = 30.68, df = 32 (P = 0.53); I² = 0%							
Test for overall effect: Z = 2.65 (P = 0.008)							
Test for subgroup differences: Chi² = 2.57, df = 1 (P = 0.11), I² = 61.1%							

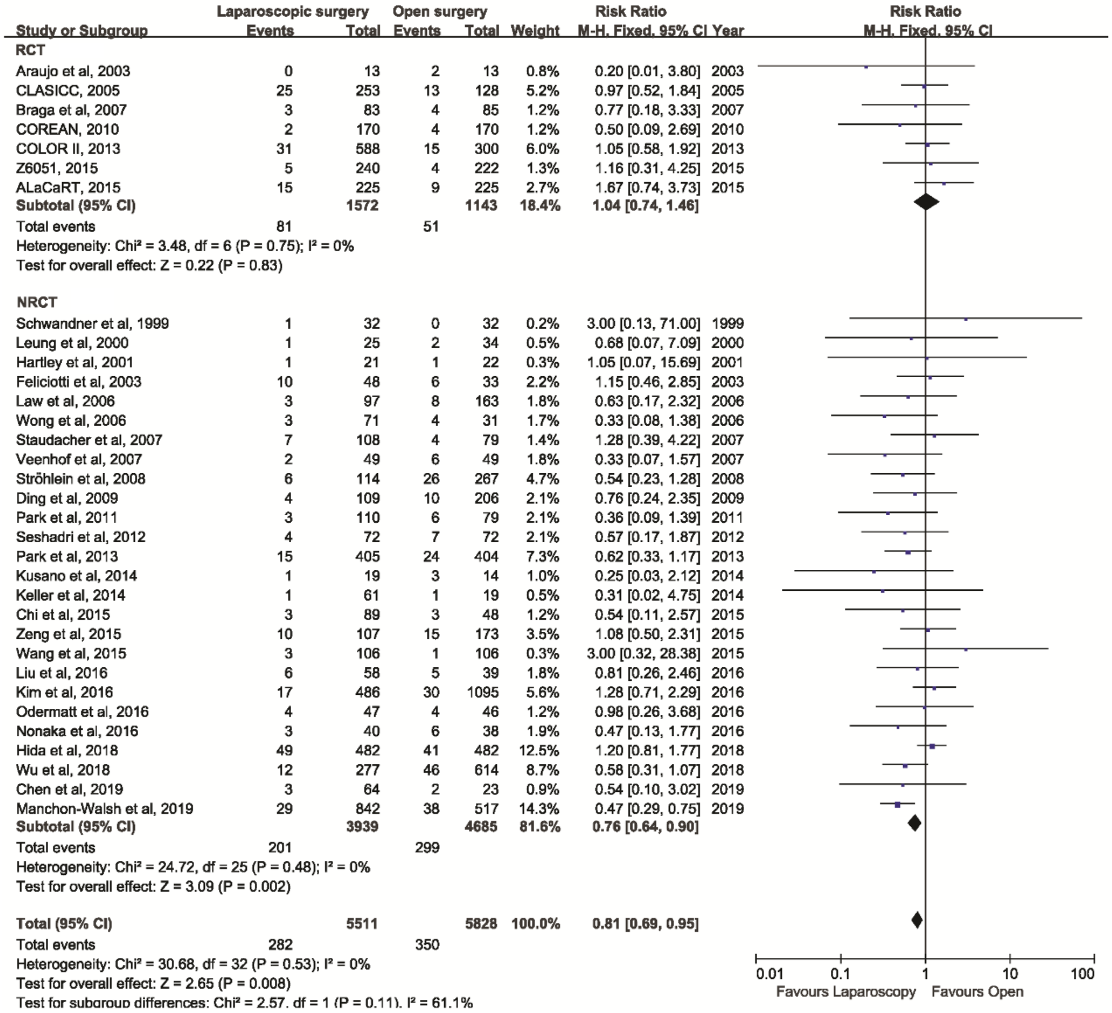

FIGURE S21 Forest plot for 3-year distant recurrence

Study or Subgroup	Laparoscopic surgery Events	Laparoscopic surgery Total	Open surgery Events	Open surgery Total	Weight	Risk Ratio M-H, Fixed, 95% CI	Year
**RCT**							
Leung et al, 2004	11	167	7	170	1.6%	1.60 [0.64, 4.03]	2003
Ng et al, 2008	2	40	4	36	1.0%	0.45 [0.09, 2.31]	2008
Lujan et al, 2009	5	97	5	96	1.2%	0.99 [0.30, 3.31]	2009
Ng et al, 2014	1	36	3	36	0.7%	0.33 [0.04, 3.06]	2014
**Subtotal (95% CI)**		**340**		**338**	**4.4%**	**0.99 [0.53, 1.83]**	
Total events	19		19				
Heterogeneity: Chi² = 2.86, df = 3 (P = 0.41); I² = 0%							
Test for overall effect: Z = 0.03 (P = 0.97)							
**NRCT**							
Morino et al, 2005	3	94	11	89	2.6%	0.26 [0.07, 0.90]	2005
Law et al, 2009	9	111	27	307	3.3%	0.92 [0.45, 1.90]	2009
Kim et al, 2009	21	272	41	135	12.6%	0.25 [0.16, 0.41]	2009
Laurent et al, 2009	9	236	12	227	2.8%	0.72 [0.31, 1.68]	2009
Feng et al, 2010	4	87	4	86	0.9%	0.99 [0.26, 3.83]	2010
Li et al, 2011	10	112	8	122	1.8%	1.36 [0.56, 3.33]	2011
Baik et al, 2011	1	54	4	106	0.6%	0.49 [0.06, 4.28]	2011
Kellokumpu et al, 2012	6	99	7	88	1.7%	0.76 [0.27, 2.18]	2012
Laurent et al, 2012	5	110	1	65	0.3%	2.95 [0.35, 24.74]	2012
Gunka et al, 2012	8	71	8	68	1.9%	0.96 [0.38, 2.41]	2012
Park et al, 2013	23	405	35	404	8.1%	0.66 [0.39, 1.09]	2013
Zhou et al, 2014	3	53	1	59	0.2%	3.34 [0.36, 31.14]	2014
Zhou et al, 2015	24	148	41	249	7.0%	0.98 [0.62, 1.56]	2015
Li et al, 2015	10	127	11	150	2.3%	1.07 [0.47, 2.45]	2015
Kim et al, 2015	3	131	10	176	2.0%	0.40 [0.11, 1.44]	2015
Guo et al, 2015	6	191	6	191	1.4%	1.00 [0.33, 3.05]	2015
Keskin et al, 2016	27	413	17	142	5.8%	0.55 [0.31, 0.97]	2016
Aydın et al, 2018	4	71	3	50	0.8%	0.94 [0.22, 4.01]	2018
Draeger et al, 2018	70	428	301	1079	39.4%	0.59 [0.46, 0.74]	2018
**Subtotal (95% CI)**		**3213**		**3793**	**95.6%**	**0.64 [0.55, 0.74]**	
Total events	246		548				
Heterogeneity: Chi² = 32.34, df = 18 (P = 0.02); I² = 44%							
Test for overall effect: Z = 6.04 (P < 0.00001)							
**Total (95% CI)**		**3553**		**4131**	**100.0%**	**0.66 [0.57, 0.75]**	
Total events	265		567				
Heterogeneity: Chi² = 37.06, df = 22 (P = 0.02); I² = 41%							
Test for overall effect: Z = 5.88 (P < 0.00001)							
Test for subgroup differences: Chi² = 1.81, df = 1 (P = 0.18), I² = 44.8%							

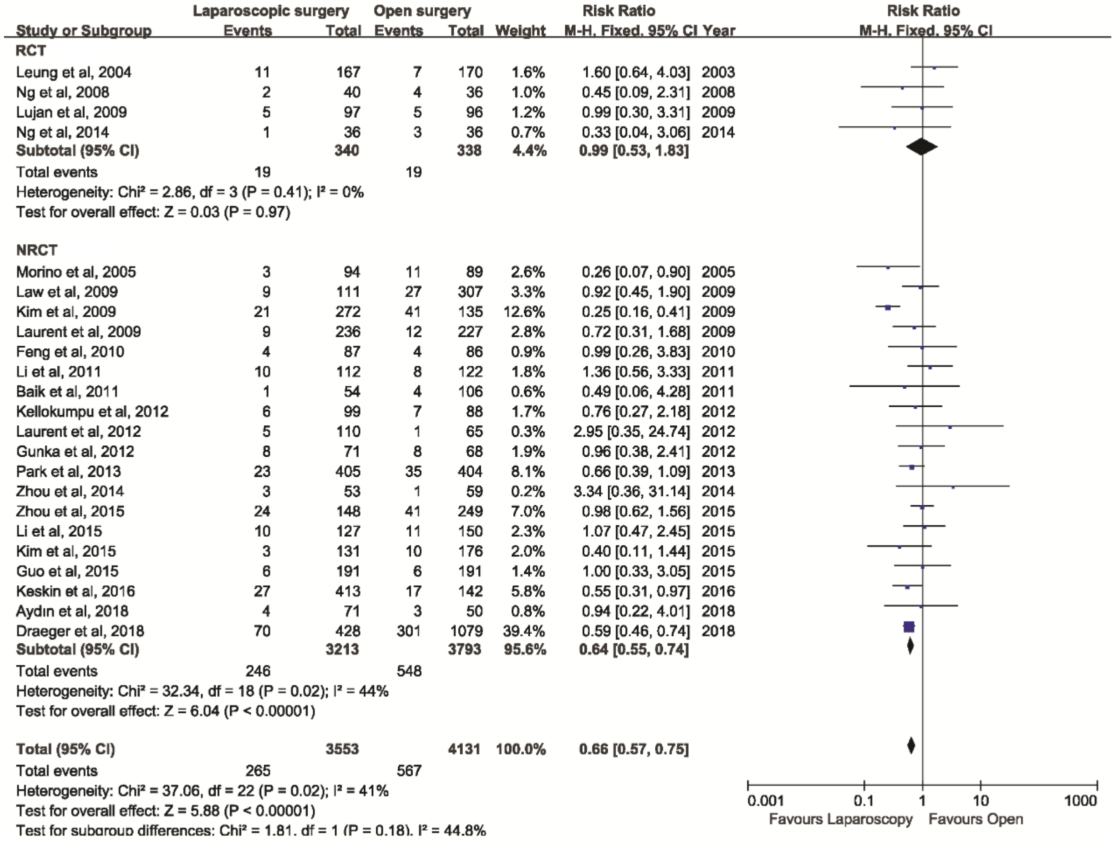

FIGURE S22 Forest plot for 3-year DFS

Study or Subgroup	Laparoscopic surgery Events	Laparoscopic surgery Total	Open surgery Events	Open surgery Total	Weight	Risk Ratio M-H, Fixed, 95% CI	Year
**RCT**							
CLASICC, 2005	47	253	21	128	5.8%	1.13 [0.71, 1.81]	2005
Braga et al, 2007	1	83	1	85	0.2%	1.02 [0.07, 16.10]	2007
COREAN, 2010	27	170	29	170	6.1%	0.93 [0.58, 1.50]	2010
ALaCaRT, 2015	44	225	39	225	8.2%	1.13 [0.76, 1.67]	2015
Z6051, 2015	35	240	37	222	8.1%	0.88 [0.57, 1.34]	2015
**Subtotal (95% CI)**		**971**		**830**	**28.4%**	**1.01 [0.82, 1.26]**	
Total events	154		127				
Heterogeneity: Chi² = 1.09, df = 4 (P = 0.90); I² = 0%							
Test for overall effect: Z = 0.13 (P = 0.90)							
**NRCT**							
Schwandner et al, 1999	4	32	5	32	1.0%	0.80 [0.24, 2.71]	1999
Leung et al, 2000	2	25	3	34	0.5%	0.91 [0.16, 5.03]	2000
Feliciotti et al, 2003	8	48	7	33	1.7%	0.79 [0.32, 1.96]	2003
Veenhof et al, 2007	5	49	10	49	2.1%	0.50 [0.18, 1.36]	2007
Staudacher et al, 2007	16	108	15	79	3.6%	0.78 [0.41, 1.48]	2007
Ströhlein et al, 2008	13	114	30	267	3.8%	1.01 [0.55, 1.87]	2008
Ding et al, 2009	9	109	14	206	2.0%	1.21 [0.54, 2.72]	2009
Park et al, 2011	14	130	15	79	3.9%	0.57 [0.29, 1.11]	2011
Seshadri et al, 2012	16	72	26	72	5.4%	0.62 [0.36, 1.05]	2012
Kusano et al, 2014	10	19	8	14	1.9%	0.92 [0.49, 1.72]	2014
Zeng et al, 2015	18	107	29	173	4.6%	1.00 [0.59, 1.72]	2015
Wang et al, 2015	3	106	12	106	2.5%	0.25 [0.07, 0.86]	2015
Kim et al, 2016	66	486	183	1095	23.6%	0.81 [0.63, 1.05]	2016
Odermatt et al, 2016	5	47	9	46	1.9%	0.54 [0.20, 1.50]	2016
Liu et al, 2016	17	58	12	39	3.0%	0.95 [0.51, 1.77]	2016
Manchon-Walsh et al, 2019	84	842	38	517	9.9%	1.36 [0.94, 1.96]	2019
**Subtotal (95% CI)**		**2352**		**2841**	**71.6%**	**0.86 [0.75, 1.00]**	
Total events	290		416				
Heterogeneity: Chi² = 16.47, df = 15 (P = 0.35); I² = 9%							
Test for overall effect: Z = 1.98 (P = 0.05)							
**Total (95% CI)**		**3323**		**3671**	**100.0%**	**0.91 [0.80, 1.02]**	
Total events	444		543				
Heterogeneity: Chi² = 19.02, df = 20 (P = 0.52); I² = 0%							
Test for overall effect: Z = 1.60 (P = 0.11)							
Test for subgroup differences: Chi² = 1.44, df = 1 (P = 0.23), I² = 30.6%							

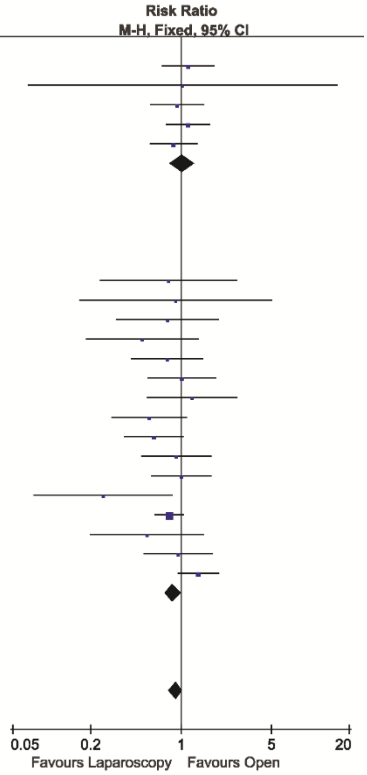

FIGURE S23 Forest plot for 3-year overall survival

Study or Subgroup	Laparoscopic surgery Events	Laparoscopic surgery Total	Open surgery Events	Open surgery Total	Weight	Risk Ratio M-H, Fixed, 95% CI	Year
**RCT**							
CLASICC, 2005	47	253	21	128	5.8%	1.13 [0.71, 1.81]	2005
Braga et al, 2007	1	83	1	85	0.2%	1.02 [0.07, 16.10]	2007
COREAN, 2010	27	170	29	170	6.1%	0.93 [0.58, 1.50]	2010
ALaCaRT, 2015	44	225	39	225	8.2%	1.13 [0.76, 1.67]	2015
Z6051, 2015	35	240	37	222	8.1%	0.88 [0.57, 1.34]	2015
**Subtotal (95% CI)**		**971**		**830**	**28.4%**	**1.01 [0.82, 1.26]**	
Total events	154		127				
Heterogeneity: Chi² = 1.09, df = 4 (P = 0.90); I² = 0%							
Test for overall effect: Z = 0.13 (P = 0.90)							
**NRCT**							
Schwandner et al, 1999	4	32	5	32	1.0%	0.80 [0.24, 2.71]	1999
Leung et al, 2000	2	25	3	34	0.5%	0.91 [0.16, 5.03]	2000
Feliciotti et al, 2003	8	48	7	33	1.7%	0.79 [0.32, 1.96]	2003
Veenhof et al, 2007	5	49	10	49	2.1%	0.50 [0.18, 1.36]	2007
Staudacher et al, 2007	16	108	15	79	3.6%	0.78 [0.41, 1.48]	2007
Ströhlein et al, 2008	13	114	30	267	3.8%	1.01 [0.55, 1.87]	2008
Ding et al, 2009	9	109	14	206	2.0%	1.21 [0.54, 2.72]	2009
Park et al, 2011	14	130	15	79	3.9%	0.57 [0.29, 1.11]	2011
Seshadri et al, 2012	16	72	26	72	5.4%	0.62 [0.36, 1.05]	2012
Kusano et al, 2014	10	19	8	14	1.9%	0.92 [0.49, 1.72]	2014
Zeng et al, 2015	18	107	29	173	4.6%	1.00 [0.59, 1.72]	2015
Wang et al, 2015	3	106	12	106	2.5%	0.25 [0.07, 0.86]	2015
Kim et al, 2016	66	486	183	1095	23.6%	0.81 [0.63, 1.05]	2016
Odermatt et al, 2016	5	47	9	46	1.9%	0.54 [0.20, 1.50]	2016
Liu et al, 2016	17	58	12	39	3.0%	0.95 [0.51, 1.77]	2016
Manchon-Walsh et al, 2019	84	842	38	517	9.9%	1.36 [0.94, 1.96]	2019
**Subtotal (95% CI)**		**2352**		**2841**	**71.6%**	**0.86 [0.75, 1.00]**	
Total events	290		416				
Heterogeneity: Chi² = 16.47, df = 15 (P = 0.35); I² = 9%							
Test for overall effect: Z = 1.98 (P = 0.05)							
**Total (95% CI)**		**3323**		**3671**	**100.0%**	**0.91 [0.80, 1.02]**	
Total events	444		543				
Heterogeneity: Chi² = 19.02, df = 20 (P = 0.52); I² = 0%							
Test for overall effect: Z = 1.60 (P = 0.11)							
Test for subgroup differences: Chi² = 1.44, df = 1 (P = 0.23), I² = 30.6%							

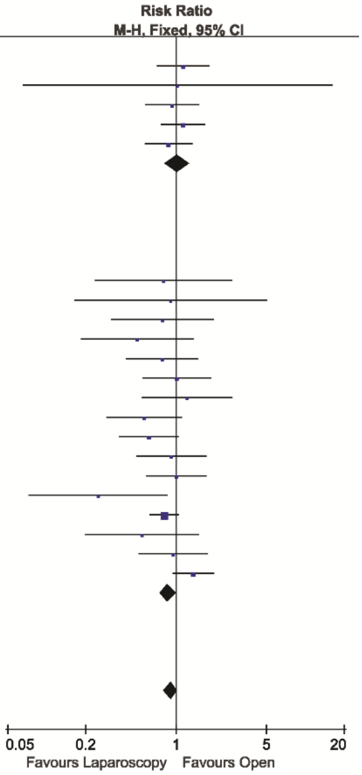

FIGURE S24 Forest plot for 5-year local recurrence

Study or Subgroup	Laparoscopic surgery Events	Laparoscopic surgery Total	Open surgery Events	Open surgery Total	Weight	Risk Ratio M-H, Fixed, 95% CI	Year
**RCT**							
CLASICC, 2005	30	253	14	128	0.7%	1.08 [0.60, 1.97]	2005
Braga et al, 2007	52	83	45	85	1.6%	1.18 [0.91, 1.54]	2007
COREAN, 2010	139	170	131	170	4.8%	1.06 [0.95, 1.18]	2010
COLOR II, 2013	518	692	244	344	11.9%	1.06 [0.97, 1.14]	2013
ALaCaRT, 2015	164	225	173	225	6.3%	0.95 [0.85, 1.06]	2015
Z6051, 2015	180	240	163	222	6.2%	1.02 [0.92, 1.14]	2015
**Subtotal (95% CI)**		**1663**		**1174**	**31.5%**	**1.04 [0.99, 1.09]**	
Total events	1083		770				
Heterogeneity: Chi² = 4.11, df = 5 (P = 0.53); I² = 0%							
Test for overall effect: Z = 1.37 (P = 0.17)							
**NRCT**							
Schwandner et al, 1999	27	32	27	32	1.0%	1.00 [0.81, 1.23]	1999
Leung et al, 2000	21	25	26	34	0.8%	1.10 [0.85, 1.41]	2000
Feliciotti et al, 2003	30	48	20	33	0.9%	1.03 [0.73, 1.47]	2003
Veenhof et al, 2007	39	49	28	49	1.0%	1.39 [1.05, 1.84]	2007
Park et al, 2011	107	130	61	79	2.8%	1.07 [0.92, 1.23]	2011
Kellokumpu et al, 2012	77	99	72	88	2.8%	0.95 [0.82, 1.10]	2012
Jefferies et al, 2012	9	12	12	16	0.4%	1.00 [0.65, 1.54]	2012
Seshadri et al, 2012	49	72	39	72	1.4%	1.26 [0.96, 1.64]	2012
Park et al, 2013	329	405	337	404	12.3%	0.97 [0.91, 1.04]	2013
Keller et al, 2014	57	61	17	19	0.9%	1.04 [0.88, 1.24]	2014
Chi et al, 2015	75	89	40	48	1.9%	1.01 [0.87, 1.18]	2015
Zeng et al, 2015	81	107	125	173	3.5%	1.05 [0.91, 1.21]	2015
Wang et al, 2015	93	106	76	106	2.8%	1.22 [1.06, 1.41]	2015
Kim et al, 2016	404	486	900	1095	20.2%	1.01 [0.96, 1.06]	2016
Nonaka et al, 2016	32	40	24	38	0.9%	1.27 [0.95, 1.69]	2016
Odermatt et al, 2016	34	47	26	46	1.0%	1.28 [0.94, 1.74]	2016
De'Angelis et al, 2017	27	49	25	46	0.9%	1.01 [0.70, 1.46]	2017
Wu et al, 2018	213	277	486	614	11.0%	0.97 [0.90, 1.05]	2018
Chen et al, 2019	58	64	15	23	0.8%	1.39 [1.02, 1.89]	2019
Zhang X et al, 2019	59	86	26	39	1.3%	1.03 [0.79, 1.34]	2019
**Subtotal (95% CI)**		**2284**		**3054**	**68.5%**	**1.03 [1.00, 1.06]**	
Total events	1821		2382				
Heterogeneity: Chi² = 27.84, df = 19 (P = 0.09); I² = 32%							
Test for overall effect: Z = 2.14 (P = 0.03)							
**Total (95% CI)**		**3947**		**4228**	**100.0%**	**1.03 [1.01, 1.06]**	
Total events	2904		3152				
Heterogeneity: Chi² = 32.02, df = 25 (P = 0.16); I² = 22%							
Test for overall effect: Z = 2.53 (P = 0.01)							
Test for subgroup differences: Chi² = 0.01, df = 1 (P = 0.93), I² = 0%							

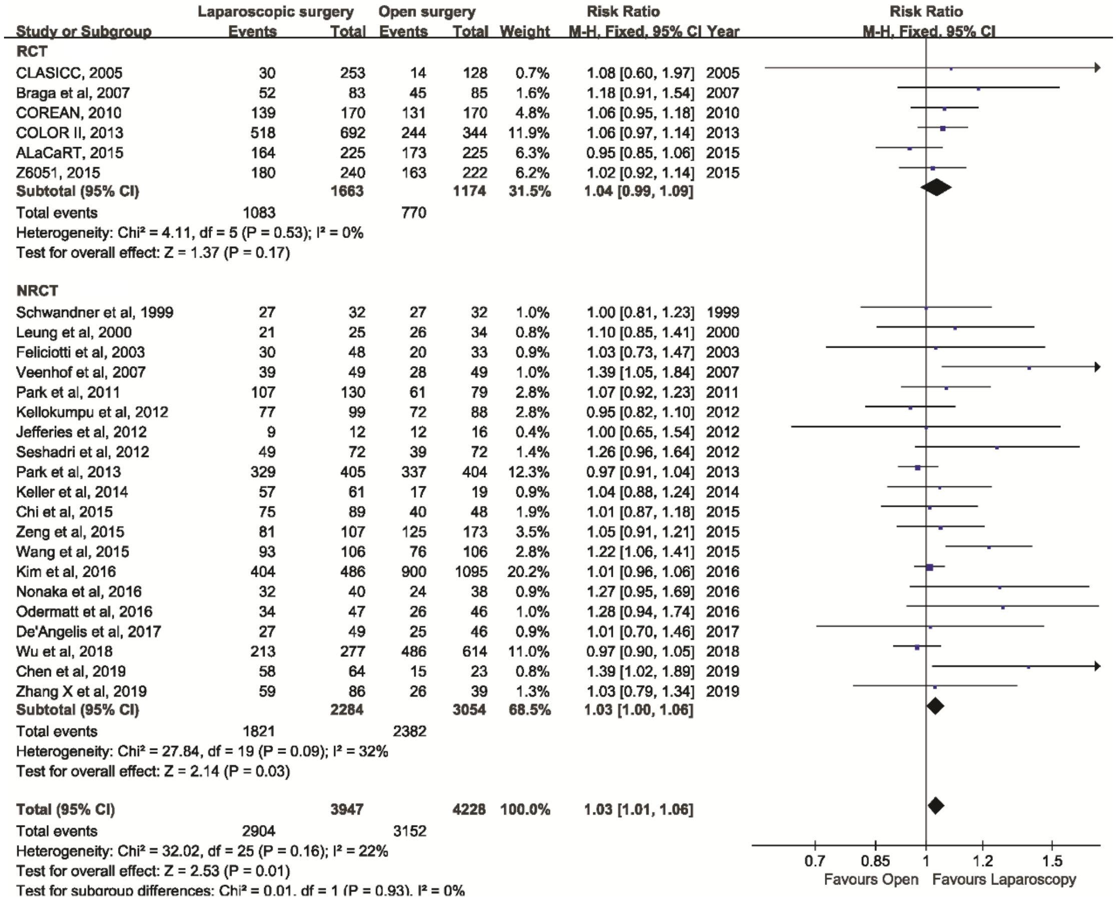

FIGURE S25 Forest plot for 5-year distant recurrence

	Laparoscopic surgery		Open surgery			Risk Ratio	
**Study or Subgroup**	**Events**	**Total**	**Events**	**Total**	**Weight**	**M-H, Random, 95% CI**	**Year**
**RCT**							
Leung et al, 2004	126	167	133	170	5.4%	0.96 [0.86, 1.08]	2003
CLASICC, 2005	135	253	67	128	2.5%	1.02 [0.83, 1.25]	2005
Ng et al, 2008	31	40	26	36	1.6%	1.07 [0.83, 1.40]	2008
Lujan et al, 2009	82	97	78	96	4.8%	1.04 [0.92, 1.18]	2009
Ng et al, 2014	30	36	27	36	1.9%	1.11 [0.88, 1.41]	2014
**Subtotal (95% CI)**		**593**		**466**	**16.2%**	**1.02 [0.95, 1.09]**	
Total events	404		331				
Heterogeneity: Tau² = 0.00; Chi² = 1.60, df = 4 (P = 0.81); I² = 0%							
Test for overall effect: Z = 0.44 (P = 0.66)							
**NRCT**							
Morino et al, 2005	61	94	52	93	2.0%	1.16 [0.92, 1.47]	2005
Laurent et al, 2009	194	236	179	227	7.1%	1.04 [0.95, 1.14]	2009
Kim et al, 2009	186	272	70	135	3.0%	1.32 [1.10, 1.58]	2009
Feng et al, 2010	68	87	64	86	3.4%	1.05 [0.89, 1.24]	2010
Baik et al, 2011	44	54	81	106	3.4%	1.07 [0.90, 1.26]	2011
Laurent et al, 2012	77	110	46	65	2.6%	0.99 [0.81, 1.21]	2012
Siani et al, 2012	24	30	24	30	1.7%	1.00 [0.78, 1.29]	2012
Seshadri et al, 2012	31	51	21	44	0.8%	1.27 [0.87, 1.86]	2012
Park et al, 2013	314	405	320	404	8.4%	0.98 [0.91, 1.05]	2013
Lee et al, 2013	76	79	79	80	10.2%	0.97 [0.93, 1.02]	2013
Zhou et al, 2015	93	148	157	249	3.7%	1.00 [0.85, 1.17]	2015
Guo et al, 2015	124	191	117	191	3.8%	1.06 [0.91, 1.24]	2015
Keskin et al, 2016	325	413	97	142	5.1%	1.15 [1.02, 1.30]	2016
Aydın et al, 2018	59	71	37	50	2.6%	1.12 [0.92, 1.36]	2018
Draeger et al, 2018	331	428	735	1079	8.9%	1.14 [1.06, 1.21]	2018
Zimmermann et al, 2019	127	168	116	160	4.8%	1.04 [0.92, 1.19]	2019
Schnitzbauer et al, 2019	3651	4463	8523	11471	12.4%	1.10 [1.08, 1.12]	2019
**Subtotal (95% CI)**		**7300**		**14612**	**83.8%**	**1.07 [1.02, 1.11]**	
Total events	5785		10718				
Heterogeneity: Tau² = 0.00; Chi² = 41.86, df = 16 (P = 0.0004); I² = 62%							
Test for overall effect: Z = 3.07 (P = 0.002)							
**Total (95% CI)**		**7893**		**15078**	**100.0%**	**1.06 [1.02, 1.10]**	
Total events	6189		11049				
Heterogeneity: Tau² = 0.00; Chi² = 45.96, df = 21 (P = 0.001); I² = 54%							
Test for overall effect: Z = 3.10 (P = 0.002)							
Test for subgroup differences: Chi² = 1.23, df = 1 (P = 0.27), I² = 18.8%							

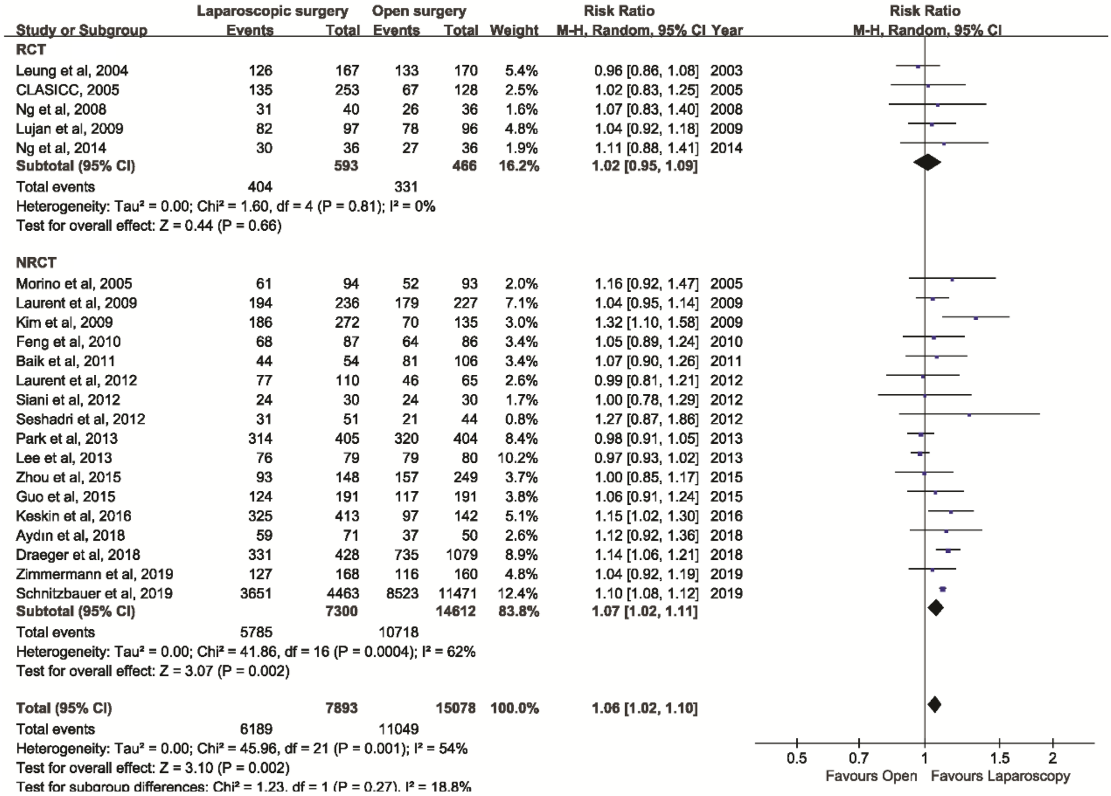

FIGURE S26 Forest plot for 5-year disease free survival

Study or Subgroup	Laparoscopic surgery Events	Laparoscopic surgery Total	Open surgery Events	Open surgery Total	Weight	Risk Ratio M-H, Random, 95% CI	Year
**RCT**							
Leung et al, 2004	126	167	133	170	5.4%	0.96 [0.86, 1.08]	2003
CLASICC, 2005	135	253	67	128	2.5%	1.02 [0.83, 1.25]	2005
Ng et al, 2008	31	40	26	36	1.6%	1.07 [0.83, 1.40]	2008
Lujan et al, 2009	82	97	78	96	4.8%	1.04 [0.92, 1.18]	2009
Ng et al, 2014	30	36	27	36	1.9%	1.11 [0.88, 1.41]	2014
**Subtotal (95% CI)**		**593**		**466**	**16.2%**	**1.02 [0.95, 1.09]**	
Total events	404		331				
Heterogeneity: Tau² = 0.00; Chi² = 1.60, df = 4 (P = 0.81); I² = 0%							
Test for overall effect: Z = 0.44 (P = 0.66)							
**NRCT**							
Morino et al, 2005	61	94	52	93	2.0%	1.16 [0.92, 1.47]	2005
Laurent et al, 2009	194	236	179	227	7.1%	1.04 [0.95, 1.14]	2009
Kim et al, 2009	186	272	70	135	3.0%	1.32 [1.10, 1.58]	2009
Feng et al, 2010	68	87	64	86	3.4%	1.05 [0.89, 1.24]	2010
Baik et al, 2011	44	54	81	106	3.4%	1.07 [0.90, 1.26]	2011
Laurent et al, 2012	77	110	46	65	2.6%	0.99 [0.81, 1.21]	2012
Siani et al, 2012	24	30	24	30	1.7%	1.00 [0.78, 1.29]	2012
Seshadri et al, 2012	31	51	21	44	0.8%	1.27 [0.87, 1.86]	2012
Park et al, 2013	314	405	320	404	8.4%	0.98 [0.91, 1.05]	2013
Lee et al, 2013	76	79	79	80	10.2%	0.97 [0.93, 1.02]	2013
Zhou et al, 2015	93	148	157	249	3.7%	1.00 [0.85, 1.17]	2015
Guo et al, 2015	124	191	117	191	3.8%	1.06 [0.91, 1.24]	2015
Keskin et al, 2016	325	413	97	142	5.1%	1.15 [1.02, 1.30]	2016
Aydın et al, 2018	59	71	37	50	2.6%	1.12 [0.92, 1.36]	2018
Draeger et al, 2018	331	428	735	1079	8.9%	1.14 [1.06, 1.21]	2018
Zimmermann et al, 2019	127	168	116	160	4.8%	1.04 [0.92, 1.19]	2019
Schnitzbauer et al, 2019	3651	4463	8523	11471	12.4%	1.10 [1.08, 1.12]	2019
**Subtotal (95% CI)**		**7300**		**14612**	**83.8%**	**1.07 [1.02, 1.11]**	
Total events	5785		10718				
Heterogeneity: Tau² = 0.00; Chi² = 41.86, df = 16 (P = 0.0004); I² = 62%							
Test for overall effect: Z = 3.07 (P = 0.002)							
**Total (95% CI)**		**7893**		**15078**	**100.0%**	**1.06 [1.02, 1.10]**	
Total events	6189		11049				
Heterogeneity: Tau² = 0.00; Chi² = 45.96, df = 21 (P = 0.001); I² = 54%							
Test for overall effect: Z = 3.10 (P = 0.002)							
Test for subgroup differences: Chi² = 1.23, df = 1 (P = 0.27), I² = 18.8%							

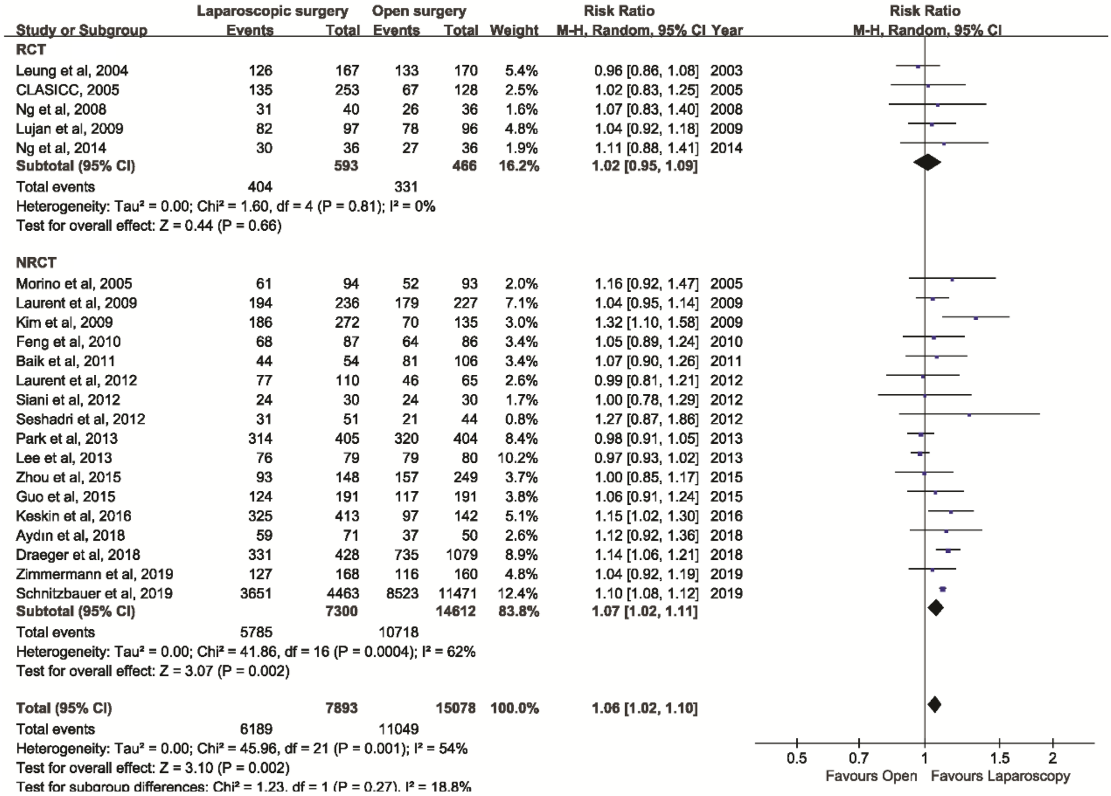

FIGURE S27 Forest plot for 5-year overall survival

Study or Subgroup	Laparoscopic surgery Events	Laparoscopic surgery Total	Open surgery Events	Open surgery Total	Weight	Risk Ratio M-H, Random, 95% CI	Year
**RCT**							
Leung et al, 2004	127	167	124	170	3.4%	1.04 [0.92, 1.18]	2003
CLASICC, 2005	142	253	60	128	1.7%	1.20 [0.97, 1.48]	2005
Ng et al, 2008	30	40	28	36	1.4%	0.96 [0.75, 1.24]	2008
Lujan et al, 2009	70	97	72	96	2.4%	0.96 [0.81, 1.14]	2009
Ng et al, 2014	31	36	33	36	2.5%	0.94 [0.80, 1.11]	2014
**Subtotal (95% CI)**		**593**		**466**	**11.5%**	**1.01 [0.93, 1.10]**	
Total events	400		317				
Heterogeneity: Tau² = 0.00; Chi² = 4.48, df = 4 (P = 0.34); I² = 11%							
Test for overall effect: Z = 0.32 (P = 0.75)							
**NRCT**							
Morino et al, 2005	75	94	61	89	2.3%	1.16 [0.98, 1.38]	2005
Kim et al, 2009	194	272	73	135	2.4%	1.32 [1.11, 1.57]	2009
Law et al, 2009	79	111	182	307	2.8%	1.20 [1.03, 1.40]	2009
Laurent et al, 2009	196	236	163	227	4.2%	1.16 [1.05, 1.28]	2009
Li et al, 2011	87	112	96	122	3.1%	0.99 [0.86, 1.13]	2011
Baik et al, 2011	50	54	94	106	4.1%	1.04 [0.94, 1.16]	2011
Laurent et al, 2012	94	110	53	65	3.1%	1.05 [0.91, 1.20]	2012
Kellokumpu et al, 2012	70	99	61	88	2.1%	1.02 [0.85, 1.23]	2012
Li et al, 2012	252	369	180	267	3.9%	1.01 [0.91, 1.13]	2012
Seshadri et al, 2012	34	51	26	44	0.9%	1.13 [0.82, 1.54]	2012
Siani et al, 2012	25	30	24	30	1.5%	1.04 [0.82, 1.32]	2012
Park et al, 2013	333	405	328	404	5.4%	1.01 [0.95, 1.08]	2013
Lee et al, 2013	77	79	79	80	6.1%	0.99 [0.95, 1.03]	2013
Zhou et al, 2014	50	53	54	59	4.1%	1.03 [0.93, 1.14]	2014
Huang et al, 2015	377	490	317	422	5.1%	1.02 [0.95, 1.10]	2015
Li et al, 2015	93	127	114	150	3.1%	0.96 [0.84, 1.11]	2015
Zhou et al, 2015	101	148	158	249	2.9%	1.08 [0.93, 1.24]	2015
Kim et al, 2015	111	131	128	176	3.7%	1.17 [1.04, 1.31]	2015
Guo et al, 2015	134	191	126	191	3.1%	1.06 [0.93, 1.22]	2015
Keskin et al, 2016	351	413	101	142	3.8%	1.19 [1.07, 1.34]	2016
Draeger et al, 2018	310	428	890	1079	5.4%	0.88 [0.82, 0.94]	2018
Schnitzbauer et al, 2019	3686	4463	8787	11471	6.7%	1.08 [1.06, 1.10]	2019
Manchon-Walsh et al, 2019	651	842	335	517	5.1%	1.19 [1.11, 1.28]	2019
Zimmermann et al, 2019	134	168	126	160	3.8%	1.01 [0.91, 1.13]	2019
**Subtotal (95% CI)**		**9476**		**16580**	**88.5%**	**1.06 [1.02, 1.10]**	
Total events	7564		12556				
Heterogeneity: Tau² = 0.00; Chi² = 86.08, df = 23 (P < 0.00001); I² = 73%							
Test for overall effect: Z = 3.25 (P = 0.001)							
**Total (95% CI)**		**10069**		**17046**	**100.0%**	**1.05 [1.02, 1.09]**	
Total events	7964		12873				
Heterogeneity: Tau² = 0.00; Chi² = 91.47, df = 28 (P < 0.00001); I² = 69%							
Test for overall effect: Z = 3.19 (P = 0.001)							
Test for subgroup differences: Chi² = 1.01, df = 1 (P = 0.32), I² = 0.6%							

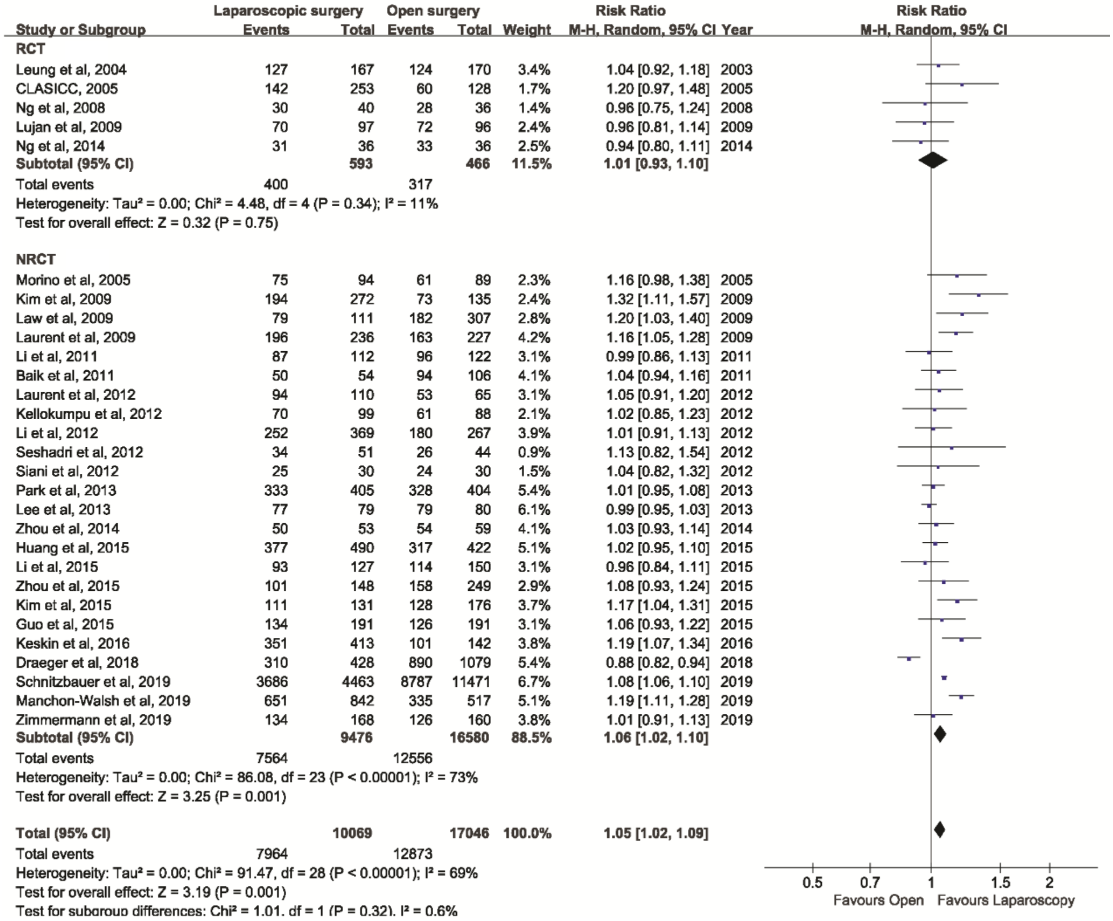

Supplement: Supplementary file 1 — Figure S1. [file CAM4-13-e7363-s001.pdf]
